# Specialized pericyte subtypes in the pulmonary capillaries

**DOI:** 10.1038/s44318-024-00349-1

**Published:** 2025-01-13

**Authors:** Timothy Klouda, Yunhye Kim, Seung-Han Baek, Mantu Bhaumik, Yan Li, Yu Liu, Joseph C Wu, Benjamin A Raby, Vinicio de Jesus Perez, Ke Yuan

**Affiliations:** 1 Division of Pulmonary Medicine, Boston Children’s Hospital, Boston, MA 02115 USA; 2 Department of Neurology, F.M. Kirby Neurobiology Center, Boston Children’s Hospital and Harvard Medical School, Boston, MA USA; 3 Stanford Cardiovascular Institute, Division of Cardiovascular Medicine, Department of Medicine, Stanford University School of Medicine, Stanford, CA 94304 USA; 4 Division of Pulmonary and Allergy Critical Care Medicine, School of Medicine, Stanford University, Palo Alto, CA USA

**Keywords:** Pericytes, Higd1b, Capillary, Pulmonary Hypertension, Single-cell RNA Sequence, Chromatin, Transcription & Genomics, Development, Vascular Biology & Angiogenesis

## Abstract

Pericytes are essential for capillary stability and homeostasis, with impaired pericyte function linked to diseases like pulmonary arterial hypertension. Investigating pericyte biology has been challenging due to the lack of specific markers, making it difficult to distinguish pericytes from other stromal cells. Using bioinformatic analysis and RNAscope, we identified *Higd1b* as a unique gene marker for pericytes and subsequently generated a knock-in mouse line, *Higd1b*-CreERT2, that accurately labels pericytes in the lung and heart. Single-cell RNA sequencing revealed two distinct *Higd1b*+ pericyte subtypes: while Type 1 pericytes support capillary homeostasis, Type 2 pericytes accumulate in arterioles, and co-express smooth muscle markers and higher levels of vimentin under hypoxic conditions. Lastly, healthy human lung pericytes with upregulation of vimentin exhibited increased adhesion, migration, and higher expression levels of the smooth muscle marker SM22 in vitro. These findings highlight the specialization of pulmonary pericytes and their contribution to vascular remodeling during hypoxia-induced pulmonary hypertension.

## Synopsis

While pericytes are important regulators of capillary function and homeostasis, the lack of specific markers has hampered the investigation of pericyte biology. This study identifies Higd1b as a unique and specific gene marker for pulmonary pericytes, enabling the identification of two pericyte subtypes and their contribution to vascular remodeling in hypoxia-induced pulmonary hypertension.

- *HIGD1B* is a unique gene marker for pulmonary pericytes that is more specific than *CSPG4* and *PDGFRB*.
- *Higd1b*-CreERT2 knock-in mice allow fate mapping experiments for pericytes in the lung, heart, and other organs.
- Single-cell RNAseq analysis in human and murine lungs suggests the presence of two *HIGD1B*+ pericyte subtypes.
- Upon hypoxia, type 1 pericytes remain quiescent in the capillary network, while type 2 PCs upregulate vimentin and smooth muscle cell marker expression and accumulate in the arterioles.

## Abstract (web summary)

Identification of *Higd1b* as a specific marker of pulmonary pericytes enables the identification of pericyte subtypes with distinct responses to hypoxia-induced pulmonary hypertension.

## Graphical abstract

**Figure:**
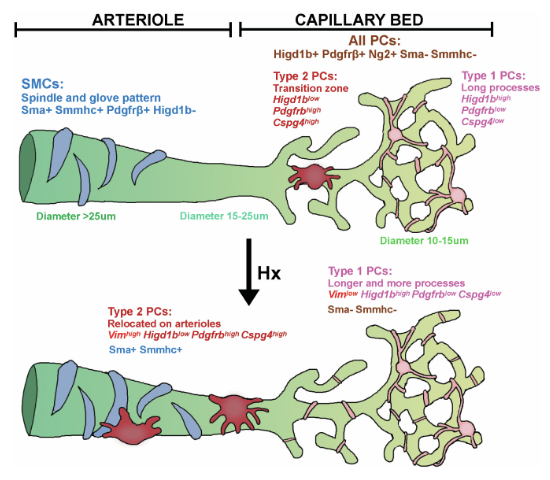

**Figure:**
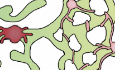

## Introduction

Pericytes (PCs) are mural cells that reside in the circulatory system’s microvasculature and maintain direct contact with capillary endothelial cells (ECs) (Allt and Lawrenson, 2001). Embedded within the basement membrane, they have critical roles in capillary homeostasis, angiogenesis, immune surveillance, and vessel maturation (Chatterjee and Naik, 2012; Fuxe et al, 2011; Gaengel et al, 2009; Gerhardt and Betsholtz, 2003). The loss of PC function or coverage is associated with numerous diseases, including Alzheimer’s, diabetic retinopathy, cancer, and pulmonary arterial hypertension (PAH) (Halliday et al, 2016; Hammes et al, 2002; Yuan et al, 2020). Despite their involvement in pathological processes across multiple organ systems, PCs’ contribution to disease development is elusive in many circumstances. A significant limitation in studying PC biology is the lack of a specific and unique cell marker, making it difficult to distinguish them from other mural cell populations under physiological and pathological conditions (Yuan et al, 2021).

Commonly used cell markers to identify PCs include Chondroitin Sulfate Proteoglycan 4 (*CSPG4*, aka neuroglial antigen NG2) and Platelet-Derived Growth Factor Receptor-β (*PDGFRB* aka CD140b) (Schiffer et al, 2018; Winkler et al, 2010). However, these markers lack specificity and uniqueness to PCs, as they are co-expressed in various cell types across multiple organ systems, including vascular smooth muscle cells (SMCs) expressing *ACTA2* and *TAGLN*, fibroblasts (FBs) expressing *DCN, COL1A1*, and *FBLN1*, and oligodendrocytes expressing *OLIG2* (Chen et al, 2011; Jin et al, 2008; Ligon et al, 2006; Muhl et al, 2020). To distinguish PCs from other mural cell populations, investigators rely on these cell markers combined with the PC’s distinct morphology (oval cell bodies with multiple, thin, punctate and elongated branches/processes) and location in the circulatory system (abundantly encircling capillaries with a small population residing on the capillary-arterioles and capillary venule borders) (Armulik et al, 2011; Yuan et al, 2021; Yuan et al, 2020). Recent advances in single-cell RNA sequencing (scRNA-seq) have revealed organ-specific gene expression in PCs, reflecting their multifaceted functions throughout the body (Baek et al, 2022). Due to these limitations, there is a critical need to identify and validate a PC-specific cell marker.

PCs play a crucial role in supporting the circulatory system and exhibit multipotent stem cell-like properties (Ahmed and El-Badri, 2018; Courtney and Sutherland, 2020). A notable histopathological feature of PAH is the excessive proliferation and accumulation of SMCs in the distal arterioles (Stenmark et al, 2018). Our group has demonstrated that in response to chronic hypoxia (Hx), Ng2+ mural cells contribute to developing pulmonary hypertension (PH) and vascular remodeling by accumulating in the microvasculature and transitioning into SMC-like cells (Yuan et al, 2020). PCs’ adaptability in response to Hx and organ injury positions them as promising targets for cell-directed therapies, not only in PAH, but also in other disease processes (Mills et al, 2013). Through analysis of murine and human scRNA-seq databases, our group has previously identified HIG1 hypoxia-inducible domain family member 1B (*Higd1b*) as a gene exclusively expressed in *Cspg4*+*/Pdgfrβ+* mural cells in hearts and lungs (Baek et al, 2022).

In this study, we utilize additional scRNA-seq data and spatial transcriptomic analysis from human and murine lungs and hearts to identify *Higd1b* as a PC-specific gene marker. We employed the Cre-LoxP and CRISPR techniques to construct a novel, tamoxifen-inducible *Higd1b*-CreERT2 mouse model. Validation with reporter lines (*R26-tdTomato* and *R26-mTmG*) confirmed *Higd1b*-*Cre*+ cells effectively labeled PCs in abundance in the lung and heart, also labeling cells in the brain, skeletal muscle, pancreas, intestine, connective tissue, kidney, and retina. Lastly, through lineage tracing studies, we demonstrated that two subtypes of PCs marked by *Higd1b* exist in the pulmonary circulation. Type 1 PCs are quiescent on capillaries, and Type 2 PCs exhibit multipotent properties and accumulate in the arterioles after exposure to Hx. With cell culture experiments, we provide evidence that PCs exhibit higher adhesion, motility, and increased levels of Transgelin (aka SM22) after Vimentin overexpression, thereby contributing to smooth muscle contraction and vascular remodeling. This discovery suggests *Higd1b* is a more specific cell marker for studying PCs in cardiorespiratory and vascular diseases. In addition, identifying PC subtypes in both humans and rodents will empower future investigators to delve into the dynamic role of PCs in disease development and pave the way for disease-modifying therapies targeting specific subgroups of PCs.

## Results

### *HIGD1B*/*Higd1b* is identified as a unique and exclusive marker for human and murine lung PCs

Using murine and human scRNA-seq data from multiple tissue types, we identified several PC organ-specific markers, including *Kcnk3* (lung), *Rgs4* (heart), and *Higd1*b (lung and heart), whose expression was restricted to annotated stringent PC clusters co-expressing both *Cspg4 and Pdgfrb* (Baek et al, 2022). Of these candidates, *Higd1b* was particularly interesting because its expression was conserved in human lung and heart tissues. We speculated that *Higd1b* would distinguish human and murine PCs from other mural cell populations in the cardiovascular system.

**Figure 1:**
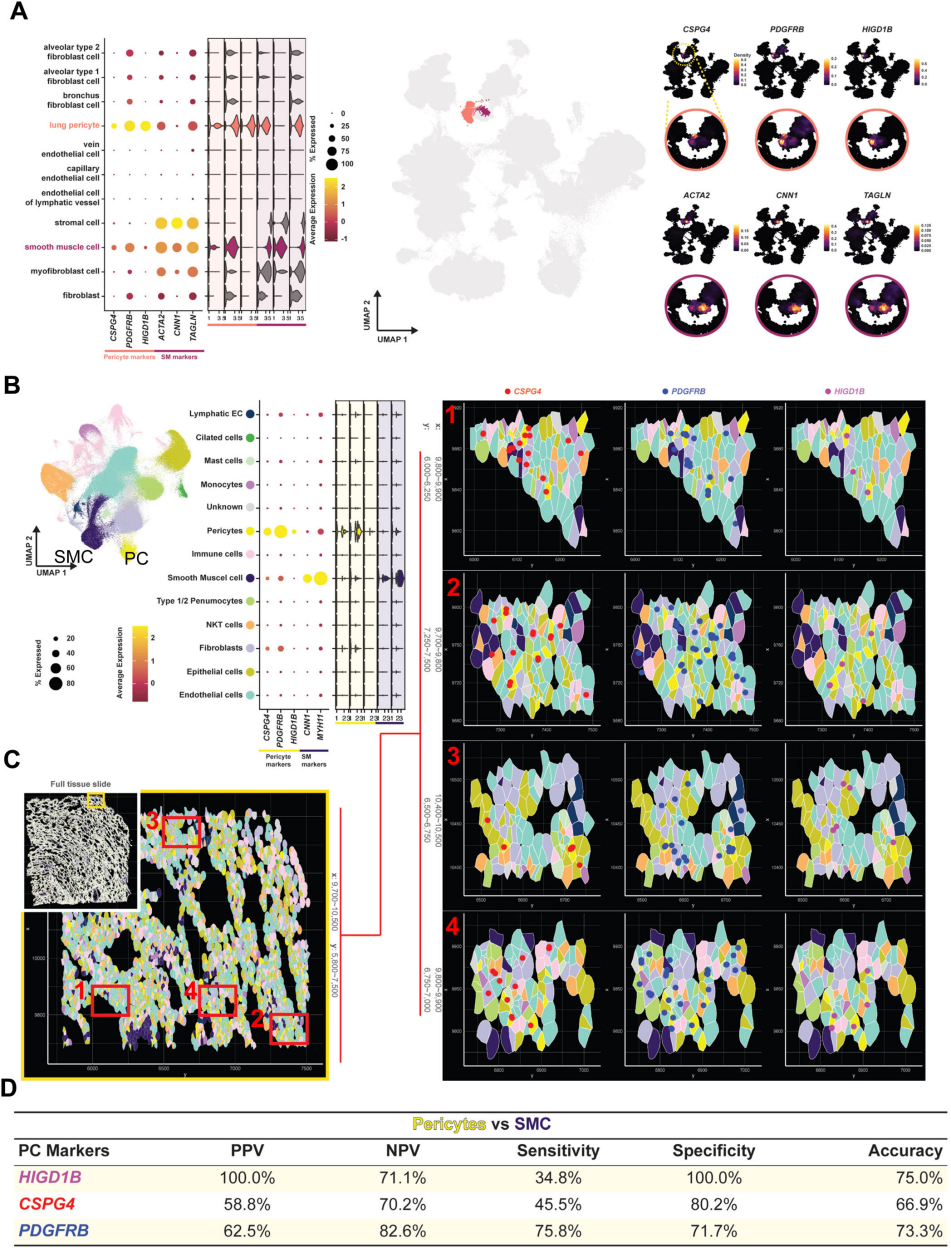
*HIGD1B* is identified as a unique and exclusive marker for human lung PCs. (**A**) Dot, Violin, and Density plots show the expression of PC (*CSPG4, PDGFRB*, and *HIGD1B*) and SMC markers (*ACTA2*, *CNN1*, and *TAGLN*) across eleven selected cell types from the Human Lung Cell Atlas (HLCA). Note the exclusive expression of *HIGD1B* in PCs compared to other mural and vascular cells. On the right, a UMAP visualization illustrates PC distributions within lung tissues, using original UMAP coordinates and cell annotations from the ‘HLCA (core)’. The Density plot presents relative expression of selected PC and SMC markers within the PC and SMC populations. In the UMAP, Dot, and Violin plots, PCs were highlighted in light orange and SMCs in dark red. Comprehensive expression data across all annotated cell types were presented in Fig. EV1. (**B**) UMAP visualization of annotated cell type clusters using the ‘Xenium Human Lung Preview Data (Non-diseased Lung)’, derived from the spatial transcriptomic analysis. Dot and Violin plots show the expression of PC markers (*CSPG4*, *PDGFRB*, and *HIGD1B*) and SMC markers (*CNN1* and *MYH11*) across thirteen annotated cell types. Consistent with the HLCA, *HIGD1B* had exclusive expression in PCs compared to other mural and vascular cells. ‘FindAllMarkers’ function of the Seurat pipeline was utilized to identify cell type markers for each cluster to annotate cell types. (**C**) One of three selected areas (yellow box) from the full lung tissue slide used for spatial transcriptomic quantifications. Four images (red boxes #1–4) were selected and inspected under higher magnification. PCs were annotated in yellow, SMCs in dark blue, and other cell types follow the color-code provided in the dot plot in (**B**). The expression of *CSPG4* is shown as red dots (left), *PDGFRB* as blue dots (middle), and *HIGD1B* as purple dots (right). Comprehensive quantifications across all inspected areas are presented in Appendix Fig. S7 and Fig. EV2. (**D**) The table summarizes the PPV, NPV, Sensitivity, Specificity, and Accuracy of *HIGD1B, CSPG4*, and *PDGFRB* to distinguish PCs from SMCs in spatial transcriptomic images across all inspected areas (Fig. EV2). PPV positive predictive value, NPV negative predictive value. Comprehensive 2 × 2 contingency tables used for quantifications are presented in Fig. EV2.

To further explore *HIGD1B* as a potential marker for PCs in lung and heart tissues, we examined the expression of *CSPG4*, *PDGFRB*, and *HIGD1B* in the human (Human Lung Cell Atlas Core) and murine (Tabula Muris Senis) single-cell data. The Human Lung Cell Atlas is a publicly accessible database comprising scRNA-seq data on 584,944 human lung cells and circulating blood collected from 107 healthy individuals (Travaglini et al, 2020). The Tabula Muris Senis compendium (https://tabula-muris.ds.czbiohub.org/) is a publicly available single-cell transcriptomic atlas covering the lifespan of *Mus musculus*, including data from 23 tissues and organs. Using the original UMAP coordinates and cell type annotations, we mapped the annotated PCs onto a UMAP to assess the expressing levels of common PC markers *CSPG4* (*Cspg4*), *PDGFRB* (*Pdgfrb*), and *HIGD1B* (*Higb1b*) in both human and murine scRNA-seq lung data. Expression patterns were visualized using Dot, Violin, and Density plots (Figs. 1A and EV1; Appendix Fig. S1). In human lungs, *HIGD1B* expression was found exclusive to PCs, whereas *PDGFRB* was expressed in PCs at similar levels but also present in multiple FB subtypes, indicating broader cellular distribution. *CSPG4* was predominantly expressed in PCs and SMCs, but its expression was reduced by about 40% compared to *HIGD1B*. Markers such as *ACTA2, CNN1*, and *TAGLN* were significantly expressed in mural SMCs (Fig. 1A). The expression patterns of *CSPG4*, *PDGFRB*, *HIGD1B, ACTA2, CNN1*, and *TAGLN* across all annotated human lung cell types are detailed in Fig. EV1A. Differential expression (DE) analysis in PCs (*n* = 3032) compared to all other annotated cells showed that 85.6% of PCs expressed *HIGD1B*, with a significant 25-fold increase compared to 0.5% of all other cells (adjusted *p* < 1E−04). *PDGFRB* was expressed in 91.1% of the PC population but also a 7-fold increase in SMCs, further highlighting its non-specificity for PCs and broad expression across stromal cells (Fig. EV1B).

**Figure EV1:**

*HIGD1B* is expressed in human lung PCs. (**A**) UMAP visualization of cell populations within human lung tissue using the original UMAP coordinates and cell type annotations from the ‘Human Lung Cell Atlas (core)’. Cell type annotations are color-coded, as shown in the dot plot. PC and SMC distributions within lung tissues, using original UMAP coordinates and cell annotations from the ‘HLCA (core)’ are highlighted in the bottom left. In UMAP, Dot, Violin and Heatmap plots, PCs were highlighted in light orange and SMCs in dark red. The Dot and Violin plots on the right illustrate the expression pattern of PC (*CSPG4, PDGFRB*, and *HIGD1B*) and SMC markers (*ACTA2*, *CNN1*, and *TAGLN*) across all annotated cell types. The Dot plot highlights the relative expression of markers, while the Violin plot depicts the distribution and intensity of expression within each cell type. (**B**) Differential expression (DE) analysis utilizing the Wilcoxon rank-sum test shows the expression profiles of PC markers (*CSPG4, PDGFRB*, and *HIGD1B*) and SMC markers (*ACTA2*, *CNN1*, and *TAGLN*) in annotated PCs and SMCs compared to all other cell populations within the ‘Human Lung Cell Atlas (core)’.

**Figure EV2:**

Statistical analysis of spatial transcriptomic figures are collected from non-diseased lung tissue. (**A**) The quantification of mural cell markers was performed using four selected areas from the spatial transcriptomic figure, derived from the three yellow boxes in Appendix Fig. S7. In these analyses, PCs are represented by yellow-colored cells, while SMCs are indicated by dark blue-colored cells. The expression of specific markers is depicted as follows: *CSPG4* is represented by red dots (left), *PDGFRB* by blue dots (middle), and *HIGD1B* by purple dots (right). (**B**) The analysis results are summarized in 2 × 2 tables that display the presence of *CSPG4*, *PDGFRB*, and *HIGD1B* across cell populations. Detailed quantification processes are described in the Methods section. Each table is followed by the corresponding results of the analysis. (**C**) The bottom tables summarize the performance of each marker to distinguish PCs or SMCs from other cells (PC vs all other cells, SMCs vs all other cells, and PCs vs SMCs). TP true positive, FP false positive, TN true negative, PPV positive predictive value, NPV negative predictive value.

A similar pattern of *Higd1b* expression was observed in the *Tabula Muris* Senis data, where *Higd1b* was exclusively expressed in PCs without labeling other mural cells. On the other hand, *Pdgfrb* was highly expressed in the PC population but also more broadly expressed across other stromal cells. *Cspg4* was specific to PCs but at significantly lower levels (Appendix Fig. S1A,B). DE gene analysis revealed that 100% of murine pulmonary PCs expressed *Higd1b*, reinforcing its specificity for PCs (Appendix Fig. S1C). For cardiac tissues, the human heart data consists of scRNA-seq on 486,134 cells from all heart compartments and 14 individuals of both sexes, ranging in age from 40 to 75 (Litviňuková et al, 2020). The mouse heart data is from the C57BL/6 wild-type mouse strain, which consists of 25,436 cardiac cells (Feng et al, 2022). In both human and mouse heart scRNA-seq data, *CSPG4/Cspg4*, *PDGFRB/Pdgfrb*, and *HIGD1B/Higb1b* demonstrated relatively high expression within PC populations, as shown in the Dot, Violin, and Density plots (Appendix Figs. S2A,B and S3A,B). However, *HIGD1B/Higd1b* expression was not entirely exclusive to PCs, as a subset of SMCs also expressed it. Notably, *Higd1b*’s expression was more specific to mouse heart PCs than *Cspg4* and *Pdgfrb* (Appendix Figs. S2C and S3C). An overview of the DE gene analysis across all four scRNA-seq data is presented in Appendix Fig. S4, highlighting the high expression of *HIGD1B/Higd1b* in human lungs, murine lungs, and hearts.

To further investigate the spatial distribution of PCs within lung tissue, we analyzed spatial transcriptomic data from the ‘Xenium Human Lung Preview Data (Non-diseased Lung)’ provided by 10x Genomics. This data, which consists of 295,883 cells, was used to examine the expression patterns of *CSPG4*, *PDGFRB*, and *HIGD1B*. The 23 annotated cell types were mapped to the UMAP visualization and the tissue sample of 295,883 cells (Fig. 1B; Appendix Fig. S5). Since *ACTA2* and *TAGLN* were not included in the spatial transcriptomic data gene panelists from the raw data, *MYH11* expression was used to identify SMCs. A consensus-based approach was employed to refine cell type annotations (Appendix Fig. S6).

After identifying cell types, three sections (size: 1000 × 2500) from the original image were randomly selected (coordinates: x: 3750–4550, y: 1200–2900; x: 5200–6000, y: 3300–5000; x: 9700–10500, y: 5800–7500) for analysis (Fig. 1C; Appendix Fig. S7). Four random regions were selected from each of the three sections for quantifications (Figs. 1C and EV2A). Cells of interest, including PCs (yellow), SMCs (dark purple), and other cell populations, were color-coded according to their annotated cell types. The expression of mural cell markers *CSPG4* (red)*, PDGFRB* (blue), and *HIGD1B* (purple) was also color-coded. In the twelve regions examined, a total of 1312 cells were identified, of which 66 were PCs (PC+ column) and 106 SMCs (SMC+ column). *HIGD1B* was identified on 49 cells (*HIGD1B*+ row), *CSPG4* on 105 cells (*CSPG4*+ row), and *PDGFRB* on 229 cells (*PDGFRB+* row) (Fig. EV2B, top and middle 2 × 2 tables). We then calculated each cell marker’s ability to distinguish PCs from SMCs and other cell types by calculating the sensitivity, specificity, positive predictive values (PPV), negative predictive value (NPV), and accuracy. Of the 66 PCs counted, 34.8% expressed *HIGD1B*, 45.5% expressed *CSPG4*, and 75.8% expressed *PDGFRB*. When comparing the ability of all three markers to distinguish PCs from SMCs, *HIGD1B* had a specificity of 100% (*CSPG4:* 80.2%*, PDGFRB:* 71.7%) and accuracy of 75.0% (*CSPG4:* 66.9%, *PDGFRB:* 73.3%) (Fig. 1D; Fig. EV2B bottom 2 × 2 tables). When calculating the ability of *HIGD1B* to distinguish PCs from all other cell populations, we found that *HIGD1B* had a higher PPV (46.9%) compared to *CSPG4* (28.6%) and *PDGFRB* (21.8%). *HIGD1B* also showed the highest specificity for PCs (97.9%), outperforming *CSPG4* (94.0%) and *PDGFRB* (85.6%). The accuracy of *HIGD1B* in identifying PCs was 94.7%, compared to *CSPG4* (91.5%), and *PDGFRB* (85.1%) (Fig. EV2C). Overall, using *HIGD1B* as a cell marker to identify PCs in human lung tissue had higher specificity, PPV, and accuracy than *PDGFRB* and *CSPG4*.

### *Higd1b-*CreERT2 knockin mouse line is constructed by CRISPR

**Figure 2:**
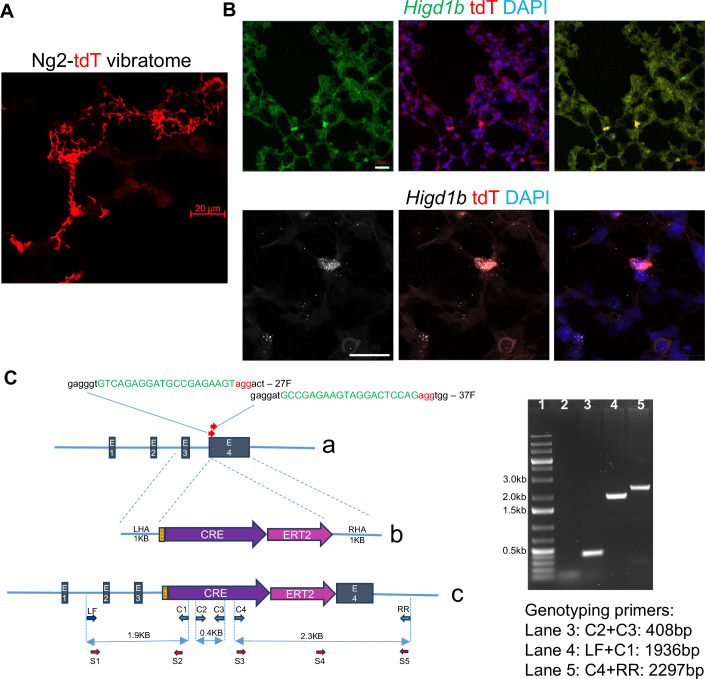
*Higd1b-*CreERT2 knockin mouse line is constructed by CRISPR*.* (**A**) PCLSs were obtained from *Cspg4-CreERTM::Ai14* (Ng2-tdT) mice. Scale bar: 20 µm. (**B**) RNAscope of OCT sections from *Ng2-tdT* lungs show the coexpression of *Higd1b* mRNA (green and white) and the tdT protein expression (red). Nuclei were counterstained with DAPI (blue). Scale bar: 20 µm. (**C**) Schematic figure for Cre-ER insertion and PCR validation of insertion. a: *Higd1b* locus exon 1–4 and location of two gRNA and sequences in green, PAM sites in red and adjacent nucleotides in black after ATG. b: the Knock-in targeting construct: left (LHA) and right homology arms (RHA) flanked by P2A-CRE-ERT2, c. genomic structure after P2A-CRE-ERT2 knock-in. Primer pair LF + C1 identifies 5’ end, and C4 + RR identifies ERT2 and 3’ end, C2 + C3 Cre gene. Left: Two founder mice, #12 and #25, were identified by Cre-specific PCR using C2 + C3 (lane 3) primers LF is located outside of LHA, C1 is located 5’ of Cre, C4 is located at the 3’ of Cre, and RR is outside of RHA. Source data are available online for this figure.

To validate *Higd1b* as a PC-specific cell marker, we designed *Higd1b*-mRNA probes and performed RNAscope on *Cspg4-CreERTM::R26-tdTomato* (Ng2-tdT) lungs. In precision cut lung slices (PCLSs), parenchymal tdT+ cells clearly demonstrated classical PC morphology, including an oval cell body and long punctate processes, partially wrapping capillary ECs (Fig. 2A). We applied *Higd1b* probes on OCT embedded Ng2-tdT lungs and found 100% co-localization of *Higd1b* mRNA with endogenous tdT (Fig. 2B). *Higd1b* positive punctate dots also stained some cells without tdT, suggesting incomplete tamoxifen induction of Ng2-tdT recombination. In larger-sized arteries, no *Higd1b* staining was found in the smooth muscle layers (Appendix Fig. S8). After RNAscope validation, we sought to generate a novel, tamoxifen-inducible, knockin Cre-LoxP mouse line using CRISPR (Fig. 2C). A total of 47 pups were screened by PCR using Cre-specific primers, and two founders were identified, #12 and #25. The genotyping results shown were from mouse #25. To confirm target-specific intact integration of the donor construct in the *Higdb1b* locus, for LHA the forward primer LF is designed in the intronic region between exon-1 and exon-2 and C1 reverse primer in 5’ region of Cre (1.9 kb); the RHA is screened using forward primer C4 located in 3’ of Cre and reverse primer RR is outside of RHA in an intron (2.3 kb) (Fig. 2C). Sanger sequencing of PCR products from left and right homology arms confirmed knock-in of *Higdb1-P2A-CRE-ERT2* construct in exon 4.

### *Higd1b-Cre+* cells specifically label pulmonary and cardiac PCs

**Figure 3:**
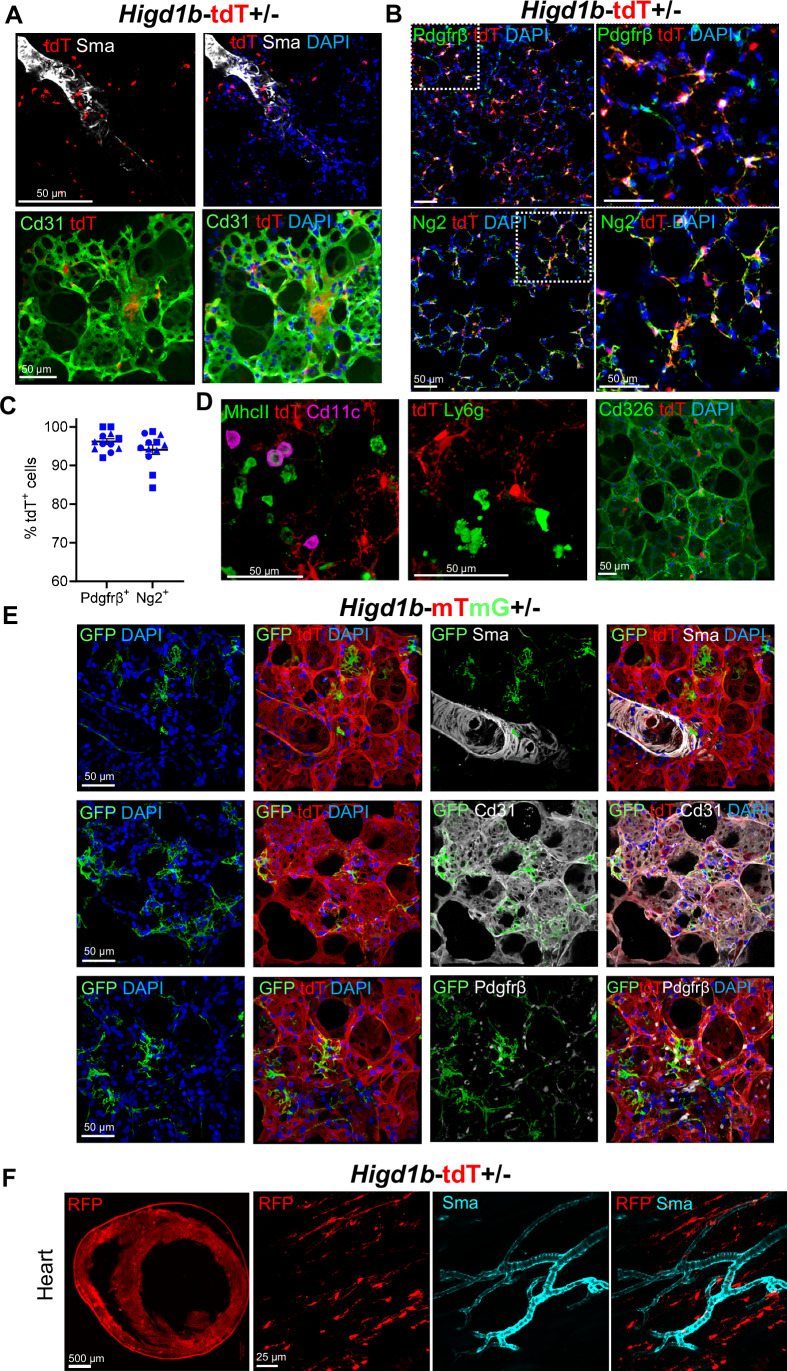
*Higd1b-Cre+* cells specifically label pulmonary and cardiac PCs. (**A**) Representative images of lung tissue from *Higd1b-tdT*+*/−* mice stained for Sma (white), Cd31 (green), and DAPI (blue). PCs were labeled with endogenous tdT reporter (red). Scale bar: 50 µm. (**B**) Representative images of PCLSs from *Higd1b-tdT*+*/−* normoxic mice stained for Pdgfrβ (green, top) and Ng2 (green, bottom). Nuclei were stained with DAPI (Blue). White boxes indicate magnified areas shown in the right panels. Scale bar: 50 μm. (**C**) Quantification of tdT+ PCs from the lungs of *Higd1b-tdT*+*/*– mice expressing Pdgfrβ and Ng2 (*N* = 4 per group). Each symbol represents each lung. Each dot represents an individual image analyzed. (**D**) Representative images of lung tissue from *Higd1b-tdT*+*/−* mice stained for macrophages using MhcII (green) and Cd11c (magenta, left panel), neutrophils with Ly6g (green, middle panel) and epithelial cells with Cd326 (green, right panel). Scale bar: 50 μm. (**E**) Representative images of GFP+ PCs (green) from lung tissue of *Higd1b-mTmG*+*/*– mice stained for Sma (white, top panel), Cd31 (white, middle panel), Pdgfrβ (white, bottom panel), and DAPI (blue). Scale bar: 50 µm. (**F**) A precision cut heart slice from *Higd1b-tdT*+*/−* cleared and stained for RFP and Sma (right panels). Scale bar: 500 µm. The right panels show a magnified area where RFP-positive cells are negative for Sma (cyan). Scale bar: 25 µm. Source data are available online for this figure.

To further validate the specificity of *Higd1b* for labeling PCs, we bred *Higd1b-*CreERT2 with a *R26-tdTomato* (Ai14, *Higd1b-tdT*^*fl/wt*^, referred to as *Higd1b-tdT*+*/*–) and a *R26-mTmG (*referred to as *Higd1b-mTmG*+*/*–) reporter mouse, followed by 0.2 mg/body weight (g) tamoxifen injection. Confocal images of PCLSs from *Higd1b-tdT*+*/*– mice revealed tdT positive (+) cells with morphology representative of pulmonary PCs, including a central, oval body with multiple elongated processes. These cells were predominantly located within pulmonary capillaries, not in distal arterioles (>25 µm). Importantly, there was no co-expression of tdT+ cells with the EC marker Cd31 (green) or SMC marker Sma (white) (Fig. 3A). Additional immunofluorescence (IF) staining of lung tissue for commonly used PC markers Pdgfrβ and Ng2 showed that the majority of tdT+ cells were found to co-express Pdgfrβ (96.2 +*/*– 0.72%) and Ng2 (94.1 +*/*– 1.26%), although staining for both markers was seen in tdT negative (−) cells (Fig. 3B,C). To supplement IF staining, fluorescence-activated cell sorting (FACS) was performed on *Higd1b-tdT*+*/*– lungs and revealed that 96.9% of tdT+ cells expressed Pdgfrβ and 84.4% of cells expressed Ng2 (Appendix Fig. S9). Additional IF staining for markers of macrophages, neutrophils, and epithelial cells showed no coexpression of any marker with tdT+ or GFP+ cells (Fig. 3D; Appendix Fig. S10). In parallel, IF staining and confocal microscopy from *Higd1b-mTmG*+*/*– mice demonstrated similar findings. GFP+ cells with typical PC morphology distributed throughout the parenchyma in direct contact with Cd31+ ECs. The majority of GFP+ cells coexpressed Pdgfrβ but did not express Sma (Fig. 3E). In control experiments, wild-type mice (*WT-tdT*+*/*– and *WT-mTmG*+*/*–) injected with similar doses of tamoxifen as well as knockin mice (*Higd1b-tdT*+*/*– and *Higd1b-mTmG*+*/*–*)* without tamoxifen did not show any endogenous tdT or GFP labeling (Appendix Fig. S11), suggesting the reporter expression is specifically driven by Cre expressing cells after tamoxifen induction.

To test the specificity of *Pdgfrb* and *Cspg4* for PCs, we examined PCLSs from *Higd1b-tdT*+*/*– mice with IF staining for both markers and found expression of Pdgfrβ and Ng2 on tdT+ cells as well as vascular Sma+ SMCs (Appendix Fig. S12A). To further demonstrate the non-specificity of these markers, we generated two mouse models, *Cspg4*-CreERTM (Jax strain #008538)::*R26-mTmG* (Jax strain #007576) and *Pdgfrb*-CreERT2 (Jax strain #029684)::Ai14. After injecting *Cspg4-mTmG* mice with tamoxifen (0.2 mg/g), GFP endogenous expression was seen on both PCs in the capillaries and Sma+ SMCs on pulmonary arteries (Appendix Fig. S12B). In addition, tdT+ cells from *Pdgfrb-tdT* mice were found in large quantities throughout the lung parenchyma and outlining vessel-like structures, consistent with published data of the unreliable nature of Pdgfrβ labeling exclusively for PCs (Alvarez-Aznar et al, 2020; Mayr et al, 2022) (Appendix Fig. S12B). Taken together, this data confirmed that both *Pdgfrb* and *Cspg4* were not specific for pulmonary PCs.

We then evaluated precision-cut heart slices from *Higd1b-tdT*+*/*– because of our prior scRNA-seq results suggesting the expression of *Higd1b* in cardiac PCs (Baek et al, 2022). We observed tdT+ cells stained for red-fluorescent protein (RFP) labeled PCs in capillaries throughout the myocardium, which did not coexpress Sma (Fig. 3F). In addition to the cardiorespiratory system, PCs play critical roles in numerous diseases, such as Alzheimer’s, diabetic retinopathy, and renal fibrosis, across numerous organ systems (Li and Fan, 2023). We therefore examined additional organs harvested from *Higd1b-tdT*+*/*– mice to assess the endogenous labeling of PCs. Similar to heart and lung samples, confocal microscopy of brain, skeletal muscle, pancreas, heart, intestine, and connective tissue surrounding the descending aorta demonstrated Sma-/tdT+ PCs in direct contact with the microvasculature (Fig. EV3A). In the kidney, tdT+ cells were mainly localized to peritubular capillaries in the medulla, with some in close contact with renal arterioles/venules and minimal coverage in the calyx and cortex. Further analysis of renal tissue showed that 4.5% of all cells were endogenously labeled with tdT, and 36% of tdT+ cells coexpressed Sma (Fig. EV3). In addition, the retina exhibited a notable presence of tdT+ labeling of PCs, with 7% tdT+ cells co-expressing Sma at the bifurcation of a vessel (Fig. EV3B). There were no tdT+ cells in the spleen or liver of *Higd1b-tdT*+*/*– mice (Fig. EV3C). Quantification of tdT+ cells across different organ systems was shown in Fig. EV3D–F. Although an in-depth analysis for endogenous tdT-labeling in other organs was not conducted, these preliminary results highlighted the novel *Higd1b-tdT*+*/*– knockin model holds promise for investigating PC biology and function across different organ systems.

**Figure EV3:**

tdT cells from *Higd1b-tdT*+*/−* label PCs in other organs in vivo. (**A**) Stainings of brain, skeletal muscle, pancreas, heart, intestine, and connective tissue around the descending aorta from *Higd1b-tdT*+*/−* mice for Cd31 (green), Sma (white), and DAPI (blue). tdT endogenous reporter labeled PCs are in red. Note the presence of PCs found within multiple organ systems. Scale bar: 50 µm. (**B**) The kidney (top two panels) and retina (bottom panel) from *Higd1b-tdT*+*/−* mice were stained for Cd31 (green), Sma (white), and DAPI (blue). tdT+ PCs are in red. Scale bar: 50 µm. (**C**) Liver (top panel) and spleen (bottom panel) from *Higd1b-tdT*+*/−* mice stained for Cd31 (green), Sma (white), and DAPI (nuclei: blue) reveal an absence of tdT+ PCs (red) in both organs. Scale bar: 50 µm. (**D**) Quantification of tdT+ cells in various organs from *Higd1b-tdT*+*/−* mice in each image field inspected. Image area inspected = 429.5 μm^2^. *N* = 3 for all organs except heart tissues (*N* = 2). Each dot represents an individual image analyzed. Data presented as mean ± SEM. (**E**) tdT+ cells from the kidney taken from *Higd1b-tdT*+*/−* mice (left) alongside binary immunofluorescence (middle). Scale bar: 50 µm. Quantification of tdT+ cells from the whole cell population identified with DAPI staining is displayed. *N* = 2, with each dot represents an image taken by an individual animal. (**F**) Percentage of tdT+, Sma+ cells per image field from the retina and kidney obtained from *Higd1b-tdT*+*/−* mice. Inspected image area = 429.5 μm^2^. *N* = 3 for each group, with each dot representing an individual image analyzed. Data presented as mean ± SEM.

### Lineage tracing study reveals that *Higd1b-tdT+* cells originating from the capillaries accumulate in muscularized distal arterioles over various durations of hypoxic exposure

Utilizing Ng2-tdT mice, our group previously showed that Ng2+ mural cells accumulated in the microvasculature and contributed to the development of Hx-induced PH (Yuan et al, 2020). However, since Ng2 is a shared cell marker expressed in multiple mural cell populations (Appendix Fig. S12), particularly SMCs, it is impossible to definitively conclude that PCs directly contribute to arteriole muscularization and vascular remodeling. To address this limitation, we conducted lineage-tracing studies using *Higd1b-tdT*+*/*– mice, allowing for a more precise description of the contribution of PCs to vascular remodeling across different time points during Hx exposure.

**Figure 4:**
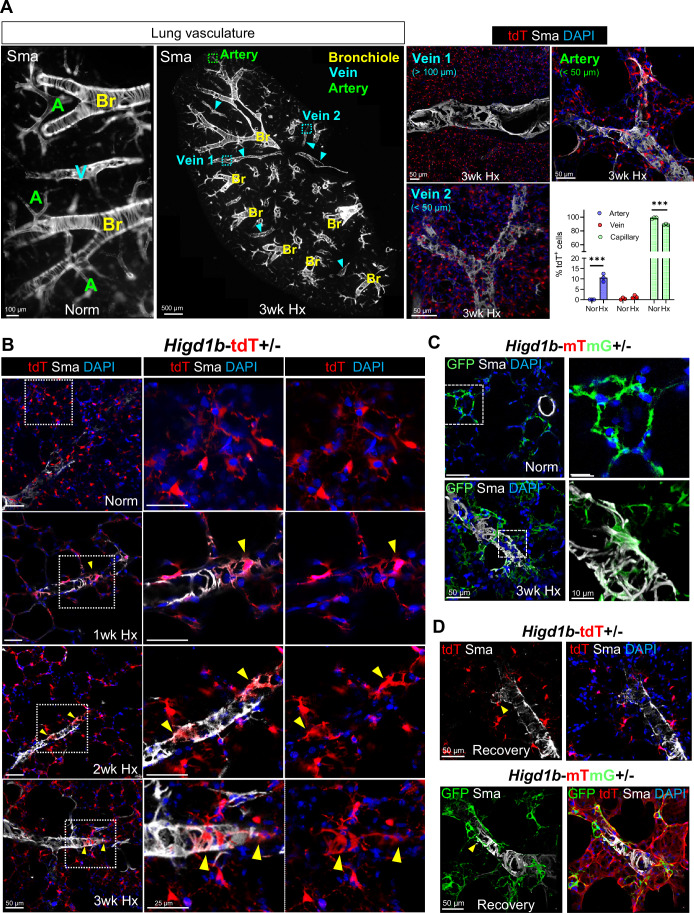
Lineage tracing reveals that *Higd1b-Cre+* cells originating from the capillaries accumulate in muscularized distal arterioles over various durations of hypoxic exposure. (**A**) Left: Images of PCLSs from *Higd1b-tdT*+*/*– mice in normoxia show the distribution and pattern of Sma (white) in bronchioles (Br, yellow), arteries (A, green), and veins (V, cyan). Scale bar: 100 μm. Middle: PCLSs from 3 wk Hx *Higd1b-tdT*+*/*– mice display Sma staining patterns in bronchioles (yellow), arteries (green), and veins (cyan). Blue arrowheads indicate the location of veins. Scale bar: 500 μm. Right: Represenative PCLSs showing tdT+ PCs (red) surrounding veins and arteries in *Higd1b-tdT*+*/*– mice after 3 wks of Hx. DAPI: blue. Scale bar: 50 μm. The bottom right shows the statistical analysis of tdT+ PC locations in the arteries (*P* = 0.0007), veins (*P* = 0.156), and capillaries (*P* = 0.0002) of *Higd1b-tdT*+*/*– mice in normoxia (*N* = 3) and 3 wks of Hx (*N* = 3). Each dot represents an individual image analyzed. Data is presented as mean ± SD with statistical analysis performed with an unpaired t-test. ***P < 0.001; indicates statistical significance. (**B**) PCLSs from *Higd1b-tdT*+*/*– mice exposed to 1 wk, 2 wk, and 3 wks of Hx show Sma (white) and DAPI (blue) staining. tdT+ PCs (red) accumulated in the distal vasculature after 1 wk of Hx and increased their coverage on arterioles after prolonged exposure to Hx. Yellow arrowheads indicate tdT+ PCs. Magnified areas are shown in adjacent panels. Scale bar: 25 µm. (**C**) PCLSs from *Higd1b-mTmG*+*/*– mice in normoxia and after exposure to 3 wks of Hx. GFP+ PCs on distal arterioles post-Hx. Sma: white. DAPI: blue. White boxes highlight magnified images in adjacent panels. Scale bar: 50 µm. (**D**) Lineage tracing of the recovery model in *Higd1b-tdT*+*/*– (top) and *Higd1b-mTmG*+*/*– (bottom) mice exposed to 3 wks of Hx followed by 3 wks of normoxia shows that the majority of reporter cells returned to the parenchyma with only a small number of PCs on the distal arterioles (yellow arrowheads). PCLSs were stained for Sma (white), and DAPI (blue). Scale bar: 50 µm. Source data are available online for this figure.

Before performing fate-mapping experiments, we inspected the bronchioles and different vessels in the pulmonary vascular tree (capillaries, arteries, and veins) to identify the location of tdT+ PCs in *Higd1b-tdT*+*/*– mice in normoxia and after exposure to 3 wks of Hx (Fig. 4A). To distinguish between arterioles (A) and venules (V), we examined the spatial distribution of Sma on vessels larger than 50 µm in diameter. Arteries and arterioles were characterized by intense, continuous Sma expression and their adjacency to bronchioles (Br), while veins exhibited an inconsistent and patchy Sma pattern and were located between bronchioles in parenchymal regions (Kretschmer et al, 2013). Sma also stained bronchiole SMCs in a rubber band pattern. Under normoxic conditions, nearly all PCs (99.6%) were confined to the capillaries, with almost none in the arteries or veins. In *Higd1b-tdT*+*/*– mice exposed to 3 wks of Hx, PCs remained predominantly within the capillary networks (89.1%). However, some tdT+ cells accumulated in arteries compared to normoxic samples (11% vs. 0.0%, *P* < 0.0001) (Fig. 4A, bottom right). To determine if the accumulation of PCs on the distal arterioles in response to Hx resulted from proliferation, we performed Ki67 staining on PCLSs from 3 wk Hx and normoxic *Higd1b-tdT*+*/*– and quantified the number of Ki67+/tdT+ cells. There was minimal to no Ki67 expression in both normoxic and Hx pericytes (0.0 +*/−* 0% vs. 0.84 +*/*– 0.47%, *P* < 0.2), suggesting that PC migration, rather than proliferation, was responsible for their accumulation on distal arterioles in response to Hx (Appendix Fig. S13).

**Figure EV4:**
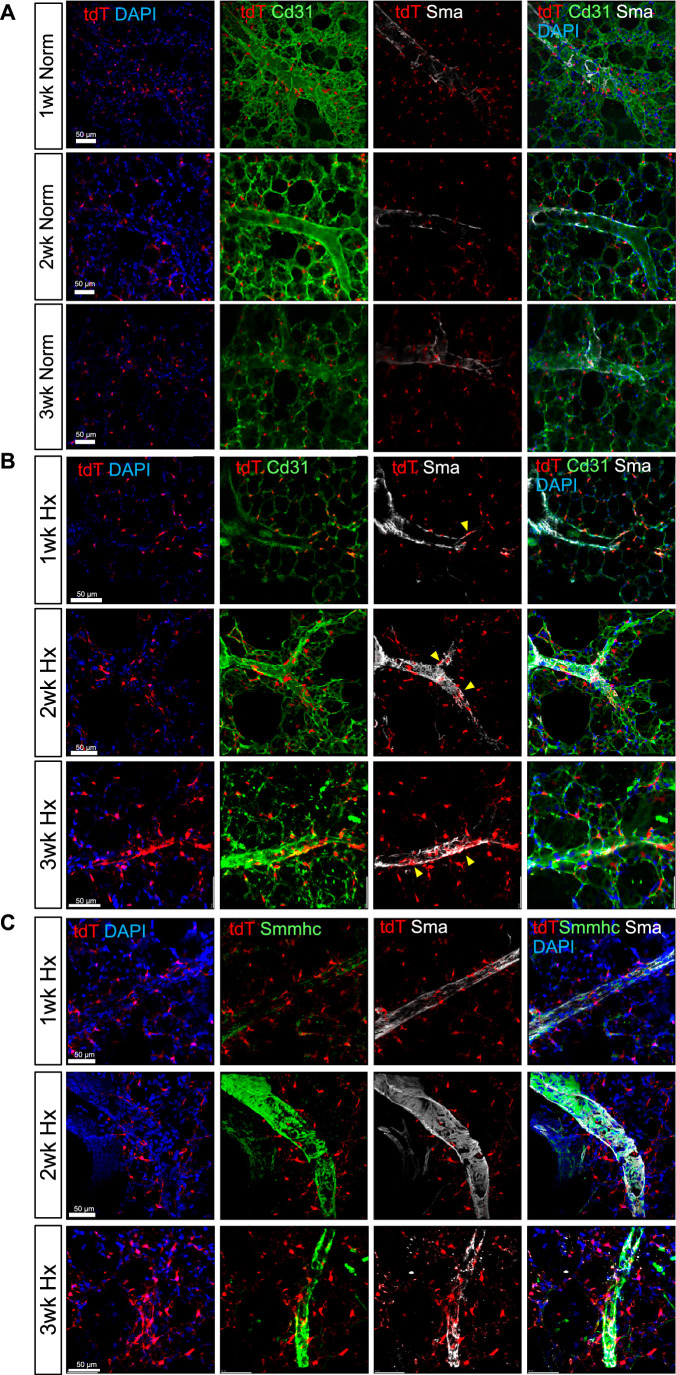
Lineage tracing shows that tdT*+* cells accumulate in muscularized distal arterioles by different hypoxic exposure times. (**A**) tdT+ PCs from *Higd1b-tdT*+*/−* mice showing Sma negative tdT+ PCs(red) located in the parenchymal region and distal arterioles after 1, 2, and 3 wks of normoxia. Cd31 in green and Sma in white and DAPI in blue. (**B**) Representative images of PCLSs from *Higd1b-tdT*+*/−* mice show tdT+ PCs (red) on distal arterioles (Cd31, green) coexpressing Sma (white) after exposure to 1, 2, and 3 wks of Hx. Yellow arrowheads highlight the accumulation of PCs on remodeled distal arterioles that coexpress Sma after Hx exposure. Scale bar: 50 μm. (**C**) Accumulation of tdT+ PCs (red) in muscularized distal arterioles, with staining for SMC markers Smmhc (green) and Sma (white) after 1, 2, and 3 wks of Hx. DAPI: blue. Scale bar: 50 µm. Source data are available online for this figure.

After demonstrating tdT+ migration and transition in response to 3 wk Hx, we exposed mice to various durations of Hx (1 wk, 2 wks, 3 wks) and harvested lung tissues for evaluation (Klouda et al, 2020; Klouda et al, 2021). Using confocal microscopy and IF staining, we examined PC morphology, distribution, and expression of SMC cell markers (Sma or Smmhc). After 1 wk of Hx, endogenously labeled tdT+ cells were found in close proximity to the distal arterioles (<50 µm), with a small subset of tdT+ cells being found directly in muscularized vessels co-expressing Sma. Compared to normoxic samples, PCs in the distal arterioles demonstrated a spindle-shaped morphology, closely resembling SMCs with reduced process length compared to normoxic PCs. The majority of PCs, however, were still localized within the lung parenchyma and maintained direct contact with capillary ECs (Fig. 4B). Prolonged exposure to Hx for 3 wks resulted in an increased number of tdT+ cells with spindle-shaped morphology and shorter processes, coexpressing Sma in remodeled arterioles. This finding demonstrated the transition of PCs to SMC-like cells and their direct contact with muscularized vessels. Quantification of Sma in tdT+ PCs from *Higd1b-tdT*+*/*– exposed to 3 wk Hx revealed 10.6% of PCs coexpressing Sma, compared to 0.0% of the normoxic tdT+ cells (Appendix Fig. S14). Despite this transition, many PCs remained within the lung parenchyma and maintained direct contact with capillary ECs. Additional IF staining for Cd31, Sma, and Smmhc confirmed that tdT+ cells did not express Cd31 but coexpressed mature SMC marker Sma and Smmhc after 1 wk, 2 wks, and 3 wks of Hx exposure (Fig. EV4C).

Next, we performed similar lineage-tracing experiments in *Higd1b-mTmG*+*/*– mice to validate findings from *Higd1b-tdT*+*/*– mice. Under normoxic conditions, no visualized GFP+ cells exhibited coexpression for Sma or were located on arterioles (Fig. 4C, top panel). However, after 3 wks of Hx, GFP+ cells accumulated in the distal arterioles (25–50 µm), co-expressing Sma and exhibited a spindle-like morphology (Fig. 4C, bottom panel).

The chronic Hx mouse model is a well-established experimental PH model which demonstrates the reversal of hemodynamics and pulmonary vascular alterations after 3 wks of normoxia (referred to as the recovery model) (Bonnet et al, 2002). Upon returning Hx, *Higd1b-tdT*+*/*– and *Higd1b-mTmG*+*/*– mice to normoxic conditions for 3 wks, we observed a loss of PC accumulation on the distal arterioles and the restoration of “normal” PC morphology seen at baseline, suggesting a dynamic movement of PCs in response to oxygen levels (Fig. 4D). However, we noticed that a small portion of tdT+ or GFP+ cells in recovery mice still wrapped and resided in the distal arterioles but no longer expressed Sma. We speculate that longer exposure to normoxia may result in a complete return of PC to their parenchymal location.

### Two subtypes of PCs are identified using human lung scRNA-seq

A key finding in our fate-mapping experiments was the distinct location and morphological changes of *Higd1b* + PCs after exposure to Hx. Since tdT+ cells accumulated in remodeled arterioles or remained in the capillaries after exposure to Hx, we speculated that there may be different PC subtypes within the pulmonary capillaries. These subsets of PCs may have different roles in disease development and be distinguished from one another using known PC markers *HIGD1B/Higb1b, PDGFRB/Pdgfrb*, and *CSPG4/Cspg4*. Therefore, we sought to determine if such PC subsets could be identified, providing further insight into the role of PCs in the development of Hx-induced vascular remodeling using scRNA-seq.

**Figure 5:**
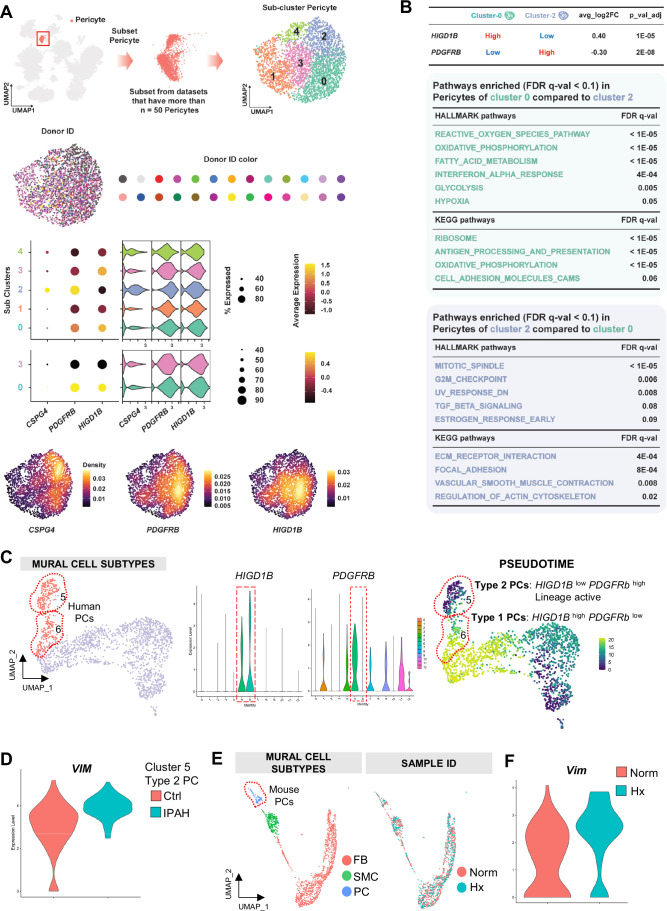
Two subtypes of PCs are identified using human lung scRNA-seq. (**A**) UMAP visualization of five PC sub-populations within the annotated PC cluster from the ‘Human Lung Cell Atlas (core)’ which includes 50 or more annotated cell types. Utilizing Harmony and selecting data from studies with more than 400 PCs, we narrowed the PC sub-populations down to 2992 PCs to minimize the batch effects. In the middle, Donor distribution across PC sub-populations is shown. Data processing steps and the distribution of other potential confounders across the PC sub-populations are provided in Appendix Fig. S15. Dot, Violin and Density plots in the bottom panels illustrate the relative expression of PC markers (*CSPG4, PDGFRB*, and *HIGD1B*) across PC subclusters, highlighting the heterogeneity among these sub-clusters. Subcluster 0 and 3 were directly compared to identify the subcluster with the highest *HIGD1B* expression. (**B**) Differential expression (DE) analysis utilizing the Wilcoxon rank-sum test was conducted between subcluster 0 (highest *HIGD1B* expression among the subclusters) and subcluster 2 (lowest *HIGD1B* expression among the subclusters). The table above shows significantly higher *HIGD1B* expression and significantly lower *PDGFRB* expression in subcluster 0 compared to subcluster 2. Gene Set Enrichment Analysis (GSEA) comparing PC sub-Clusters 0 and 2 shows that sub-Cluster 0, marked by significantly higher expression of *HIGD1B*, is enriched in pathways related to metabolic activity and stress response. In contrast, sub-cluster 2, with a higher expression of *PDGFRB*, is associated with pathways involved in focal adhesion, cell cycle regulation, and movement. A comprehensive list of enriched pathways with an FDR below 0.1 can be found in Appendix Fig. S16. (**C**) Human IPAH and control mural and stromal cell subtypes were re-clustered from the previously published data. Sub-clusters 5 and 6 were identified as PC clusters enriched with *HIGD1B* expression. The Violin plot shows higher *HIGD1B* expression in sub-Cluster 6 compared to sub-Cluster 5, while *PDGFRB* is highly expressed in sub-Cluster 5. Pseudotime analysis reveals that Type 1 PCs (sub-Cluster 6, *HIGD1B*^*high*^*PDGFRB*^*low*^) are predominantly late lineages (yellow) whereas Type 2 PCs (sub-Cluster 5, *HIGD1B*^*low*^*PDGFRB*^*high*^) display a mix of early (purple) and middle (green) lineages. (**D**) The Violin plot shows *VIMENTIN* (*VIM*) expression is increased in IPAH Type 2 PCs from IPAH patients compared to healthy donor Type 2 PCs in sub-Cluster 5. (**E**) A re-analyzed UMAP plot of mural cells from murine lungs exposed to Hx vs. normoxia identifies PC cluster outlined by a dashed area. The UMAP of sample ID is also included. (**F**) The Volin plot demonstrates that *Vimentin* (*Vim*) expression is elevated in PCs from Hx-mice compared to normoxic PCs.

To explore the heterogeneity within PC sub-populations in relation to *HIGD1B* expression, we first subset the annotated PC cluster from the “Human Lung Cell Atlas” (Travaglini et al, 2020). Utilizing Harmony (Korsunsky et al, 2019) and selecting data from studies with more than 400 PCs, we narrowed the data down to 2992 PCs. This allowed us to minimize the batch effect from sub-clustering, leading to the identification of five distinct PC sub-clusters (Fig. 5A; Appendix Fig. S15). Notably, sub-Cluster 0 and 3 exhibited high expression levels of *HIGD1B*. When directly comparing sub-Cluster 0 and 3, sub-Cluster 0 had the highest *HIGD1B* expression. Sub-cluster 2 displayed the lowest expression of *HIGD1B*. Particularly, despite its lower *HIGD1B* expression, sub-cluster 2 demonstrated a higher expression of *CSPG4* and *PDGFRB*.

To further dissect the molecular difference between PCs with high and low *HIGD1B* expression, we performed DE analysis between sub-clusters 0 and 2, utilizing the Wilcoxon rank-sum test. Subsequently, we conducted a pre-ranked Gene Set Enrichment Analysis (GSEA) to identify key pathways and genes distinguishing these sub-populations (Fig. 5B; Appendix Fig. S16). Our analysis revealed that the relative expression level of *HIGD1B* was significantly higher in sub-Cluster 0, whereas the relative expression level of *PDGFRB* was significantly higher in sub-Cluster 2. The GSEA underscored functional disparities between the two sub-populations. In sub-Cluster 0, pathways associated with metabolic activity, such as oxidative phosphorylation and glycolysis, were prominent, suggesting a metabolically active phenotype. In addition, this sub-cluster showed enrichment in the reactive oxygen species pathway, Interferon-α/β response, highlighting potential roles in cellular stress response and inflammation. Intriguingly, sub-Cluster 2 displayed enrichment in pathways related to cell cycle and development, such as mitotic spindle and G2M checkpoint, which may reflect a migration-associated state. The presence of pathways like estrogen response early and extrea cellular matrix (ECM) receptor interaction indicates a potential involvement in tissue remodeling and hormonal responses. KEGG pathways, including focal adhesion, ECM receptor interaction, vascular smooth muscle contraction, and regulation of actin cytoskeleton, suggested cellular motility, contractility, and lineage transition. These divergent pathways between sub-Cluster 0 and 2 suggest a different functional state of PCs, with sub-Cluster 0 displaying PC classic characteristics of metabolic and structure maintenance within the lung tissue, while sub-Cluster 2 aligns with stress response engagement, including cellular proliferation, movement, and lineage transition. A full list of enriched pathways identified in PCs from sub-Cluster 0 and sub-Cluster 2 can be seen in Appendix Fig. S16.

Next, we re-analyzed IPAH PCs and control PCs utilizing previously published scRNA-seq data (Kim et al, 2024; Saygin et al, 2020). Among the sub-re-clustered 13 mural cell clusters, only sub-Clusters 5 and 6 had expressions of *HIGD1B* (Fig. 5C left; Appendix Fig. S17). Violin plots show that sub-Cluster 6 is *HIGD1B*^*high*^*PDGFRB*^*low*^, defined as Type 1 PCs whereas sub-Cluster 5 is *HIGD1B*^*low*^*PDGFRB*^*high*^, defined as Type 2 PCs. Pseudotime analysis  suggests that Type 2 PCs are lineage active by purple/green color, whereas Type 1 PCs are quiescent and have mature lineage status by yellow using color-coded scale (Fig. 5C, right). When referring back to the pathways analyzed in sub-types of PCs in Fig. 5B, we speculated that Type 1 PCs aligned with sub-Cluster 0, which was characterized by reactive oxygen species (ROS) signaling, metabolism, and Hx pathways. These cells, embedded within the capillary network, were highly relevant to O_2_-CO_2_ gas exchange compared to Type 2 PCs. Their high *Higd1b* expression suggested sensitivity to oxygen levels, which could explain the activation of ROS-related pathways. In contrast, Type 2 PCs corresponded to sub-Cluster 2, adjacent to arterioles. Pathways enriched in this sub-cluster involved cell cycle regulation, focal adhesion, and contraction, reflecting their more active lineage state and the expression of migration-related genes.

Differentially expressed genes (DEG) comparing IPAH Type 2 PCs with healthy Type 2 PCs only revealed two significantly altered genes (adjusted *P* < 0.02), which are *VIM* (Fig. 5D) and *ITM2C*. IPAH Type 1 PCs compared to healthy Type 1 PCs also only revealed two significantly altered genes (adjusted *P* < 0.02), which are *MT2A* and *MT1M*. When re-analyzing Hx murine mural cells scRNA-seq data from our recently published work(Kim et al, 2024), we identified PC clusters from other mural populations using the expression level of *Higd1b* (Fig. 5E). Due to limited cell numbers, we can not further sub-cluster the murine PC population. By DEG analysis, *Vim* was 3.4-fold upregulated in hypoxic PCs compared to normoxic PCs (Fig. 5F).

### Type 2 PCs in *Higd1b-tdT*+*/*– lungs show upregulated Vimentin expression after exposure to Hx

**Figure 6:**
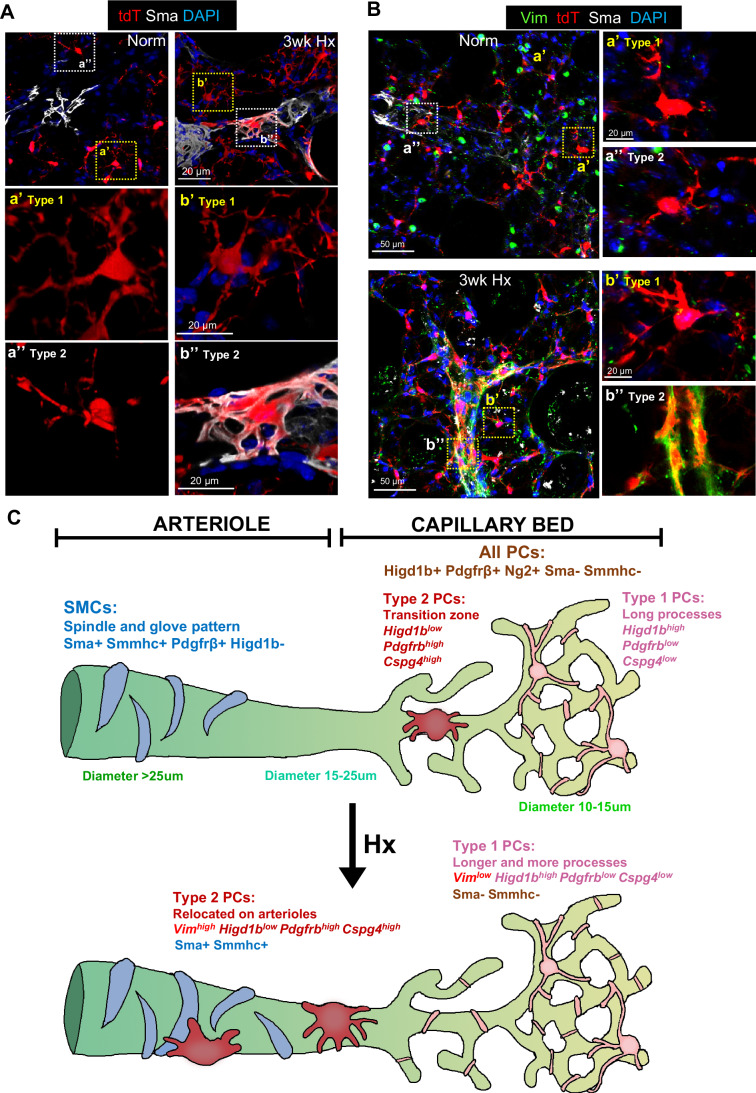
Type 2 PCs in *Higd1b-tdT*+*/*− lungs show upregulated Vimentin expression after exposure to Hx. (**A**) High-resolution images show PCLSs obtained from *Higd1b-tdT*+*/*− lungs exposed to 3 wks of Hx stained for Sma (white) and DAPI (blue), highlighting the morphological differences between Type 1 and Type 2 PCs. Yellow boxes indicate Type 1 PCs, while white boxes highlight Type 2 PCs within the microvasculature. Panel a’ represents a Type 1 PC in normoxia, characterized by long and thin processes located in the capillaries, while panel a” shows a Type 2 PC near a partial Sma+ arteriole in normoxia. Panel b’ shows a Type 1 PC located in the capillaries in Hx with longer and thicker processes compared to normoxic conditions, while Panel b” shows a Type 2 PC in Hx accumulated and enwrapped around a distal arteriole, coexpressing Sma. Scale bar: 20 µm. (**B**) PCLSs from *Higd1b-tdT*+*/*− mice in normoxia (top panel) and after exposure to 3 wks of Hx (bottom panel) stained for Vim (green), Sma (white), and DAPI (blue). Scale bar: 50 µm. Magnified areas in boxes show Panel a’, depicting a Type 1 PC under normoxia and Panel a” depicting a Type 2 PC in normoxic conditions. Panel b’ highlights a Type 1 PC in Hx without coexpression of Vim, while Panel b” shows a Type 2 PC coexpressing Vim (green) and Sma (white) on an arteriole. Scale bar: 20 µm. (**C**) The proposed model illustrates the location of PC subtypes and arteriolar SMCs in the pulmonary vasculature under physiological conditions and in the development of Hx induced PH. After Hx, Type 2 PCs upregulate Vim, exhibit increased motility and lineage activity. Italic texts represent gene expression, while non-Italic texts denote protein expression. Source data are available online for this figure.

We then performed confocal microscopy and IF staining on normoxic and Hx *Higd1b-tdT*+*/*– mice to determine if Vimentin would be upregulated in Type 2 PCs. Under normoxic conditions, Type 1 PCs were in the parenchymal capillaries (Fig. 6A, Panel a’), whereas Type 2 PCs (Fig. 6A, Panel a”) were close to a Sma+ arteriole. However, after exposure to 3 wks of Hx, Type 1 PCs showed an increased number of processes (Fig. 6A, Panel b’) compared to normoxic Type 1 PCs (Fig. 6A, Panel a’). Hx Type 2 PCs wrapped around muscularized distal arterioles (<50 µm), demonstrated distinct morphology with spindle/flat shape and shorter processes (Fig. 6A, Panel b”) and coexpressed Sma compared to normoxic Type 2 PCs (Fig. 6A, Panel a”, an oval body with thin processes).

**Figure EV5:**
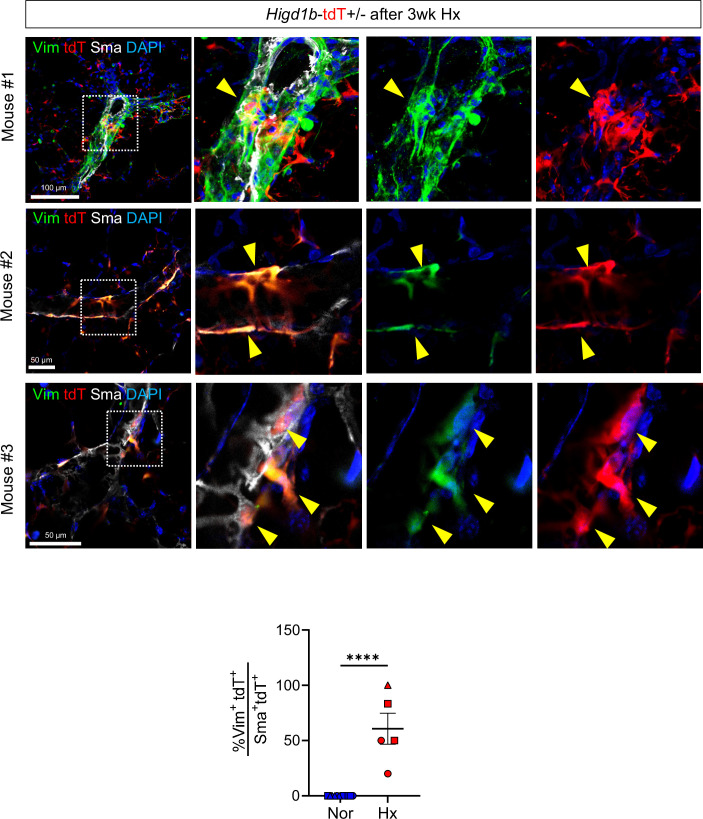
Chronic Hx results in upregulation of Vim in Type 2 PCs accumulated on distal arterioles. PCLSs from three *Higd1b-tdT*+*/−* mice demonstrate the upregulation of Vim (green) in Type 2 PCs (red) accumulated on distal arterioles. Yellow arrowheads highlight the morphological changes of Type 2 PCs and the coexpression of Vim. Sma (white). DAPI (blue). Scale bar: 50 μm. The bottom figure shows the quantification of tdT+ cells coexpressing Vimentin in normoxic and Hx *Higd1b-tdT*+*/−* mice. *N* = 3 for each group. Each dot represents an individual image analyzed. Data is presented as mean ± SEM. Statistical analysis is performed with an unpaired t-test. ****P *< 0.0001* indicates statistical significance. Source data are available online for this figure.

In normoxia, Vimentin (Vim) was seen scattered throughout the parenchyma. Under increased magnification, there was no expression of Vim in Type 1 or Type 2 PCs (Fig. 6B, Panel a’ and a”). After 3 wks of Hx, Vim was upregulated in the outer layer of distal arterioles and in Type 2 PCs (Fig. 6B, panel b”). These staining results were consistent with the Volin plot results in Fig. 5D. After exposure to 3 wks of Hx, roughly 54% of Sma+, tdT+ cells were also Vim+ (Fig. EV5). Taken together, scRNA-seq analysis and experiments in PC lineage tracing suggested that in response to Hx, a subset of PCs (Type 2) relocated in the distal arterioles and expressed higher levels of Vim and lower levels of typical PCs genes, including *Higd1b, Cspg4*, and *Pdgfrb*. This was in contrast to Type 1 PCs, which under physiological conditions, had a low relative expression of Vim and higher expressions of *Higd1b, Cspg4*, and *Pdgfrb* (Fig. 6C). These findings suggest, for the first time, a heterogeneity of PCs in the capillary networks and those treatments targeting all mural cells may affect a wide range of PCs/SMCs and lead to disruption of the microvasculature homeostasis and structure support rather than reversing the vascular remodeling seen in PH. Instead, therapeutic approaches should be considered to exclusively target Type 2 PCs and not affect Type 1 PCs.

### Overexpression of VIM promotes adhesion, migration, and SM22 expression in healthy human lung PCs after exposure to Hx

**Figure 7:**
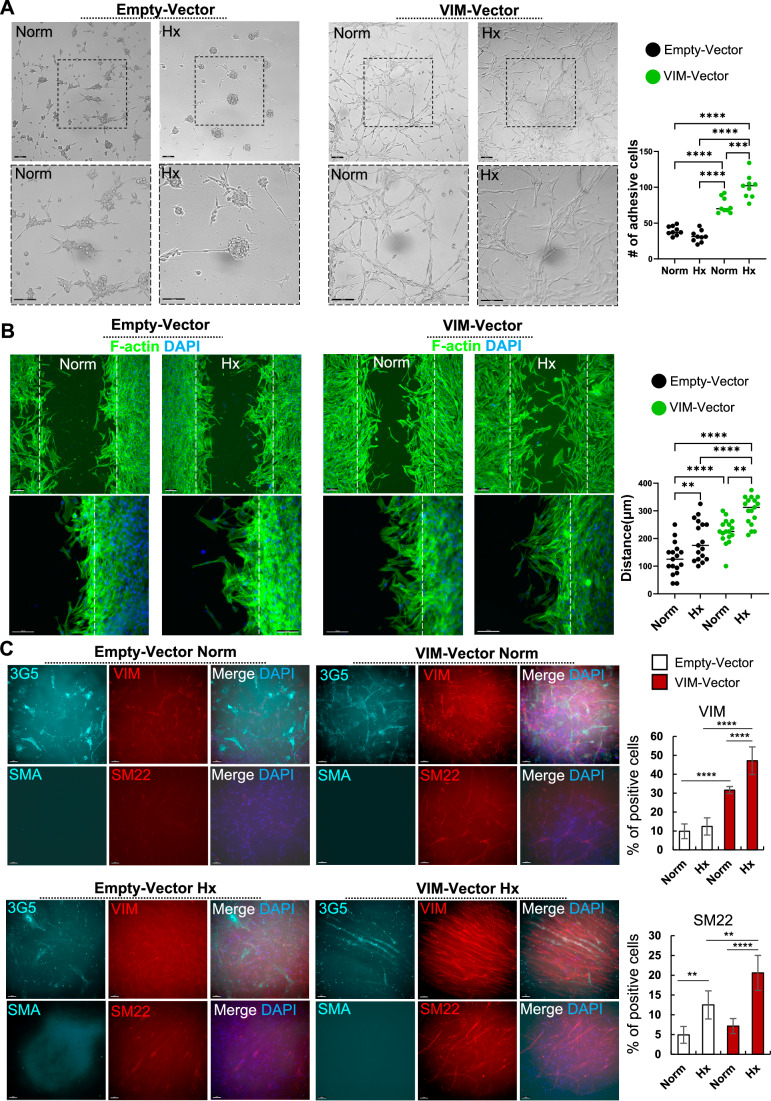
Overexpression of VIM promotes adhesion, migration, and SM22 expression in healthy human lung PCs after exposure to Hx. (**A**) Cell adhesive assays performed on healthy human lung PCs under normoxic and Hx conditions, with and without overexpression of VIM, demonstrate increased PC adhesion in response to Vim. Black boxes highlight areas of increased magnification, showing close contact between VIM overexpression PCs and neighboring cells. Scale bar: 100 µm. The graph on the right quantifies the adhesion of PCs treated with VIM (green) compared to empty vector controls (black). Each dot represents data from three biological replicates, repeated three times. Data is presented as mean ± SEM with statistical analysis performed with one-way ANOVA. ****P < 0.0001 indicates statistical significance. (**B**) Migration assay indicates that VIM overexpression promotes cell migration of healthy PCs. After six hours, PCs treated with VIM plasmid demonstrated increased cell migration, visualized by F-actin (green) and DAPI (blue). The graph on the right quantifies the migration distance of PCs in normoxic and Hx conditions. Each dot represents an average distance of all PCs in a 100 µm × 200 µm area, using three biological replicates (*N* = 3 for each group). Data presented as mean ± SEM with statistical analysis performed with one-way ANOVA. **P < 0.01, ****P < 0.0001 indicates statistical significance. (**C**) Cell culture IF stainings in healthy human lung PCs with and without VIM overexpression for expression of SMC marker (SM22, SMA) in PCs (3G5), along with VIM (red). DAPI (blue). Graphs on the right display the percent of cells acquired from six images coexpressing VIM (top) and SM22 (bottom). Each experiment was repeated three times with three biological replicates. Data is presented as mean ± SEM with statistical analysis performed using one-way ANOVA. **P < 0.01, ****P < 0.0001 indicates statistical significance. Source data are available online for this figure.

To investigate the role and function of VIM in pulmonary PCs, particularly Type 2 PCs in the context of PAH pathology, we upregulated VIM expression in PCs and conducted several biological assays to measure adhesion, migration, proliferation using MTS, wound healing, and IF cell markers under normoxia and Hx (FiO_2_:2.5%). Three healthy PCs isolated from failed donor tissues were transiently transfected with VIM-overexpressing plasmids (Addgene, Plasmid #56439) or pcDNA3.1-empty vectors. GFP was expressed 48 hours (h) post-transfection, confirming an effective transfection (Appendix Fig. S18A). PCs overexpressing VIM had nearly a two-fold increase in adhesion under normoxic conditions (Norm: 39.3 +*/*− 2.12 vs Norm/VIM: 74.4 +*/*− 3.62 cells, *P* < 0.0001) and a three-fold increase in Hx conditions (Hx: 31.3 +*/*− 2.72 vs Hx/VIM: 100.9 +*/*− 5.54 cells, *P* < 0.0001) (Fig. 7A). Using a wound-healing assay, we assessed PC migration under different conditions. After 6 h, control PCs under Hx conditions demonstrated significantly increased migration compared to normoxic PCs (Norm: 126.0 +*/*− 14.12 vs Hx: 193.0 + /17.41 µm, *P* < 0.01). However, VIM overexpression further increased migration distance in both normoxic (Norm: 225.4 +*/*− 11.16 µm, *P* < 0.0001) and Hx conditions (300.7 +*/*− 12.24 µm, *P* < 0.0001) (Fig. 7B). No significant change in proliferation was found in VIM-overexpressing PCs under normoxia or hypoxia conditions (Appendix Fig. S18B), which was consistent with negative Ki67 staining in Hx tdT+ PCs (Appendix Fig. S13). To investigate if PCs transition into SMC-like cells in response to VIM overexpression, we performed IF staining for PC markers (3G5, PDGFRβ) and SMC markers (SM22, SMMHC, SMA) under normoxic and Hx conditions. The percentage of VIM+ cells was significantly increased in VIM transfected cells in normoxic (31.62 +*/*− 1.89 vs 9.82 +/− 3.89%, *P* < 0.0001) and Hx conditions (47.14 +*/*− 7.32 vs 12.38 +*/*− 4.53%, *P* < 0.0001). In addition, VIM overexpression under Hx led to an increased percentage of SM22+ cells (20.59 +*/*− 4.43 vs. 7.14 +*/*− 1.89%, *P* < 0.0001) compared to VIM overexpression under normoxia. The combination of VIM upregulation and Hx resulted in a 1.7-fold increase of SM22+ cells compared to Hx alone (20.59 +*/*− 4.43 vs 12.5 +*/*− 3.55%, *P* < 0.01) (Fig. 7C). However, no expression of SMA (Fig. 7C) was detected under any conditions. Taken together, in vitro experiments suggested that VIM overexpressing caused significant changes in PC biology and function, promoting increased adhesion, migration, and potential transition into SMC-like in both normoxic and Hx conditions.

## Discussion

In this study, we identified *HIGD1B*/*Higd1b* as a unique and specific PC marker in the cardiopulmonary system using scRNA-seq, spatial transcriptomics, and RNAscope for humans and mice (Fig. 1; Appendix Figs. S1–8). Utilizing the Cre-LoxP and CRISPR technologies, we generated a novel, tamoxifen-inducible knockin mouse line (*Higd1b-*CreERT2) which effectively labeled PCs in abundance in the lung, heart (Figs. 2 and 3) and other organs (Fig. EV3). Through lineage tracing studies and scRNA-seq analysis, we demonstrated two subtypes of PCs within the pulmonary capillary networks capable of dynamic transition (Figs. 5 and 6). Type 1 PCs located within the capillary network (diameter <10 µm) maintain homeostasis and integrity and were quiescent. Type 2 PCs located in the zone between capillary and arterioles (diameter <25 µm) were lineage active and had multipotent properties, including the plasticity to transition into SMC-like cells, relocating to the arterioles in response to Hx, contributing to vascular remodeling and the development of Hx-induced PH (Fig. 6).

PCs are an essential component of microvasculature and play key roles in regulating capillary homeostasis, angiogenesis, immune cell recruitment, vascular remodeling, and maintenance of the blood-brain barrier(Crisan et al, 2008; Hartmann et al, 2022; Proebstl et al, 2012; Yuan et al, 2021). They have been implicated in the development of numerous disease processes throughout the body, including PAH, Alzheimer’s disease, and diabetic retinopathy. A significant limitation and challenge in studying PCs is the lack of a clear and specific cell marker to distinguish them from other mural cell populations. Genetic mouse models, combining cell markers, fluorescent reporters, and lineage-tracing lines, represent powerful tools for genetically labeling PCs and tracking their behavior during development and in pathological conditions. Commonly used promoters for PC labeling include *Pdgfrb* (Cuervo et al, 2017*;* Jung et al, 2018)*, Cspg4* (Volz et al, 2015b*;* Yuan et al, 2020*;* Zhu et al, 2008), *Tbx18* (Zhu et al, 2008), *LepR* (Butiaeva et al, 2021), Alkaline Phosphatase AP (Butiaeva et al, 2021), *Myh11* (Hess et al, 2019), *Foxj1* (Rock et al, 2011*)*, and *Foxd1* (Hung et al, 2017) and even dual reporter systems by *Pdgfrb-Flp* and *Cspg4-FSF-CreER* (Nikolakopoulou et al, 2019) or *PdgfraDreER* negative *PdgfrbCreER* positive (Han et al, 2021). However, it is essential to emphasize that none of these markers are exclusive to PCs, as SMCs, FBs, myofibroblasts, and other non-mural cells share these markers with PCs. Consequently, PC lineage-tracing studies cannot definitively rule out potential contamination from other cell types. scRNA-seq is an emerging and powerful tool for analyzing the genetic signature of individual cells, enabling the identification of genes for future diagnostic and therapeutic strategies. Through data mining from publicly available sources, we have identified *HIGD1B/Higd1b* as a unique and specific PC gene. Combining scRNA-seq with corresponding spatial transcriptomic data, our goal was to determine the subcellular location of mRNA molecules for *CSPG4, PDGFRB*, or *HIGD1B* and assign different cell types to their locations in a normal human lung section. *CSPG4* or *PDGFRB* was located around large-sized vessels, while *HIGD1B* was mostly found in the parenchyma and tentatively around capillaries (Fig. 1C). By calculating the predictive value and specificity of *HIGD1B*, we found that *HIGD1B* exhibited a higher PPV when distinguishing annotated PCs from SMCs compared to *CSPG4* and *PDGFRB* (Fig. 1D). Although the sensitivity of *HIGD1B* to identify PCs was lower compared to other markers, its increased specificity and lower false negative rate suggest that although fewer PCs are identified when using *HIGD1B* as a PC-specific marker, it identified a “purer” population of PCs compared to *CSPG4* and *PDGFRB*. This makes *HIGD1B* a superior marker for studying PC gene expression and molecular changes under pathological conditions. A limitation of this bioinformatic analysis is the large volume of data from a single biological sample, the inability to perform repeat experiments in biological replicates, and the reliance on cell annotations from prior experiments. In addition, these markers may not distinguish between multiple subsets of PCs. Regardless, we provide evidence for using *HIGD1B* as a more specific PC marker. Future studies incorporating a combination of cell markers may enhance the characterization of PC subtypes and their distinction from other mural cells and stromal populations.

The HIG1 hypoxia inducible domain (HIGD) gene family comprises five genes: *HIGD11a, -1b, -1c, -2a, -2b* (Pang et al, 2021). *HIGD1a* is regulated by hypoxia-inducible factor-1 (HIF1) and promotes cell survival under hypoxic conditions (An et al, 2011; Bedo et al, 2005). Previous studies have indicated that *HIGD1B* promotes cardiomyocyte survival by maintaining mitochondrial integrity and its overexpression promotes cell survival through altered activation of caspase‑3 and ‑9, but its role in pulmonary cells is currently unknown (Pang et al, 2021). Notably, PCs in different organs reside at different locations throughout the capillary microcirculation, thus leading to distinct morphological characteristics and signature genes (Grant et al, 2019). Our previous work suggested that PC markers may be organ-specific and conserved in human and mouse hearts and lungs (Baek et al, 2022). The difference in PC gene expression between organs warrants further investigation, as specific gene expression patterns in PCs may control cell dedifferentiation and be an important mechanism to understand when developing PC-specific treatment strategies. Intriguingly, the *Higd1b-*CreERT2 mice truly reflect the PC specificity across various organs, with *Higd1bCre+* cells being abundant in not only hearts and lungs but also the brain, skeletal muscle, pancreas, intestine, connective tissue around the descending aorta, kidney, and retina. Although an in-depth analysis was not performed in each organ to determine the expression of *Pdgfrb* and *Cspg4* in tdT+ cells, our preliminary results suggested the potential of this novel mouse model to investigate PC biology beyond the cardiopulmonary system. Taken together, these results suggest *HIGD1B/Higd1b* is a superior cell marker to study the role of PCs in vascular diseases.

Due to their close origin, PCs giving rise to SMCs or other mural cells have been extensively studied in many organs during development and under pathological conditions. For example, PCs coordinate the behavior of epithelial and vascular cells during lung morphogenesis (Kato et al, 2018). PCs are a source of SMC precursors during collateral artery formation in heart development (Volz et al, 2015b). Resident PCs in postnatal skeletal muscle play a crucial role in contributing to differentiation in the smooth muscle layer of blood vessels and the development of skeletal muscle fibers (Dellavalle et al, 2011). FoxD1-lineage PC-like cells also contribute to the myofibroblast population following bleomycin-induced lung injury (Hung et al, 2013). PCs give rise to microglial cells after ischemic brain injury (Özen et al, 2014). PCs participate in vascular and fibrotic remodeling after ischemic damage in the heart (Quijada et al, 2023). PCs in kidneys differentiate into myofibroblasts, which contribute to collagen deposition and fibrosis (Chen et al, 2011; Crisan et al, 2008; Humphreys et al, 2010; Volz et al, 2015a). In contrast, some studies suggest that endogenous PCs in the heart, brain, skeletal muscle, and adipose tissue do not behave as multipotent tissue-resident progenitors (Guimarães-Camboa et al, 2017). These discrepancies may be due to different PC subtypes, suggesting a potential diversity of plasticity and function. However, the classification of proposed PC subtypes remains challenging, again, due to the lack of PC-specific markers. Without a consensus, PCs have been defined by the inclusion and exclusion of numerous markers. PCs in the cerebral circulatory system closer to the arteriole end of the capillary bed may be involved in regulating blood flow, while PCs on the capillary bed are more vital to maintaining the function of the blood-brain barrier, and PCs at the venule end of the capillary network regulate immune cell infiltration (Hall et al, 2014; Hartmann et al, 2015; Jespersen and Østergaard, 2012; Proebstl et al, 2012; Yemisci et al, 2009). Skeletal muscle Type 1 (Nestin-Ng2+) PCs are fibrogenic and adipogenic in old and diseased muscle, while Type 2 PCs (Nestin+Ng2+) generate new muscle tissue after injury (Birbrair et al, 2013). In addition, Type 2 PCs recover blood flow in a mouse model of hindlimb ischemia (Birbrair et al, 2014). Two types of PCs specified as CD274+ capillary and DLK1+ arteriolar PCs are differentiated from human pluripotent stem cells (Kumar et al, 2017). By applying scRNA-seq to IPAH and control lung cells, we identified two subgroups of PCs in the pulmonary vasculature (Fig. 5C). Type 1 are classical or synthetic PCs enriched with *HIGD1B* but with lower expression of SMC signature mRNAs, including *PDGFRB*, *ACTA2*, and *MYH11*. In mouse lungs, they have long punctate processes that partially wrap around capillary ECs within the basement membrane. They reside on capillary beds, with one cell covering roughly 3–4 capillary nets. Type 1 PCs maintain capillary homeostasis and inhibit EC proliferation under normal conditions. They are fully differentiated and quiescent, thus having fewer multipotent properties. Under Hx conditions, their process surface area may expand and wrap tighter around capillary EC junctions in response to arteriolar vasoconstriction, protecting against capillary leakage. Selective ablation of brain PCs provokes exuberant extension of processes from neighboring PCs to contact uncovered endothelium regions (Berthiaume et al, 2018). We speculated that Type 1 PC process expansion may be a dynamic event similar to brain PCs due to the absence/movement of neighboring Type 2 PCs, but it requires further experimental evidence.

Type 2 PCs are contractile with relatively lower expression of *HIGD1B* and higher expression of SMC signature mRNAs, including *PDGFRb*, *ACTA2*, and *MYH11*, compared to Type 1 PCs. In mouse lungs, they reside at the border zone of capillary nets and arterioles (15–25 μm). Their processes may be shorter, making detachment and transmigration easier. Similar to Type 1 PC inhibition to EC growth, Type 2 PC’s main functions may prevent arteriolar SMCs from migrating to capillaries and maintain the homeostasis balance of the SMC-PC-capillary compartments. Under Hx conditions, Type 2 PCs translocate out of the border zone and reside on arterioles (diameter >25 μm) and coexpress Sma and Smmhc, suggesting they have multipotent properties under pathological conditions and may be more motile and act like SMCs in response to arteriolar vasoconstriction. In a recovery model where mice are re-exposed to normoxia after 3 wks of Hx, PH is completely reversed. Intriguingly, the vast majority of Type 2 PCs are no longer required for remodeling and vasoconstriction and move back to the border zone, while Type 1 PC processes are likely to restore to their normal size. Vim orchestrates cytoskeletal and microtubule rearrangements and mechano-signaling to promote cell migration and polarity (Gan et al, 2016; Jiu et al, 2015; Mendez et al, 2010). Its upregulation in Hx Type 2 PCs may reduce cell–cell contact and drive cell translocation. To supplement our scRNA-seq and tissue staining experiments, we performed in vitro experiments on healthy human lung PCs and found the overexpression of VIM in both normoxic and Hx conditions results in cell adhesion, migration, and the upregulation of SM22 expression. Taken together, these findings suggest that Vim expression in PCs may contribute to the development of Hx-induced PH.

More studies are required to distinguish Type 1 and Type 2 PCs using unique markers and to further characterize and validate their function and plasticity. Their cellular and genetic identities should be further validated using clonal expansion and multiple color reporter systems, such as Confetti reporter lines. In addition, subtypes of PCs in the capillary circulation of human lungs need to be carefully examined and characterized. Despite its advantages, scRNA-seq does not describe cell location and spatial information. Understanding PC spatial orientation is crucial for comprehending PC distribution or morphological changes in disease pathogenesis, especially if we can identify them in biopsy samples in relation to disease progression and severity. The other limitation is that the geographic differences of human donor PCs regarding sex, age, and ethnicity are not explored. To more specifically label PC subtypes, barcoding cells with synthetic DNA sequences such as DARLIN, an inducible Cas9 barcoding mouse line, may be a useful tool to generate massive lineage barcodes across tissues and enable the detection of edited barcodes in profiled single cells (Toyama et al, 2023). Only the C57BL/6 strain was used for lineage tracing, as different strains exhibit varying levels of hemodynamic responses to Hx (Ikeda et al, 2019; Tada et al, 2008). PC lineage changes in other lung diseases such as chronic obstructive pulmonary disease ^(COPD), interstitial lung disease (ILD), idiopathic pulmonary fibrosis (IPF), and asthma should be carefully examined and investigated. In addition, the role of PCs in the heart microvasculature is overlooked under pathological conditions. Targeting PC malfunction reveals enormous therapeutic possibilities, as PC-directed therapies have the potential to reverse or prevent disease progression and development in multiple scenarios. For example, human pluripotent stem cell-derived PCs have been successfully engrafted into the vasculature of ischemic murine limbs and promoted vascularization and muscle regeneration, highlighting the high ceiling of their potential as a therapeutic target (Dar et al, 2012). The proliferation of SMCs in PAH may be targeted using a PDGFRβ inhibitor (such as imatinib) (Frost et al, 2015; Ghofrani et al, 2010). However, PDGFRβ is expressed not only on SMCs but also on PCs, which play a crucial role in maintaining capillary homeostasis. Therefore, reducing the PC population with a PDGFRβ inhibitor may disrupt capillary homeostasis, leading to unexpected side effects and vascular leakage. To specifically target SMC proliferation, it may be more effective to use surface markers that are more specific to SMCs and exclude PC inhibition.

In summary, we identified *HIGD1B*/*Higd1b* as a specific and unique cell marker able to differentiate PCs from other mural cell populations. We generated a novel, tamoxifen-inducible, PC reporter mouse *Higd1b-*CreERT2 and confirmed the specific and appropriate labeling of PCs dominantly in the lung,  heart, and other organs. Our study, for the first time, suggests the existence of PC subtypes in the pulmonary circulation with different functions and dynamic changes in their morphologies under Hx conditions. This new cell marker and knockin mouse will provide the field of vascular biology with the necessary tools to perform fate mapping and gene knock-out experiments specific for PCs and lead to novel discoveries on their contribution to disease development.

## Methods

### *HIGD1B* expression in single-cell RNAseq annotating PCs

We employed integrated data from the ‘Human Lung Cell Atlas (core)’ and the ‘Tabula Muris Senis’ to investigate the expression of *CSPG4*, *PDGFRB*, and *HIGD1B* in PCs of human and mouse lungs, respectively (Tabula Muris, 2020; Travaglini et al, 2020). In addition, single-cell sequencing data from Litviňuková et al (2020) and Feng et al (2022) were utilized for analogous investigations in human and mouse hearts (Feng et al, 2022; Litviňuková et al, 2020). These data, which included annotations for PCs, were derived from healthy subjects or mice. We used the Seurat R package to specifically examine the expression of *CSPG4*, *PDGFRB*, and *HIGD1B* across different cell types utilizing dot plots and density plots, employing the original UMAP coordinates from each data for visualization (Jain et al, 2021). The analysis methods applied for Fig. 5C–F were previously published in PMID: 38243138.

### Spatial analysis of *HIGD1B* in lung tissue and quantifications

We analyzed the ‘Non-diseased Lung’ data which consists of consists of 295,883 cells from the ‘Xeniµm Human Lung Preview Data’ to examine *CSPG4*, *PDGFRB*, and *HIGD1B* expression in lung tissue. The data underwent normalization with SCTransform, followed by dimensionality reduction using Principal Component Analysis (PCA) (Choudhary and Satija, 2022; Jain et al, 2021). We selected the top 60 principal components based on the percentage of variance explained for subsequent analysis. These components were used to project the cells into a two-dimensional space using the Uniform Manifold Approximation and Projection (UMAP) algorithm (Becht et al, 2018). Unsupervised clustering was performed with the FindClusters function (resolution set at 0.3), identifying 23 clusters (Jain et al, 2021). To annotate the cell types in the data, we utilized ‘singleR’ and HLCA as a reference to computationally annotate the cell types. However, as the data is limited to a gene panel consisting of only 541 genes, we used well-established markers for cell types of interest and genes identified through DE analysis between the clusters to annotate cell types. To determine the final cell type annotation, we employed a consensus-based approach. Specifically, we assigned a final cell type when at least two methods agreed. When the approaches provided similar but not identical annotations, we utilized a broader, more general category to harmonize the classification.

For quantification of the spatial transcriptomics, four sections (100 × 2500) were randomly selected from three larger lung areas (coordinates: x: 3750–4550, y: 1200–2900; x: 5200–6000, y: 3300–5000; x: 9700–10500, y: 5800–7500). Cells of interest (PCs, SMCs, and all other cells) within these sections were color-coded according to their cell types and molecules of interest (*CSPG4, PDGFRB*, and *HIGD1B*). One individual blinded to the cell populations quantified the expression of each molecule (or lack thereof) in the three groups. A cell with two distinct molecules was counted twice, once in each group, and those with expression of the same molecule more than once were only counted once. Within the four frames inspected, 1312 cells were identified (PCs: 66, SMCs: 106). *HIGD1B* was identified on 49 cells, *CSPG4* on 105 cells, and *PDGFRB* on 229 cells. Equations used to calculate the predictive value and accuracy of each cell marker are as follows: sensitivity: (TP/(TP + FP)), specificity: (TN/(TN + FP)), positive predictive value: (TP/(TP + FP)), negative predictive value: (FP/(FP + TP)) and accuracy (TP + TN)/(TP + TN + FP + FN).

### Sub-clustering pericyte cluster utilizing Harmony

The pericyte cluster, annotated from respiratory airway and lung parenchyma tissue was extracted from the ‘Human Lung Cell Atlas (core)’. To mitigate the effects of potential confounders, we first removed data from ‘Studies’ that had less than twenty pericytes and utilized Harmony to adjust for variables including ‘data’, ‘assay’, ‘tissue sampling method’. ‘sequencing platform’, ‘development stage’, ‘tissue’, ‘subject type’, ‘study’, ‘lung condition’, ‘sex’, ‘self-reported ethnicity’ and ‘age or mean of age range’ (Korsunsky et al, 2019). We applied PCA to this focused data, selecting the top fifteen principal components based on their contribution to variance. These principal components were subsequently used to project the cells into a two-dimensional space via the UMAP algorithm. Following this, unsupervised clustering was conducted using Seurat’s FindClusters function, with the resolution parameter set to 0.5. We then utilized dot and density plots to investigate the expression patterns of *CSPG4*, *PDGFRB*, and *HIGD1B* across these Pericyte sub-clusters.

### Differential gene (DE) expression analysis and gene set enrichment analysis (GSEA)

DE analysis was carried out to compare Pericyte sub-clusters 0 and 3 using the Wilcoxon rank-sum test (Jain et al, 2021). In this analysis, a positive log2 fold change (log2FC) indicates higher gene expression in sub-cluster 0 relative to sub-cluster 3. DE genes were ranked in descending order based on their log2FC to create a pre-ranked gene list for subsequent analysis. We employed the GSEApy to perform GSEA on this pre-ranked gene list against curated gene sets from the Hallmark collection, and the Kyoto Encyclopedia of Genes and Genomes (KEGG) (Subramanian et al, 2005). Statistical significance for pathway enrichment was assessed using a permutation test, with pathways exhibiting an adjusted q-value below 0.05 considered statically significant.

### Experimental animals

All animal procedures conducted in this study were approved by the Institutional Animal Care and Use Committee (IACUC) guidelines at Boston Children’s Hospital (BCH) and adhered to the published guidelines of the National Institutes of Health (NIH) on the use of laboratory animals. Mice were housed in the animal facility at BCH with a 12-h light/dark cycle and ad libitum access to rodent chow and water.

The founder #25 *Higd1b-*CreERT2 was crossbred with Ai14 (*Higd1b-*CreERT2:*:R26-tdTomato*) for generation of the *Higd1b-tdT* and also crossbred with the *Rosa26-mTmG* (https://www.jax.org/strain/007576) to generate *Higd1b-mTmG*. Ear clip-based genotyping was used to identify knockin and control mice. *Cspg4-CreERTM* (https://www.jax.org/strain/008538) was crossbred with Ai14 for the generation of the *Ng2-tdT* or *Rosa26-mTmG* for the generation of the *Ng2-mTmG* and tamoxifen induction following the previously published protocol (Yuan et al, 2020). *Pdgfrb*-CreERT2 (Jax Strain #029684) was crossbred with Ai14 to generate the *Pdgfrb-tdT* and its tdT+ label was endogenous without tamoxifen injection.

### Donor plasmid and guide RNA

Higdb1-P2A-CRE-ERT2 targeting vector (donor) was generated by assembling 1 kb left (LHA) and 1 kb right homology (RHA) arms flaked by P2A-CRE-ERT2 (Fig. 1A) and designed and purchased by Vectorbuilder Inc (cat# VB220531-1072sdb). Two gRNA (27F & 37F) were used for CRISPR/Cas9 mediated knock-in into Higdb1 exon 4. 0.61 pmol each of crRNA and tracrRNA was conjugated and incubated with Cas9 protein (30 ng/μl) to prepare ribonucleoprotein complex (RNP) according to Aida et al and donor DNA (10 ng/μl) was mixed with RNP for microinjection cocktail.

### Microinjection

For knock-in mouse generation - microinjection cocktail was injected into 0.5 dpc embryos harvested after mating C57Bl6-Hsd (Envigo). Post-injection embryos were reimplanted into CD1 (Envigo) pseudo-pregnant foster females and allowed to term. Tail snip biopsies were collected from pups at P7.

### Genotyping

Tail snip genomic DNA was prepared from pups and analyzed by PCR using the following primer pairs (Fig. 2C). Primer pairs LF + C1 for LHA, C2 + C3 for Cre, C4 + RR for RHA. PCR reaction containing 0.5 μM each primer pair, 1xQ5 MM buffer (NEB), 100 ng genomic DNA and was amplified using a thermocycler (BioRad): 95 °C - 3 min 95 °C - 30 s annealing 67 to 72 °C, 72 °C 1 min for 35 cycles and final extension at 72 °C for 5 min. PCR products were analyzed on 1% agarose gel (Seakem - GTG), 1XTAE buffer, and DNA was purified from gel using a Gel Extraction kit (QIAQuick - Qiagen) and cloned using TOPO cloning Kit (Invitrogen).

Plasmid DNA was prepared using miniprep kit (Qiagen) and Sanger sequenced using primers: S1 forward primer outside of LHA and S2 reverse primer at the 5’ of CRE; S3 forward primer at the 3’ of CRE, S4 forward primer in ERT2 and S5 reverse primer outside of RHA.

### Reporter gene activation via tamoxifen injection

For tamoxifen induction, *Higd1b-tdT* mice were injected via intraperitoneal injection with a total dose of 0.2 mg/g body weight tamoxifen dissolved in corn oil (20 mg/ml) over 2 days. The median age and weight of *Higd1b-tdT* male mice used in this study was 7.47 +/*−* 1.22 weeks and the average body weight 28.14 +/*−* 1.17 g at the time of injection. The median age of female mice was 7.35 +/*−* 1.22 weeks and the average body weight 28.1 +/*−* 1.17 g.

*Higd1b-mTmG* mice were injected via intraperitoneal injection with a total dose of 0.2 mg/g body weight tamoxifen dissolved in corn oil (20 mg/ml) over 2 days. The median age and weight of *Higd1b-mTmG* male mice used in this study was 7.55 +/*−* 1.04 weeks and the average body weight 28.53 +/*−* 1.2 g. The median age of female mice was 7.86 +/*−* 1.04 weeks and the average body weight 28.55 +/*−* 1.2 g at the time of injection.

All mice were allowed to rest for 7–14 days before being exposed to Hx or undergoing tissue harvest. As negative controls, tissue from mice (*Higd1b-tdT*+*/−* and *Higd1b-mTmG*+*/−*) without tamoxifen and wild-type mice (Cre positive flox negative or Cre negative flox positive as *WT-tdT*+*/−* and *WT-mTmG*+*/−*) injected with similar doses of tamoxifen were harvested and underwent inspection for endogenous PC labeling. Experimental animals are defined as heterozygous of Cre and flox.

### RNAscope

RNAscope experiments for *Higd1b* was conducted and provided by the Neurobiology Imaging Facility (NIF) at Harvard Medical School, Boston, MA.

### Hypoxia Studies

Mice were placed in an Hx chamber and exposed to 10% FiO_2_ with ad libitum access to rodent chow and water for up to 3 weeks. The environment within the chamber was established through a continuous mixture of room air and nitrogen gas. The chamber environment was continuously monitored using an oxygen analyzer (Servomex, Sugar Land, TX). CO_2_ was removed with lime granules, and the temperature was maintained between 22 and 24 °C. The chamber was inspected at least daily for animal welfare, O_2_ concentration, CO_2_ concentration, and humidity.

### Vibratome tissue preparation

Animals were euthanized with controlled isoflurane and cervical dislocation. After euthanasia, mice were secured in the supine position, and a midline incision was made to expose the abdominal and thoracic cavities. The sternum was dissected to expose the contents of the mediastinum and then the abdominal aorta was located and severed. A 25 G butterfly needle was inserted into the right ventricle (RV) and slowly perfused with 15cc of ice-cold 1X phosphate-buffered saline (PBS) to flush the red blood cells from the circulatory system. Once the lungs were white in appearance, the trachea was cannulated and the lungs inflated with 2% low-melting point agarose in 1X PBS. After complete inflation, the trachea was tied, and cold 1x PBS was poured over the lungs to solidify the agarose and preserve the structure of the lung. The lungs were then carefully removed from the mediastinum and placed in 4% paraformaldehyde (PFA) at 4 °C overnight, followed by washing in 1X PBS the next day. Lung lobes were then separated and sectioned with a vibratome machine (Leica VT1000 S) at a thickness of 300 µm for IF staining and microscopy (Klouda et al, 2021).

The heart was removed from the mediastinum after flushing with 15cc of 1X PBS and the right and left atrium dissected to expose ventricles. Heart samples were washed in 1X PBS and then fixed in 4% PFA overnight at 4 °C. After three washes in 1X PBS (one hour each), the samples were placed at 4 °C overnight on a rotating plate. The next day, samples were removed and sectioned with a vibratome machine at a thickness of 300 µm.

### Optical cutting temperature (OCT) tissue preparation

After appropriate euthanasia, the hind leg of the mice was secured and an incision was made into the subcutaneous tissue located over the femur to expose the muscle. Skeletal muscle from the femur, identified by its striations and orientation of muscle fibers, was carefully dissected and removed. For connective tissue preparation, the tissue surrounding the descending aorta in the abdominal cavity was identified and dissected after removing the mediastinal contents as previously described. (Kim et al, 2024) Connective tissue and skeletal muscle were placed in 4% PFA at 4 °C overnight and then washed in 1X PBS for another day.

Brain tissue was harvested from mice by first securing the mouse in the prone position after euthanasia and dissecting away the hair and subcutaneous tissue to expose the skull. The skull was carefully pierced at the estimated location of the sagittal suture, and the cranial bones were dissected, being careful not to damage the brain tissue underneath. Once an opening was created and the brain removed from the skull, the tissue was placed in 4% PFA at 4 °C overnight. Tissues were washed in 1X PBS for an additional day.

The liver and kidney were located and carefully removed after euthanasia and flushing of red blood cells from the vasculature. The tissue was fixed in 4% PFA at 4 °C overnight and then washed in 1X PBS.

All the above samples were completely submerged in a 30% sucrose/PBS solution and placed at 4 °C overnight on a rotating plate for several days until they sunk to the bottom. They were embedded with 100% OCT and stored at −80 °C for future experiments. OCT-prepared samples were then sectioned with a Cryostat machine (RWD, FS800A Cryostats) at a thickness of 10 μm and mounted on microscopy slides for IF staining.

The heart was removed from the mediastinum after flushing with 15cc of 1X PBS and the right and left atrium dissected to expose ventricles. Heart samples were embedded in 100% OCT solution. The next day, samples were removed and sectioned with a Cyrostat machine at a thickness of 10 µm.

Retinas were dissected from mice after euthanasia and fixed in 4% PFA for one hour and then washed with 1x PBS overnight at 4 °C. The samples were carefully dissected under a dissecting microscope to expose the optic nerve and surrounding vasculature. Fixed retinas were stored at 4 °C until IF staining. After staining, the samples were placed on microscopy slides with Prolong Gold Antifade Solution containing DAPI.

### Cell culture and VIM transfection

Human lung PCs collected from our PC biobank. Three healthy PCs isolated from failed donor tissues from our previously established PC biobank were utilized for all cell culture experiments (PMID: 25447046). PCs were grown in PC media (ScienCell, Cat# 1001) with growth supplements and used between passages 4 to 15.

### Plasmids

EGFP-Vimentin-7 was used for VIM overexpression, which was a gift from Michael Davidson (Addgene plasmid # 56439; http://n2t.net/addgene:56439; RRID:Addgene_56439), or pcDNA3.1-empty vectors for control in a transfection device (Nucleofector II Program U-052; Lonza) using the basic SMC Nucleofector kit (Lonza). All experiments were performed in normoxic and hypoxic (FiO_2_:2.5%) conditions.

### Cell adhesion assay

5 × 10^5^ PCs with empty vector or with VIM-vector were seeded on the MatTek uncoated 8 well slides (Cat # CCS-8). Each slide was under normoxia or 2.5% hypoxia chamber (Thermofisher) for 24 h. Then, images were acquired using phase-contrast using Leica Thunder Imager.

### Cell ICC staining

Around 5 × 10^5^ cells were seeded on eight-well cell culture slides (Cat.No:80806 ibidi GmbH) on the day before the staining. Next, cells were fixed for 15 min in 4% paraformaldehyde, followed by three washes with PBS. Cells were then permeabilized with ice-cold 1× PBS containing 0.1% Triton X-100 and goat serum for 1 h, followed by overnight incubation with primary antibodies at 4 °C, washed in 1× PBS, and incubated with Alexa Fluor 594 goat anti-mouse antibody and/or Alexa Fluor 488 goat anti-rabbit (A11008; Invitrogen) accordingly for 1 h at room temperature. Slides were mounted with Prolong Gold antifade solution containing DAPI (Invitrogen).

### Wound-healing co-culture assay

Cell-culture plates with inserts (catalog number 81176) were purchased from ibidi GmbH (Munich, Germany). Around 2 × 10^4^ cells were seeded into either side of the inserts. Cells were synchronized using starvation media for 16 h before removal of the inserts. Serial images of the gap between PCs were taken over a period of 6 h. Cell migration rate and orientation were calculated by comparing cells at 0 and 6 h. Phalloidin-labeled F-actin (1:50; 8878, Invitrogen) was used to stain actin filaments.

### FACS flow cytometry

Lung tissues from control and *Higd1b-tdT* mice were prepared for FACS analysis. The day before the analysis, 25 μL of sheep anti-rat IgG Dynabeads (11035; Invitrogen, Carlsbad, CA) were incubated overnight with 10 μg of CD45 IgG. The next day, fresh lung tissue was washed twice with 1× PBS to remove any residual medium. The tissue was then minced and digested using a Miltenyi GentleMACS dissociator (Miltenyi Biotec, Germany). After dissociation, 10 mL of cold PBS containing 5% FBS was immediately added. The suspension was passed through a BD Falcon 70-μm cell strainer (BD Biosciences, San Jose, CA) to remove debris and undigested fibrous tissue. For control cells, wash three times with 1× PBS, then keep on ice. For Higd1b-tdT cells, the cell pellet was resuspended in 1 mL of 1× PBS containing 35 μL of CD45 IgG-coated magnetic beads and gently rotated at 4 °C for 20 min. The CD45 beads were then depleted using a magnet. The cells were washed three times with 1× PBS and stained with NG2 (sc-166251, Santa Cruz) and PDGFRB (14-1402-82, Invitrogen) antibodies, incubating at 4 °C for 15 min. Following this, the cells were washed three times with 1× PBS and incubated with Alexa Fluor 488 donkey anti-mouse antibody (A-21202, Invitrogen) and/or Alexa Fluor 647 goat anti-rat antibody (A-21245, Invitrogen) for 15 min at 4 °C. Finally, the cells were washed with PBS and immediately analyzed by flow cytometry using a BD LSRFortessa™ cell analyzer.

### Tissue immunofluorescence staining

Vibratome-prepared precision cut lung sections (PCLSs) and heart tissues were blocked with 5% goat serum in 0.5% Triton X-100/PBS (PBS-T) for one hour at room temperature. Samples were incubated with primary antibodies diluted in 5% goat or donkey serum and 0.5% PBS-T at 4 °C for 24 h. PCLSs were then washed three times in 1X PBS (fifteen minutes per wash) followed by incubation with alexa 488, 555, or 647 fluorophores containing secondary antibodies (1:250 concentration) overnight at 4 °C. Samples were then washed again and placed on microscopy slides with Prolong Gold Antifade Solution containing DAPI (Life Technologies Corporation). After thawing to room temperature, OCT-prepared tissue and retina were stained following a similar technique. All images were captured using a Zeiss confocal 880 Airyscan microscope and processed by Aivia software.

The following antibodies were used for IF staining:

Mouse-anti-mouse/human SMA-647 (1:100; sc-32251-AF647, Santa Cruz Biotechnology)

Rat-anti-mouse CD31 (1:100; 553370, BD-Pharmingen)

Rat-anti-mouse PDGFRβ (1:100; 14-1402-82, Invitrogen)

Rabbit-anti-mouse MYH11 (1:100; AB53219, ABCAM)

Isolectin GS-IB_4_-488 (1:100; I21411, Thermo Fisher Scientific/Invitrogen)

Goat-anti-mouse Cardiac Troponin 1 (1:100 ab56357, Abcam)

Rat-anti-mouse CD326 (1:100, 870481, Cell Signaling)

APC-mouse CD11c (1:100, 117310, BioLegend)

Rabbit-anti-mouse MHCII (1:100; Ab133567, Abcam)

Rat-anti-mouse/human Ly6G (1:100, 127643, BioLegend)

Rabbit-anti-mouse/human Ki67 (1:300, ab16667, Abcam)

Mouse-anti-rat/human 3G5 (1 mg/ml; in house-made)

Rabbit-anti-human SM22 (1:100, ab14106, Abcam)

Rabbit-anti-human Vimentin (1:100, 5741S, Cell signaling)

### Quantifications of tdT+ PCs in lungs and other organ systems of *Higd1b-tdT*

To quantify the number of *Higd1b*+ cells expressing commonly used mural cell markers Pdgfrβ and Ng2, we performed IF staining on PCLSs from three *Higd1b-tdT* normoxic mice (2 male and 1 female). Four images containing at least 30 pericytes each were obtained from each sample at different locations by a blinded individual under similar microscope settings. Z-stack and Aivia software were used to delineate the spatial arrangement and stain cells. The expression of both molecules (Pdgfrβ and Ng2) in *Higd1b*+ pericytes was calculated by dividing the number of tdT+ cells coexpressing either marker by the total of tdT+ and DAPI+ double-positive cells counted.

After harvest, solid organs of interest from *Higd1b-tdT*+*/−* mice were stained for endothelium with either Cd31 or Isolectin(retina) and Sma to identify SMCs. One image was taken from a random location from each sample at similar magnification and size. Image J software was used to quantify the number of tdT+ cells in each sample per image field by using Otsu thresholding method for consistency.

To quantify the location of tdT+ cells from *Higd1b-tdT*+*/−* mice in normoxic and Hx conditions, lung tissue was stained for endotheliµm (Cd31) and SMCs (Sma). Arteries were characterized as vessels >50 µm expressing Cd31 and Sma in a continuous pattern. Veins were characterized as being >50 µm and expressing Cd31 with a patchy accumulation of Sma. Lastly, capillaries were defined as Cd31+, Sma- vessels less than 25 µm in size. Images from each mouse (Normoxia *N* = 3, 2 males and 1 female; Hx *N* = 3, 1 male and 2 female) were taken under similar microscope settings by one blinded individual. A different investigator reviewed images with Aivia software and recorded the number of tdT+ PCs interacting with each cell type. The percent of cells was calculated by dividing the number of cells in direct contact with the vessel type (artery, vein, and capillary) by the total number of tdt+ cells.

To determine if PCs underwent proliferation in response to Hx, we quantified the number of tdT+ cells coexpressing Ki67 by performing IF staining on PCLSs from *Higd1b-tdT* normoxic (*N* = 2, 1 male and 1 female) and 3 wk Hx (*N* = 3, 1 male and 2 females). Four images containing at least 30 PCs were obtained from each sample at different locations by a blinded individual under similar microscope settings. Z-stack and Aivia software was used to delineate the spatial arrangement of IF staining. The expression of Ki67 in *Higd1b*+ pericytes was calculated by dividing the number of cells coexpressing Ki67 by the total of tdT+ cells counted.

To quantify the number of *tdT*+ cells expressing Sma, we performed IF staining on PCLSs from three *Higd1b-tdT*+*/−* normoxic mice and three mice exposed to 3 wks Hx. Four images containing at least 30 PCs each were obtained from each sample at different locations by a blinded individual under similar microscope settings. Z-stack and Aivia software were used to better delineate the spatial arrangement and staining of cells. The expression of Sma in tdT+ cells was calculated by dividing the number of cells coexpressing Sma by the total number of tdT+ cells visualized. To capture the morphological changes of PCs in response to Hx, high-resolution images at increased magnification focusing on the distal arterioles were taken from three mice exposed to 1 wk, 2 wk, and 3 wks Hx.

To characterize the expression of vimentin in tdT+ PCs, PCLSs from *Higd1b-tdT*+*/−* mice under normoxic (*N* = 2, 1 male and 1 female) and Hx conditions (N:3, 1 male and 2 females) were stained for vimentin. Four images were randomly taken from each sample under similar magnification and settings by one blinded individual. Each image contained a minimum of 30 pericytes. Z-stack and Aivia software were used to better describe the shape and spatial arrangement of cells. The percentage of cells expressing vimentin was calculated by dividing the number of vimentin-positive Higd1b-positive cells by the total number of tdT+ cells counted.

### Statistical analysis

Values from multiple experiments are expressed as means ± SEM. Statistical significance was determined using either an unpaired t-test or one-way analysis of variance followed by Bonferroni multiple comparison tests unless stated otherwise. *P* < 0.05 was considered significant.

## Supplementary information

Appendix

Peer Review File

Source data Fig. 2

Source data Fig. 3

Source data Fig. 4

Source data Fig. 6

Source data Fig. 7

EV and Appendix Figure Source Data

Expanded View Figures

## Data Availability

For experiments pertaining to scRNA-seq, microfluid, droplet based data was processed using the 10× Genomics platform (10×3′v2) of the (1) Tabula Muris Senis, (2) Human Lung Cell Atlas, (3) Adult Human Heart, and (4) The Mouse Heart data, which were obtained from the Cellxgene collections (https://cellxgene.cziscience.com). The pre-processed, publically available scRNA-seq data utilized in experiments include: 1. Tabula Muris Senis compendium for murine lung tissue: (https://cellxgene.cziscience.com/collections/0b9d8a04-bb9d-44da-aa27-705bb65b54eb). 2. The Human Lung Cell Atlas data for human lung tissue: (https://cellxgene.cziscience.com/collections/5d445965-6f1a-4b68-ba3a-b8f765155d3a). 3. The Adult Human Heart data for human cardiac cells: (https://cellxgene.cziscience.com/collections/b52eb423-5d0d-4645-b217-e1c6d38b2e72). 4. The Mouse Heart data for murine cardiac cells: https://www.ncbi.nlm.nih.gov/geo/query/acc.cgi?acc=GSE193346. Spatial transcriptomics of the human lung was obtained from the Xenium Human Lung Preview Data (Non-diseased Lung) using 10x Genomics Data which can be found at: (https://www.10xgenomics.com/data/xenium-human-lung-preview-data-1-standard). The source data of this paper are collected in the following database record: biostudies:S-SCDT-10_1038-S44318-024-00349-1.
