# Supplementary material for: Specialized pericyte subtypes in the pulmonary capillaries
Source: EMBO J. 2025 Jan 13;44(4):1074–106. doi: 10.1038/s44318-024-00349-1 (PMC11833098; doi:10.1038/s44318-024-00349-1)
Supplement: Supplementary file 1 — Appendix [file 44318_2024_349_MOESM1_ESM.pdf]

Appendix

Specialized Pericyte Subtypes in the Pulmonary Capillary

Timothy Klouda[1*], Yunhye Kim[1*], Seung-Han Baek[1*], Mantu Bhaumik[2], Yan Li[1], Yu Liu[3], Joseph C Wu[3], Benjamin A Raby[1], Vinicio de Jesus Perez[4 #], Ke Yuan[1#]

[1]Division of Pulmonary Medicine, Boston Children's Hospital, Boston, MA 02115, USA

[2]Department of Neurology, F.M. Kirby Neurobiology Center, Boston Children's Hospital and Harvard Medical School, Boston, MA, USA.
[3]Stanford Cardiovascular Institute, Division of Cardiovascular Medicine, Department of Medicine, Stanford University School of Medicine, Stanford, CA, 94304, USA.
[4]Division of Pulmonary and Allergy Critical Care Medicine, School of Medicine, Stanford University, Palo Alto, CA, USA

*Authors contributed equally to this work.
#Authors contributed equally to this work.
Co-correspondence: vdejesus@stanford.edu
Lead-correspondence: ke.yuan@childrens.harvard.edu

Table of contents

Appendix Figure S1: *Higd1b* is expressed in murine lung PCs. Page 1

Appendix Figure S2: *HIGD1B* is not exclusively expressed in human heart PCs. Page 3

Appendix Figure S3: *Higd1b* is not exclusively expressed in murine heart PCs. Page 4

Appendix Figure S4: Potential PC-specific cell markers are identified from differentially expressed genes in PCs across human and murine lung and heart scRNA-seq data. Page 6

Appendix Figure S5: Cell types are annotated in spatial transcriptomic data utilizing known markers. Page 7

Appendix Figure S6: Cell types are annotated in spatial transcriptomic data utilizing computational cell type annotation and differentially expressed (DE) genes. Page 9

Appendix Figure S7: Spatial analysis of PC markers in non-diseased lung tissue. Page 11

Appendix Figure S8: *Higd1b* mRNA expression is absent in arterial SMC layers. Page 12

Appendix Figure S9: tdT+ cells from *Higd1b*-tdT lungs coexpress Pdgfrβ and Ng2 by FACS. Page 13

Appendix Figure S10: GFP+ cells from *Higd1b*-mTmG+/- mice do not express epithelial or immune cell markers. Page 14

Appendix Figure S11: Tamoxifen is not injected into *Higd1b*-tdT+/- and *Higd1b*-mTmG+/- or tamoxifen injection on WT littermates. Page 15

Appendix Figure S12: Pdgfrβ and Ng2 are non-specific markers for lung PCs. Page 16

Appendix Figure S13: tdT+ PCs do not co-express Ki67 in response to chronic Hx. Page 17

Appendix Figure S14: tdT+ cells accumulate in muscularized distal arterioles after 3wk Hx. Page 18

Appendix Figure S15: Sub-clustering of PCs was conducted from HLCA. Page 19

Appendix Figure S16: Gene Set Enrichment Analysis in PC sub-cluster 0 vs 2. Page 20

Appendix Figure S17: Mural cell cluster 5 and 6 feature plots from IPAH and control scRNA-seq data. Page 21

Appendix Figure S18: Overexpression of VIM in PCs does not enhance proliferation. Page 22

Appendix Figure S1

A

B

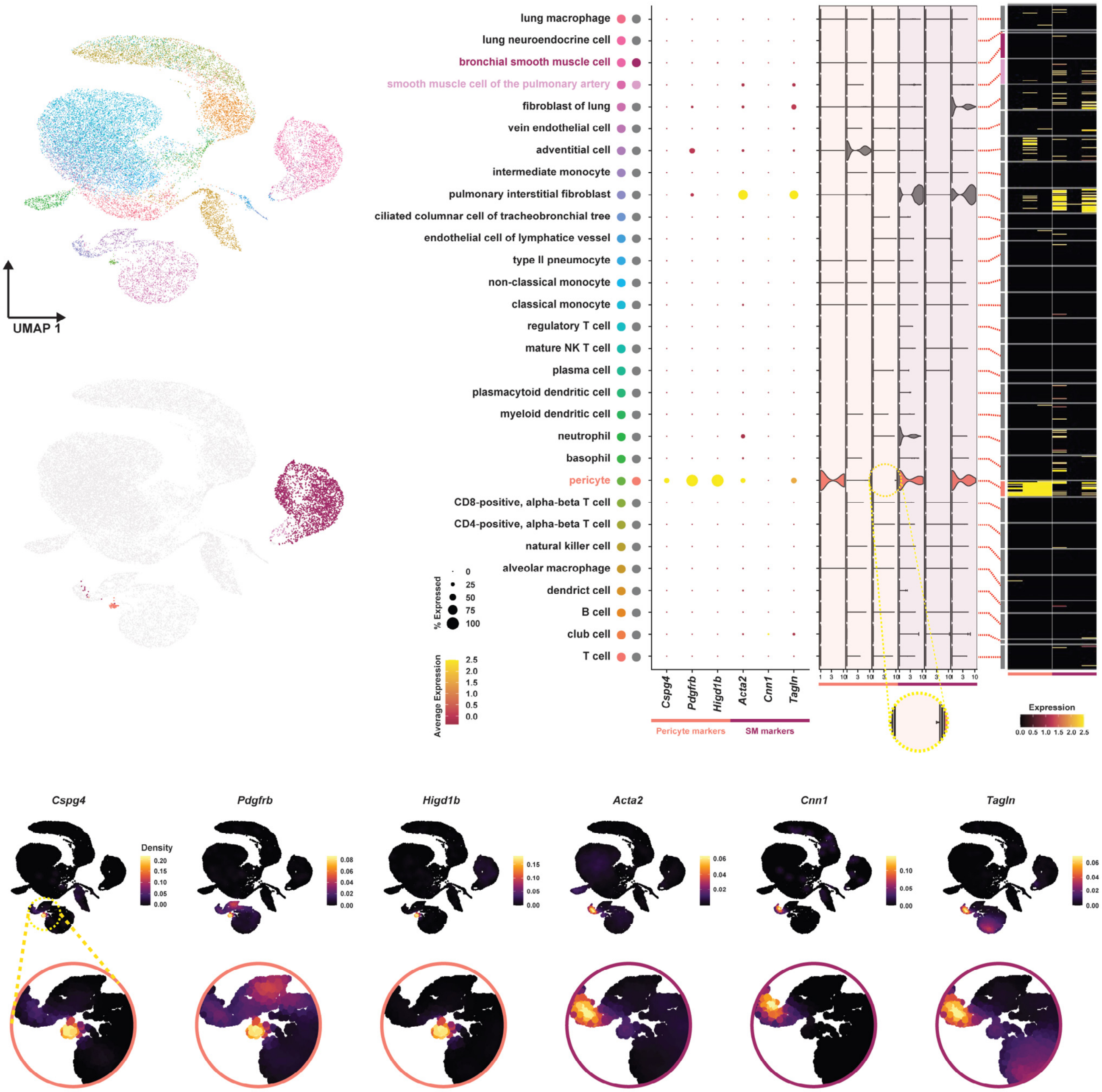

C

	pericyte vs Others				bronchial smooth muscle cell vs Others				smooth muscle cell of the pulmonary artery vs Others			
	Ave log2FC	padj	pct.1	pct.2	Ave log2FC	padj	pct.1	pct.2	Ave log2FC	padj	pct.1	pct.2
Cspg4	8.232	padj < 1E-04	39.3%	0.1%	-0.071	1	0.2%	0.2%	-5.657	1	0%	0.2%
Pdgfrb	9.711	padj < 1E-04	96.7%	2.2%	-4.560	0.002	0.9%	2.6%	-5.391	1	1.0%	2.5%
Higd1b	10.160	padj < 1E-04	100%	0.8%	1.124	padj < 1E-04	3.9%	0.7%	-6.194	1	0%	1.0%
Acta2	5.696	padj < 1E-04	36%	6.3%	1.607	padj < 1E-04	2.4%	6.7%	0.042	0.071	17.3%	6.3%
Cnn1	-5.096	1	0%	0.2%	2.802	padj < 1E-04	0.7%	0.1%	-5.098	1	0%	0.2%
Tagln	4.315	padj < 1E-04	42.6%	3.8%	1.486	1	2.7%	4.0%	0.144	padj < 1E-04	19.4%	3.8%

Others (n = 24,479): dataset excluding pericyte, Others (n = 22,201): dataset excluding bronchial smooth muscle cell, Others (n = 24,442): dataset excluding smooth muscle cell of the pulmonary artery
Tabula Muris Senis Normal Lung (n = 24,540 cells), pericyte (n = 61), bronchial smooth muscle cell (n = 2,339), & smooth muscle cell of the pulmonary artery (n = 98)

Appendix Figure S1: *Higd1b* is expressed in murine lung PCs.

(A) UMAP visualization of all cell populations within mouse lung tissue based on original UMAP coordinates and cell type annotations from the *Tabula Muris Senis* compendium. Cell type annotations are color-coded, as shown in the dot plot. PC and SMC distributions within lung tissues, using original UMAP coordinates and cell annotations from the *Tabula Muris Senis* compendium are highlighted in the bottom left. In UMAP, Dot, Violin and Heatmap plots, PCs were highlighted in light orange, bronchial SMCs in dark red, and SMCs of the pulmonary artery in pink. The Dot and Violin plots on the right display the expression patterns of PC (*Cspg4, Pdgfrb,* and *Higd1b*) and SMC markers (*Acta2*, *Cnn1*, and *Tagln*) across all annotated cell types. Consistent with findings from the Human Lung Cell Atlas, the expression of *Higd1b* is exclusive in PCs compared to other mural and vascular cells. The Violin plot of the *Higd1b* expression is highlighted to illustrate the left-skewed distribution of PCs.

(B) Density plots show the expression distribution of lung PC (*Cspg4, Pdgfrb,* and *Highd1b*) and SMC markers (*Acta2*, *Cnn1*, and *Tagln*) across all annotated cell types in the *Tabula Muris Senis* compendium. Subpanels highlight a magnified area from the UMAP, especially focusing on annotated PCs, further illustrating the distinct expression of *Higd1b* in PCs.

(C) Differential expression (DE) analysis utilizing the Wilcoxon rank-sum test was conducted to compare the expression of PC (*Cspg4, Pdgfrb,* and *Higd1b*) and SMC markers (*Acta2*, *Cnn1*, and *Tagln*) in annotated PCs, bronchial SMCs, and SMCs from the pulmonary artery against all other cell populations within the *Tabula Muris Senis* compendium. The analysis reveals that *Higd1b* is significantly upregulated in PCs relative to other cell types.

Appendix Figure S2

A

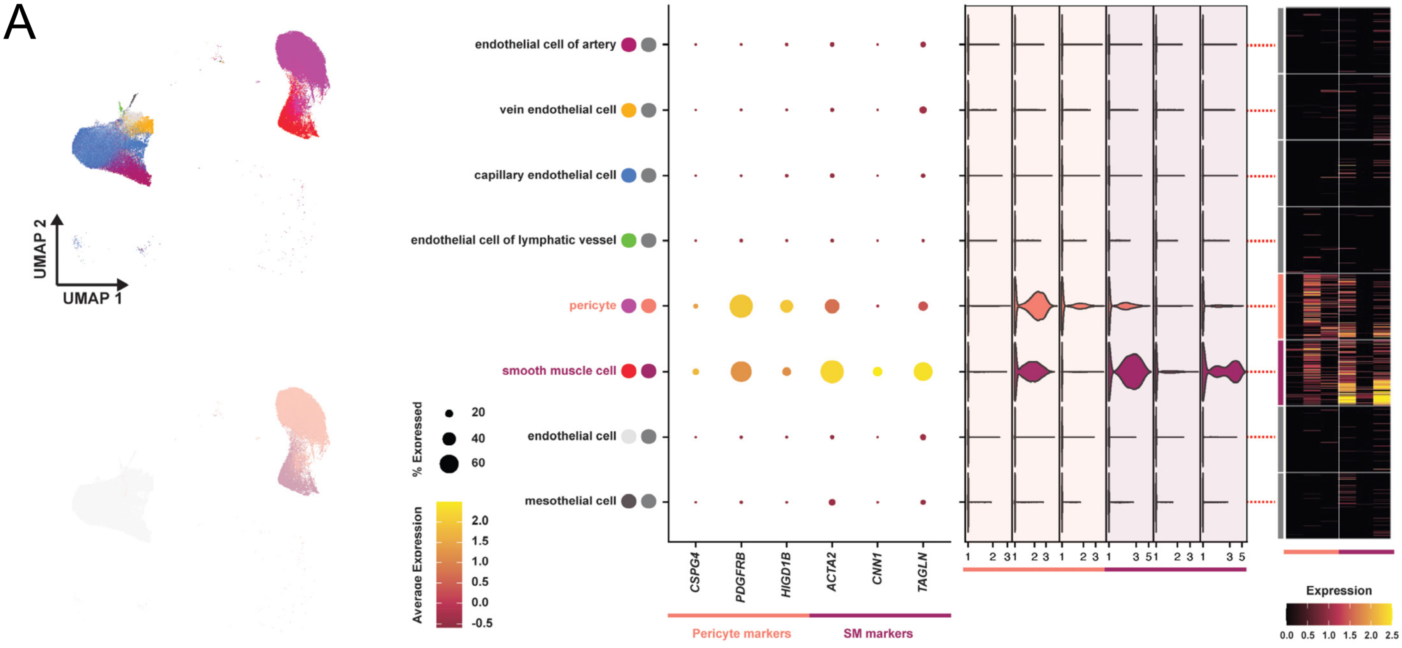

B

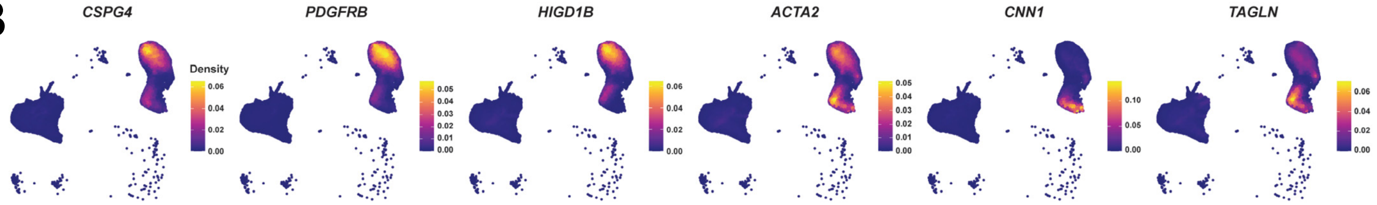

C

	pericyte vs Others				smooth muscle cell vs Others			
	Ave log2FC	padj	pct.1	pct.2	Ave log2FC	padj	pct.1	pct.2
CSPG4	2.331	padj < 1E-04	10.3%	2.6%	1.361	padj < 1E-04	16.1%	4.7%
PDGFRB	3.499	padj < 1E-04	76.8%	11.3%	0.373	padj < 1E-04	68.1%	34.6%
HIGD1B	2.836	padj < 1E-04	39.6%	6.9%	0.434	padj < 1E-04	23.7%	19.6%
ACTA2	0.755	padj < 1E-04	45.6%	17.8%	3.032	padj < 1E-04	76.8%	24.6%
CNN1	-1.172	padj < 1E-04	2.6%	4.2%	4.680	padj < 1E-04	27.0%	1.5%
TAGLN	-0.254	padj < 1E-04	26.8%	17.5%	3.955	padj < 1E-04	60.9%	17.6%

Human Heart (n = 195,395 cells), pericyte (n = 77,856), smooth muscle cell (n = 16,242), Others (n = 117,539): cells excluding pericyte, & Others (n = 179,153): cells excluding smooth muscle cell

Appendix Figure S2: *HIGD1B* is not exclusively expressed in human heart PCs.
(A) UMAP visualization of all cell populations within human heart tissue, based on the original UMAP coordinates and cell type annotations from the original data. Cell type annotations are color-coded as shown in the dot plot. PC and SMC distributions within lung tissues, using original UMAP coordinates and cell annotations from the original data are highlighted in the bottom left. In UMAP, Dot, Violin and Heatmap plots, PCs were highlighted in light orange and SMCs in dark red. The Dot and Violin plots on the right show the expression patterns of PC (*CSPG4, PDGFRB,* and *HIGD1B*) and SMC markers (*ACTA2*, *CNN1*, and *TAGLN*) across all annotated cell types.
(B) Density plots show the expression distribution of PC (*CSPG4, PDGFRB,* and *HIGD1B*) and SMC markers (*ACTA2*, *CNN1*, and *TAGLN*) across all annotated cell types from the human heart data.
(C) Differential expression (DE) analysis utilizing the Wilcoxon rank-sum test shows the expression of PC (*CSPG4, PDGFRB,* and *HIGD1B*) and SMC markers (*ACTA2*, *CNN1*, and *TAGLN*) in annotated PCs and SMCs against all other cell populations within the human heart data.

Appendix Figure S3

A

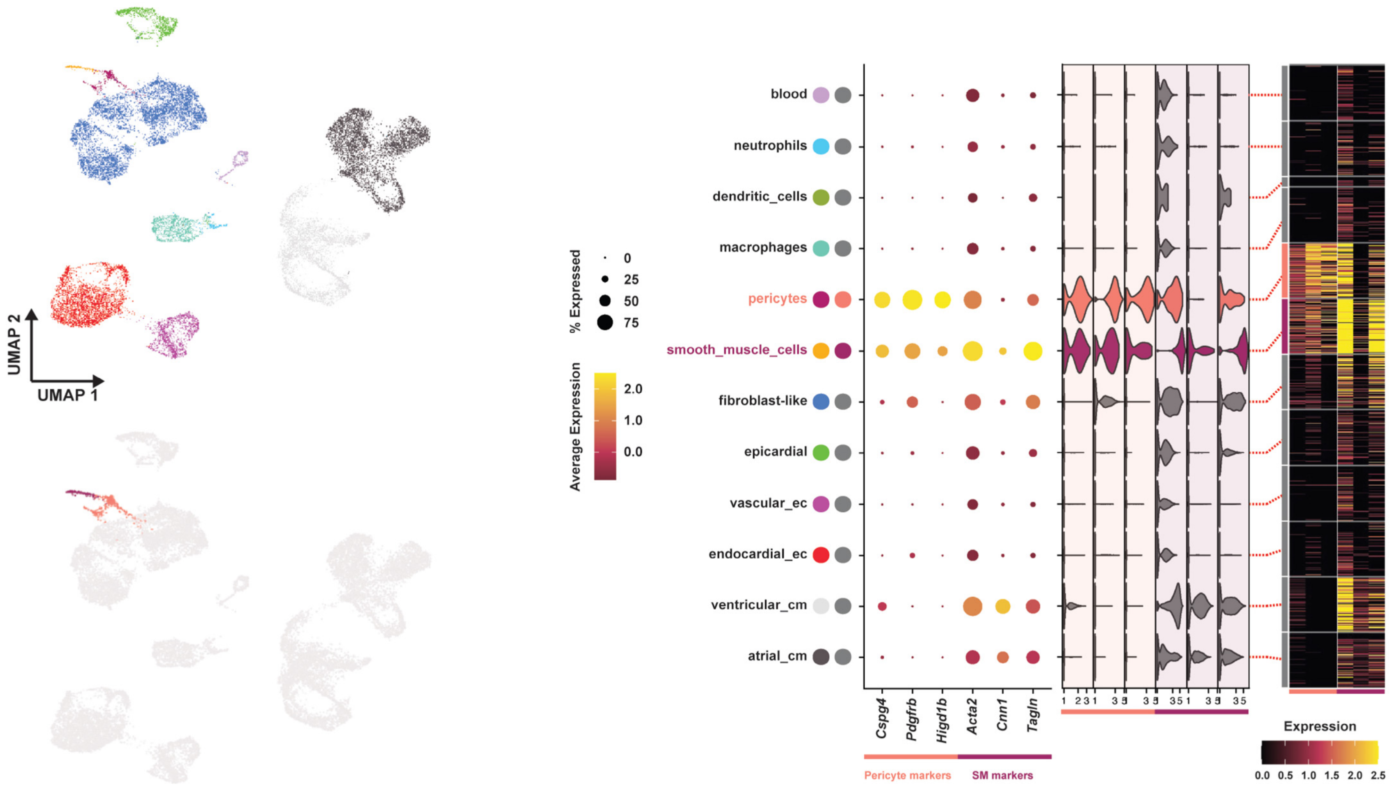

B

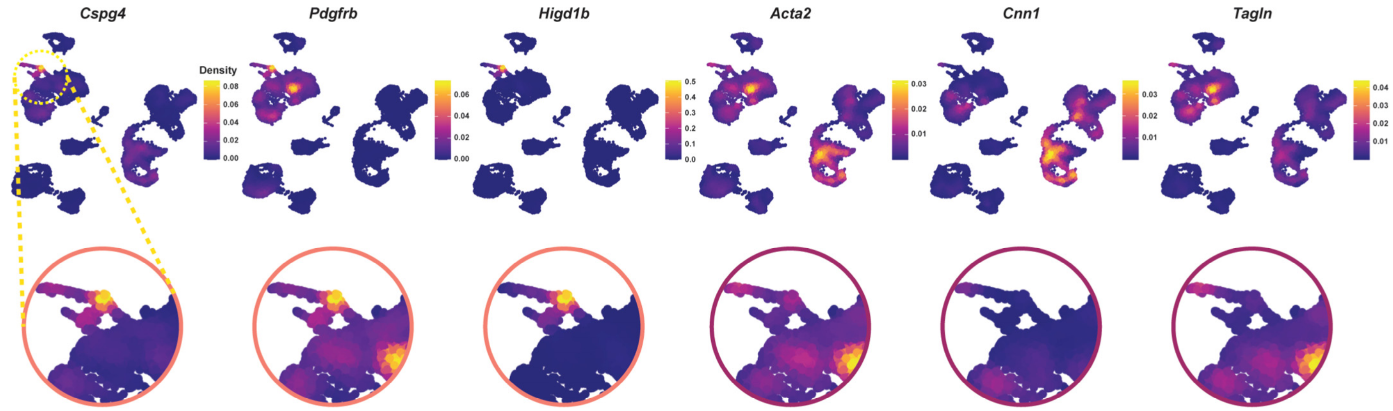

C

	pericyte vs Others				smooth muscle cell vs Others			
	Ave log2FC	padj	pct.1	pct.2	Ave log2FC	padj	pct.1	pct.2
Cspg4	4.125	padj < 1E-04	76.7%	13.3%	3.561	padj < 1E-04	61.5%	13.9%
Pdgfrb	4.101	padj < 1E-04	97.1%	18.3%	2.716	padj < 1E-04	75.8%	19.1%
Higd1b	8.183	padj < 1E-04	79.8%	0.8%	4.545	padj < 1E-04	44.1%	1.7%
Acta2	1.216	padj < 1E-04	87.0%	70.8%	3.879	padj < 1E-04	98.8%	70.9%
Cnn1	-2.119	padj < 1E-04	10.1%	32.5%	1.474	1	29.8%	32.1%
Tagln	0.477	1	52.0%	51.3%	4.192	padj < 1E-04	93.2%	51.1%

Mouse Heart (n = 25,436 cells), pericytes (n = 377), smooth muscle cells (n = 161), Others (n = 25,059): cells excluding pericytes, & Others (n = 25,275): cells excluding smooth muscle cells

Appendix Figure S3: *Higd1b* is not exclusively expressed in murine heart PCs.

(A) UMAP visualization of all cell populations within murine heart tissue, based on the original UMAP coordinates and cell type annotations from the murine heart data. Cell type annotations are color-coded as shown in the dot plot. PC and SMC distributions within lung tissues, using original UMAP coordinates and cell annotations from the murine heart data, are highlighted at the bottom left. In UMAP, Dot, Violin and Heatmap plots, PCs were highlighted in light orange and SMCs in dark red. The Dot and Violin plots on the right display the expression patterns of PC (*Cspg4, Pdgfrb,* and *Higd1b*) and SMC markers (*Acta2*, *Cnn1*, and *Tagln*) across all annotated cell types.

(B) Density plots show the expression of cardiac PC (*Cspg4, Pdgfrb,* and *Highd1b*) and SMC markers (*Acta2*, *Cnn1*, and *Tagln*) across all annotated cell types in the murine heart data. Subpanels focus on the magnified areas from the UMAP visualization, focusing on annotated PCs.

(C) Differential expression (DE) analysis utilizing the Wilcoxon rank-sum test shows the expression of PC (*Cspg4, Pdgfrb,* and *Higd1b*) and SMC markers (*Acta2*, *Cnn1*, and *Tagln*) in annotated PCs and SMCs compared to all other cell populations within the murine heart data.

Appendix Figure S4

Genes expressing in Pericytes above the criteria

Human Lung					Mouse Lung				
**Gene**	**Ave log2FC**	**padj**	**pct.1**	**pct.2**	**Gene**	**Ave log2FC**	**padj**	**pct.1**	**pct.2**
COX4I2	3.519	< 1E-04	92.3%	0.8%	***Higd1b***	10.160	< 1E-04	100%	0.8%
HIGD1B	3.239	< 1E-04	85.6%	0.5%	***Cstdc2***	10.148	< 1E-04	85.2%	0.1%
					Postn	10.099	< 1E-04	100%	0.6%
					Fam162b	9.766	< 1E-04	90.2%	0.2%
					Vtn	9.567	< 1E-04	90.2%	0.2%
					Notch3	9.186	< 1E-04	86.9%	0.2%
					Lipg	8.730	< 1E-04	90.2%	0.5%
					Vsnl1	8.589	< 1E-04	88.5%	0.5%
					Kcnk3	8.487	< 1E-04	91.8%	0.9%
					Tmem178	8.373	< 1E-04	85.2%	0.5%
					Art3	8.213	< 1E-04	75.4%	0.7%
					Ltbp2	8.133	< 1E-04	82.0%	0.6%
					Gap43	8.008	< 1E-04	80.3%	0.5%
					Pcdh18	7.902	< 1E-04	77.0%	0.6%

Human Heart					Mouse Heart				
**Gene**	**Ave log2FC**	**padj**	**pct.1**	**pct.2**	**Gene**	**Ave log2FC**	**padj**	**pct.1**	**pct.2**
No gene met the criteria					***Higd1b***	8.183	< 1E-04	79.8%	0.8%

***The criteria is set to include genes that have a log2FC >2 & padj < 0.05 & pct.1 > 75% & pct.2 < 1%**

Appendix Figure S4: Potential PC-specific cell markers are identified from differentially expressed genes in PCs across human and murine lung and heart scRNA-seq data.
Genes identified through differential expression analysis across the four scRNA-seq data, compared to PCs to all other cell types in each data, reveal that *HIGD1B* in human lung and *Higd1b* in murine lung and heart data demonstrated high expression levels in PCs. Genes presented have the following selection criteria; a log2FC >2, padj <0.05, pct.1 > 75%, and pct.2 <1%.

Appendix Figure S5

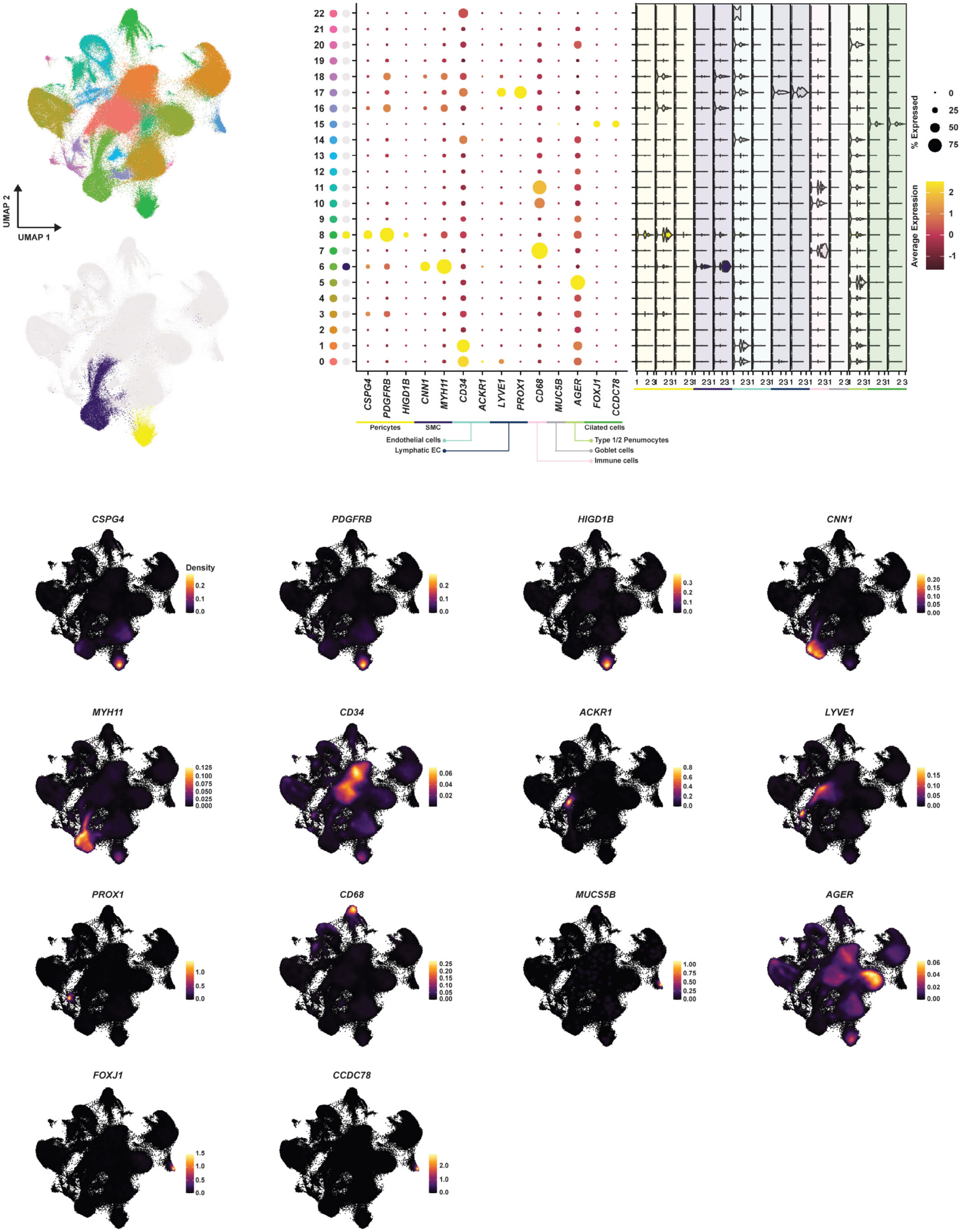

Appendix Figure S5: Cell types are annotated in spatial transcriptomic data utilizing known markers.

Unsupervised clustering utilizing the ‘FindCluster’ function identified 23 distinct clusters. Detailed cell type annotation processes are described in the Methods section. The UMAP visualization of the 23 clusters is shown on the top left. On the right, Dot and Violin plots show the expression of known cell-type markers across the cell clusters. The gene panel includes known cell type markers for PCs, SMCs, endothelial cells (ECs), lymphatic ECs, immune cells, goblet cells, type 1/2 penumocytes, and cilated cells. Cluster 8 (yellow) is enriched with PCs, based on the expression of key markers, such as *CSPG4, PDGFRB,* and *HIGD1B* and cluster 6 (dark blue) is enriched with SMCs, based on the expression of markers like *ACTA2, CNN1,* and *TAGLN*. At the bottom, density plots further illustrate the distribution of these cell-type markers, corroborating the findings from the dot and violin plots.

Appendix Figure S6

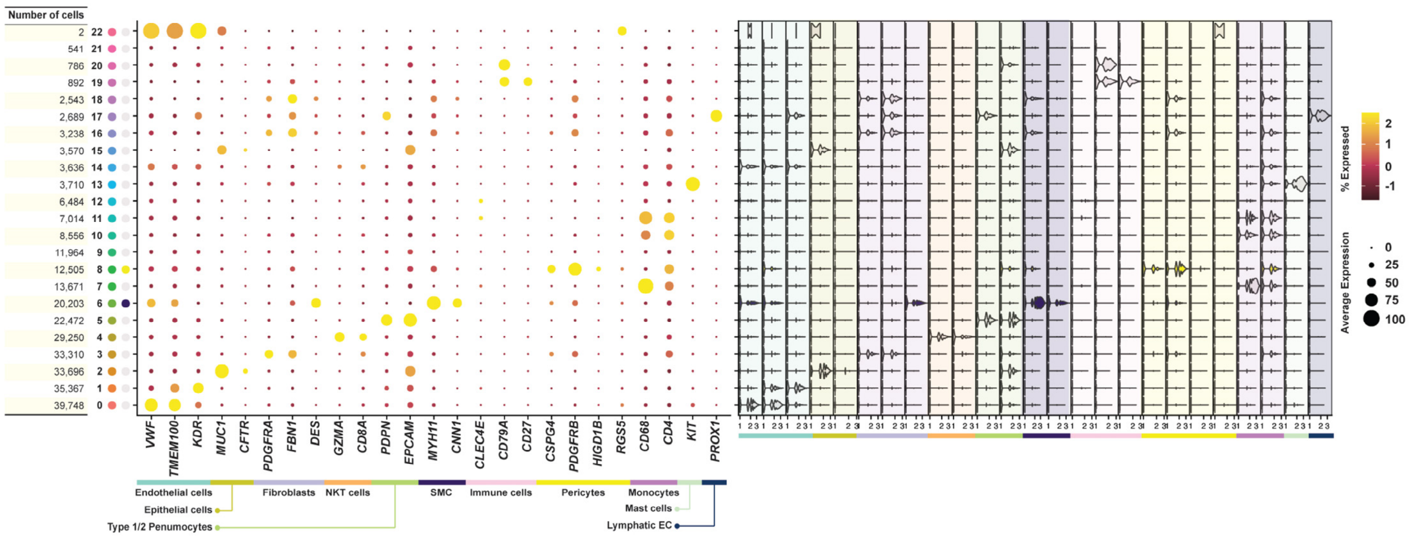

Clusters	Known Markers	Computational Annotation	DE gene Markers	Final
	Based on:			
22	Unknown	Hematopoietic stem cell	Unknown	Unknown
21	Unknown	Epithelial cell of alveolus of lung	Unknown	Unknown
20	Unknown	B cell	Immune cells-lymphoid cells	Immune cells
19	Unknown	Plasma cell	B cell	Immune cells
18	Unknown	Alveolar type 2 fibroblast cell	Stromal-FB	Fibroblasts
17	Lymphatic EC	Endothelial cell of lymphatic vessel	Lymphatic EC	Lymphatic EC
16	Unknown	Alveolar type 1 fibroblast cell	Myofibroblasts	Fibroblasts
15	Cilitated Cell	Ciliated columnar cell of tracheobronchial tree	Type 2 epithelial	Cilitated Cell
14	Unknown	Conventional dendritic cell	T-cell	Immune cells
13	Unknown	Mast cell	Mast cells	Mast Cells
12	Unknown	Alveolar macrophage	Neutrophil-monocytes	Immune cells
11	Immune Cell	Non-classical monocyte	Monocytes	Monocytes
10	Immune Cell	Lung macrophage	Monocytes	Immune cells
9	Unknown	Type II pneumocyte	Unknown	Unknown
8	Pericytes	Smooth muscle cell	Pericytes	Pericytes
7	Immune Cell	Alveolar macrophage	Myeloid cells	Immune cells
6	Smooth Muscle Cell	Smooth muscle cell	SMC-mural cells	Smooth muscle cell
5	Tyep 1/2 Pneumocytes	Type I pneumocyte	Unknown	Tyep 1/2 Pneumocytes
4	Unknown	T cell	NKT cells	NKT cells
3	Unknown	Alveolar type 1 fibroblast cell	Fibroblasts	Fibroblasts
2	Unknown	Type II pneumocyte	Epithelial Cells	Epithelial Cell
1	Endothelial Cell	Capillary endothelial cell	Endothelial Cell	Endothelial Cell
0	Endothelial Cell	Capillary endothelial cell	Endothelial Cell	Endothelial Cell

Computational Annotation was conducted using 'SingleR' utilizing HLCA as reference

Appendix Figure S6: Cell types are annotated in spatial transcriptomic data utilizing computational cell type annotation and differentially expressed (DE) genes.
Dot and violin plots show the expression patterns of selected differential expression genes between the clusters, which were utilized to annotate cell types. Utilizing the Human Lung Cell Atlas as a reference, 'singleR' was used to computationally annotate the cell types. For the final cell type annotation, we implemented a consensus-based approach. When the annotation approaches yielded similar, yet not identical results, we utilized a broader, more general category to ensure consistency across the data and eliminate minimal discrepancies. The table below summarizes each cell cluster and its final identification based on this analysis.

Appendix Figure S7

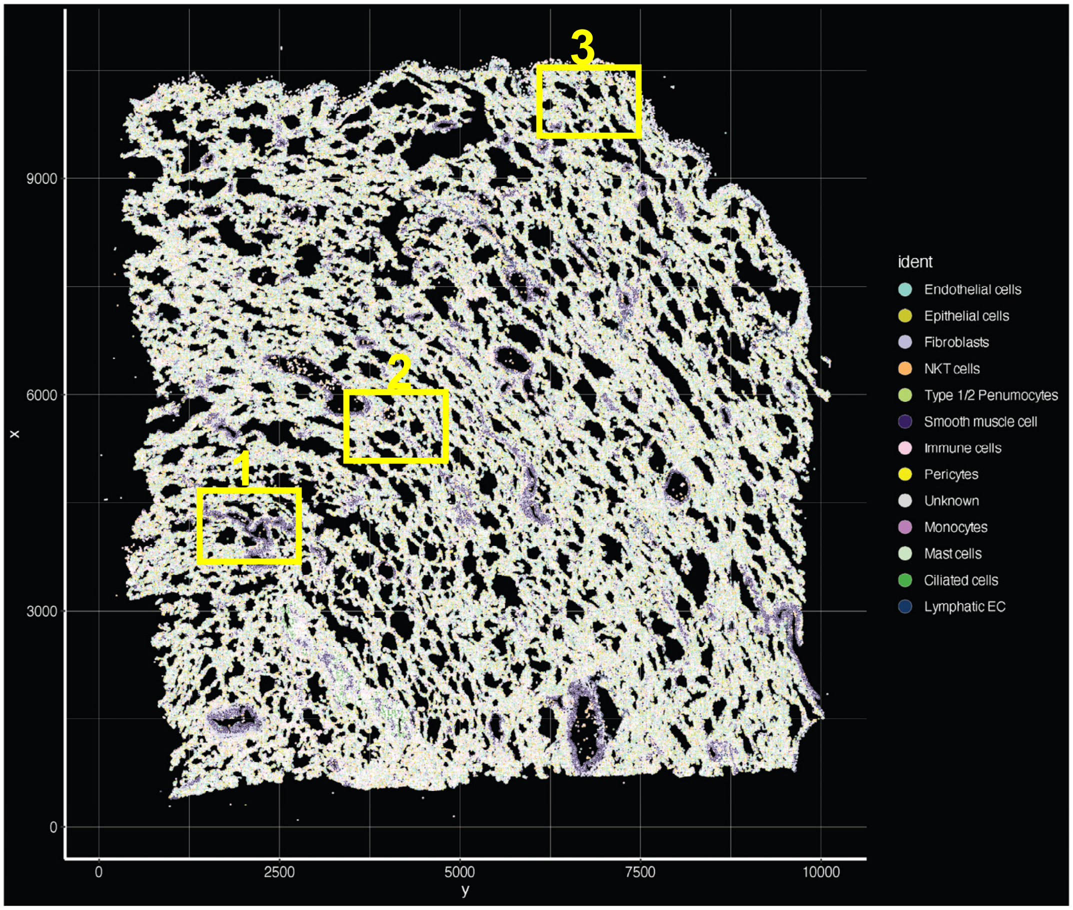

Area:	1	2	3
x:	3,750~4,550	5,200~6,000	9,700~10,500
y:	1,200~2,900	3,300~5,000	5,800~7,500

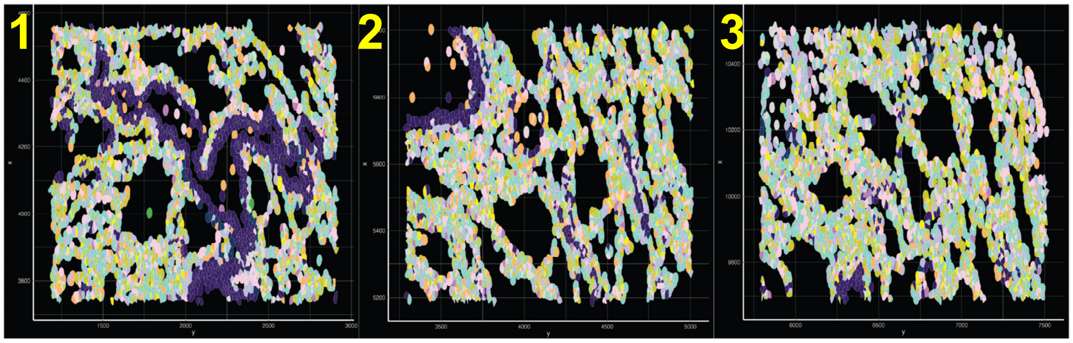

Appendix Figure S7: Spatial analysis of PC markers in non-diseased lung tissue.
The full lung tissue slide of the spatial transcriptomic data consists of 295,883 cells. The cell types, identified through previous analysis and annotations, have been mapped back to their respective locations within the tissue. The spatial distribution of these cell types is visualized in the full tissue slide, with each cell type represented by a distinct color, as indicated in the color-coded legend on the right side of the figure. Magnified areas for quantifications of the spatial transcriptomic map are found in yellow boxes and corresponding coordinates.

Appendix Figure S8

Higd1b is negative in arterial SMCs

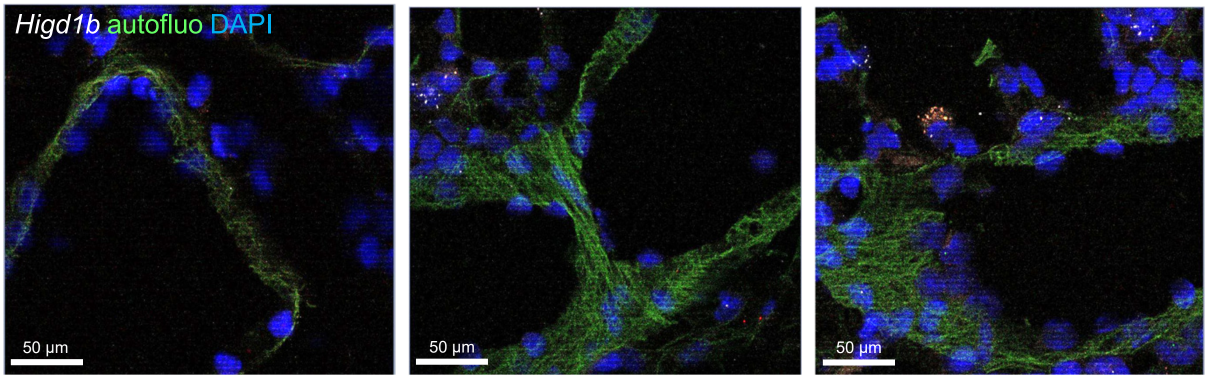

Appendix Figure S8: *Higd1b* mRNA expression is absent in arterial SMC layers. RNAscope shows the absence of *Higd1b* (white) expression in arterial SMC layers. Autofluorescence in green indicates a SMC layer. DAPI: blue. Scale bar: 50 µm.

Appendix Figure S9

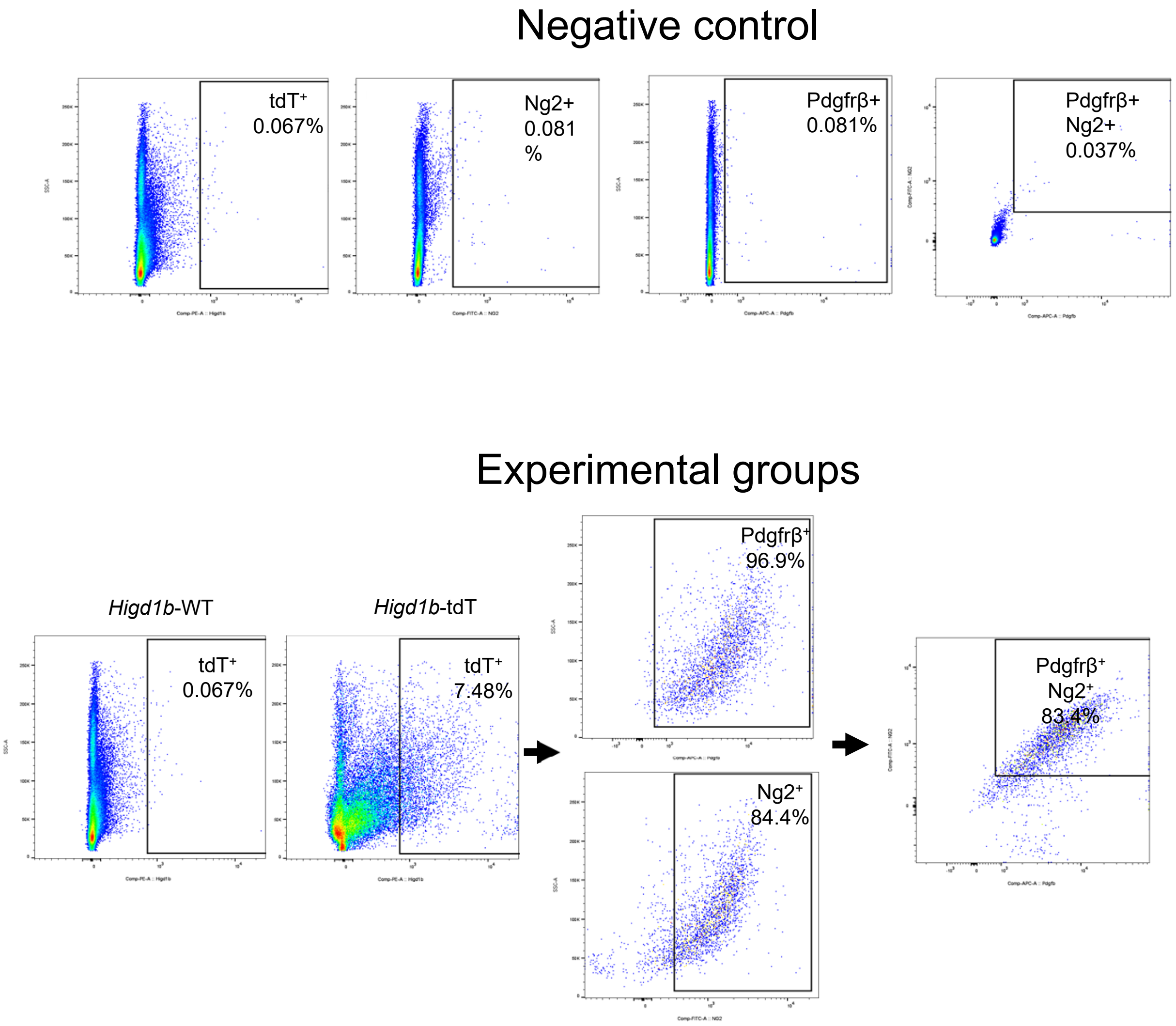

Appendix Figure S9: tdT+ cells from Higd1b-tdT lungs coexpress Pdgfrβ and Ng2 by FACS.

Flow plots from the tamoxifen-treated lungs of Higd1b-WT+/- and Higd1b-tdT+/- mice illustrate the gating and stepwise isolation of tdT+ cells, highlighting their expression of mural cell markers Pdgfrβ and Ng2. In the Higd1b-tdT+/- mice, tdT+ PCs showed high expression levels of both Pdgfrβ and Ng2. The analysis involved three animals for each condition, with FACS experiments being repeated three times. The same tdT-negative control was used across all experimental groups to establish gating for the tdT-positive population.

Appendix Figure S10

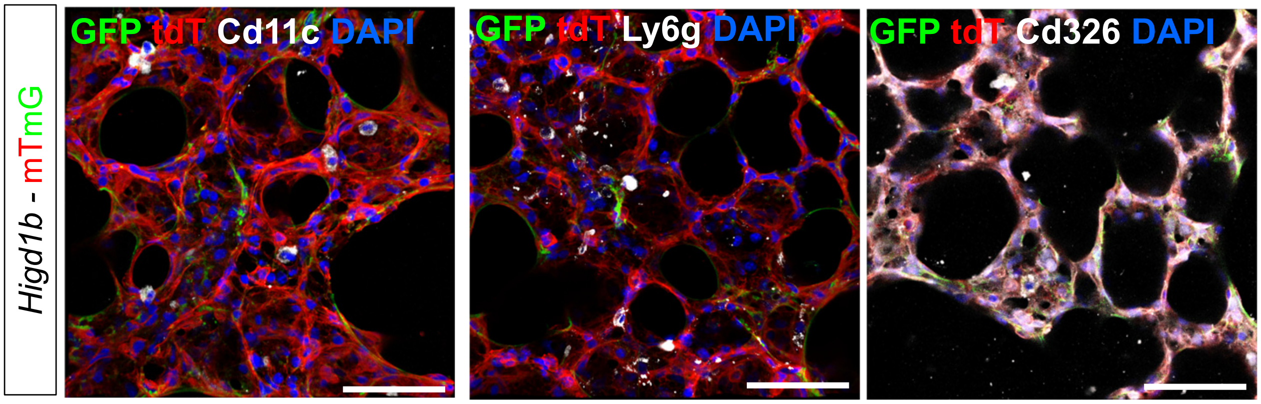

Appendix Figure S10: GFP+ cells from *Higd1b*-mTmG+/- mice do not express epithelial or immune cell markers.

PCLSs from *Higd1b*-mTmG+/- mice were stained for macrophages with Cd11c (white, left panel), neutrophils with Ly6g (middle panel), and epithelial cells (Cd326, right panel). DAPI: blue. Scale bar: 50 µm.

Appendix Figure S11

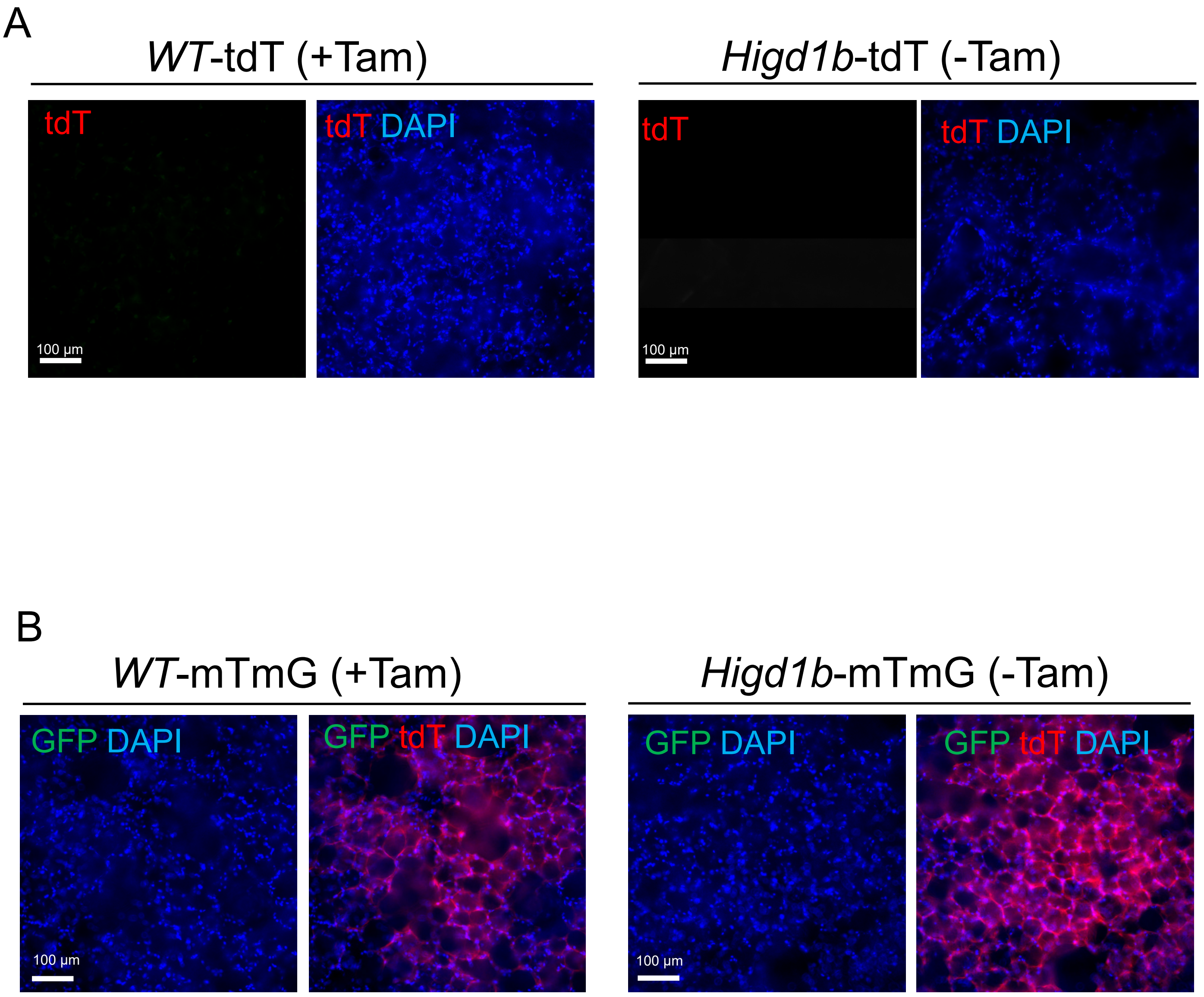

Appendix Figure S11: Tamoxifen is not injected into *Higd1b*-tdT+/- and *Higd1b*-mTmG+/- or tamoxifen injection on WT littermates.
(A) PCLSs from *WT-tdT+/-* mice after tamoxifen administration and *Higd1b-tdT+/-* mice without tamoxifen revealing an absence of tdT+ cells (red). DAPI: blue. Scale bar: 100 µm.
(B) PCLSs from *WT-mTmG+/-* mice with tamoxifen and *Higd1b-mTmG+/-* mice without tamoxifen showing no membrane GFP reporter (green) expression but the presence of membrane tdT color (red). tdT reporter: red. DAPI: blue. Scale bar: 100 µm.

Appendix Figure S12

A

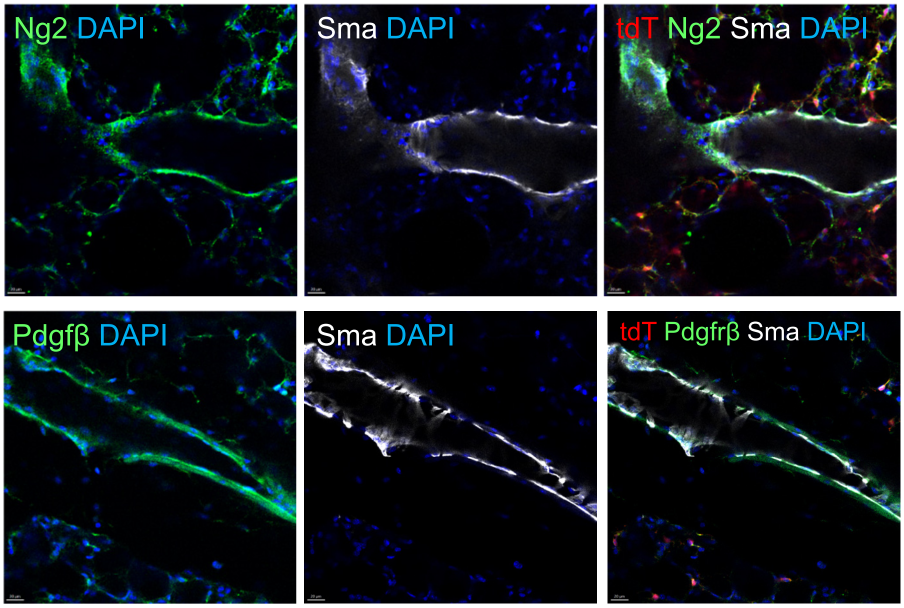

B

Cspg4-CreERTM*(Jax Strain #008538) :: *R26R-mTmG
Tamoxifen dose: 2mg/25 body weight

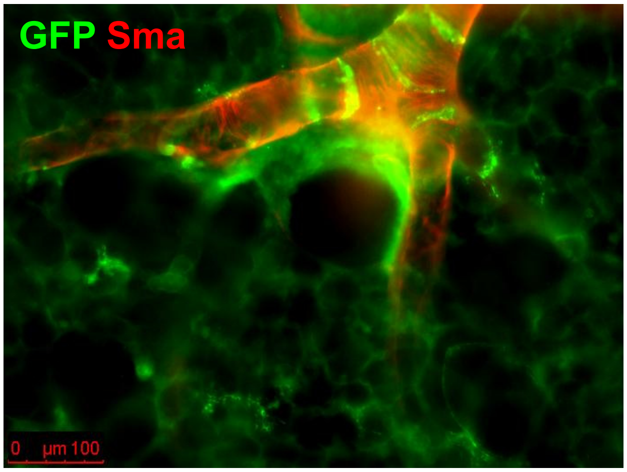

Pdgfrb-CreERT2*(Jax Strain #029684) :: *Ai14
No tamoxifen injected

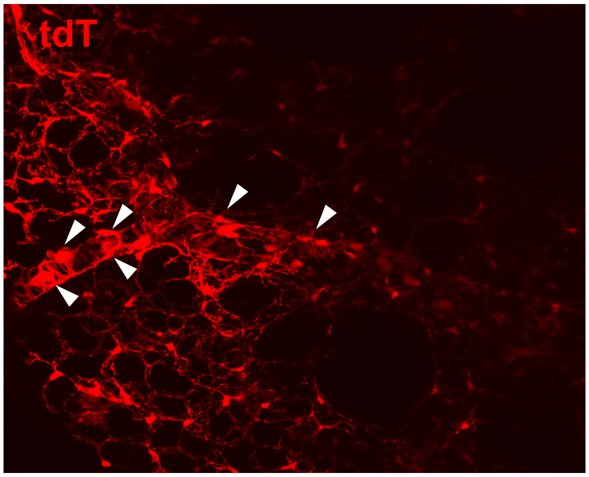

Appendix Figure S12: Pdgfrβ and Ng2 are non-specific markers for lung PCs.
(A) PCLSs from *Higd1b-tdT+/-* mice were stained for Ng2 (green, top panel) and Pdgfrβ (green, bottom panel), showing both antibodies overlapped with Sma (white). Scale bar: 20 µm.
(B) PCLSs from *Cspg4-mTmG+/-* mice injected with tamoxifen exhibited nonspecific labeling of the endogenous reporter (GFP, green; white arrowhead) on Sma+ vessels (top panel). Coexpression of GFP with Sma (red) was highlighted with white arrows. Scale bar: 100 µm. PCLSs from *Pdgfrb-tdT+/-* mice without tamoxifen showed tdT-labeled PCs and SMCs(bottom panel), with white arrowheads representing SMCs. Scale bar: 100 µm.

Appendix Figure S13

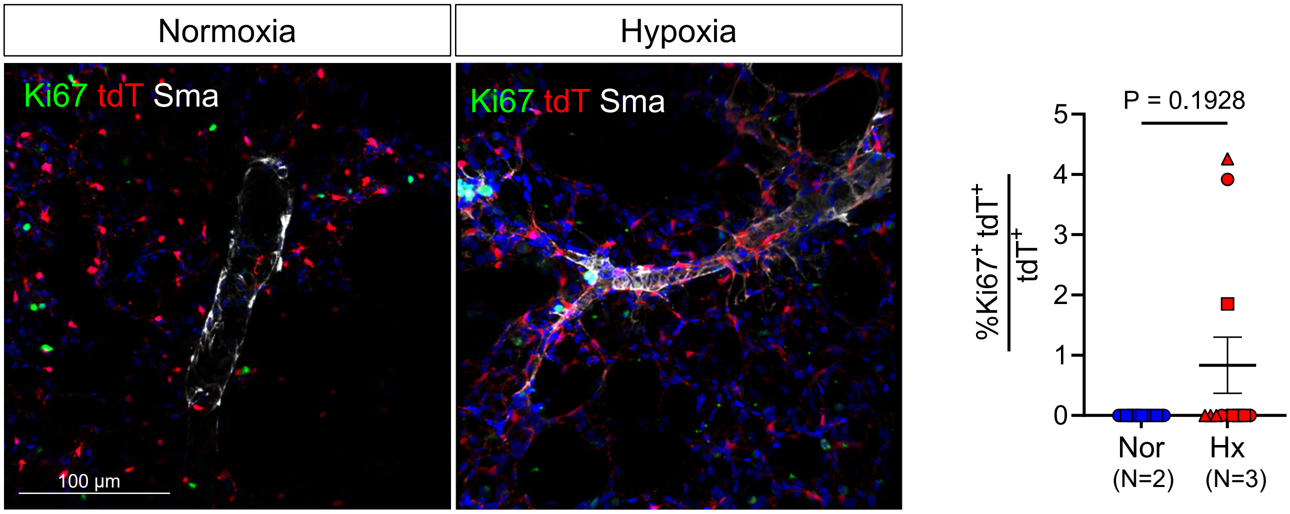

Appendix Figure S13: tdT+ PCs do not co-express Ki67 in response to chronic Hx. Representative images for Ki67 (green) staining demonstrate tdT+ cells (red) from the lungs of *Higd1b-tdT+/-* mice in normoxic and after 3 wks pf Hx. Quantification of Ki67+ tdT+ cells from normoxia (N=2) and 3wk Hx (N=3) mice. Sma (white), DAPI (blue). Scale bar: 100 µm. Each symbol represents each lung. Each dot represents the percentage of Ki67+ tdT labeled cells compared to the total number of tdT+ PCs in one image. Data is presented as mean ± SEM. Statistical analysis was performed using an unpaired t-test.

Appendix Figure S14

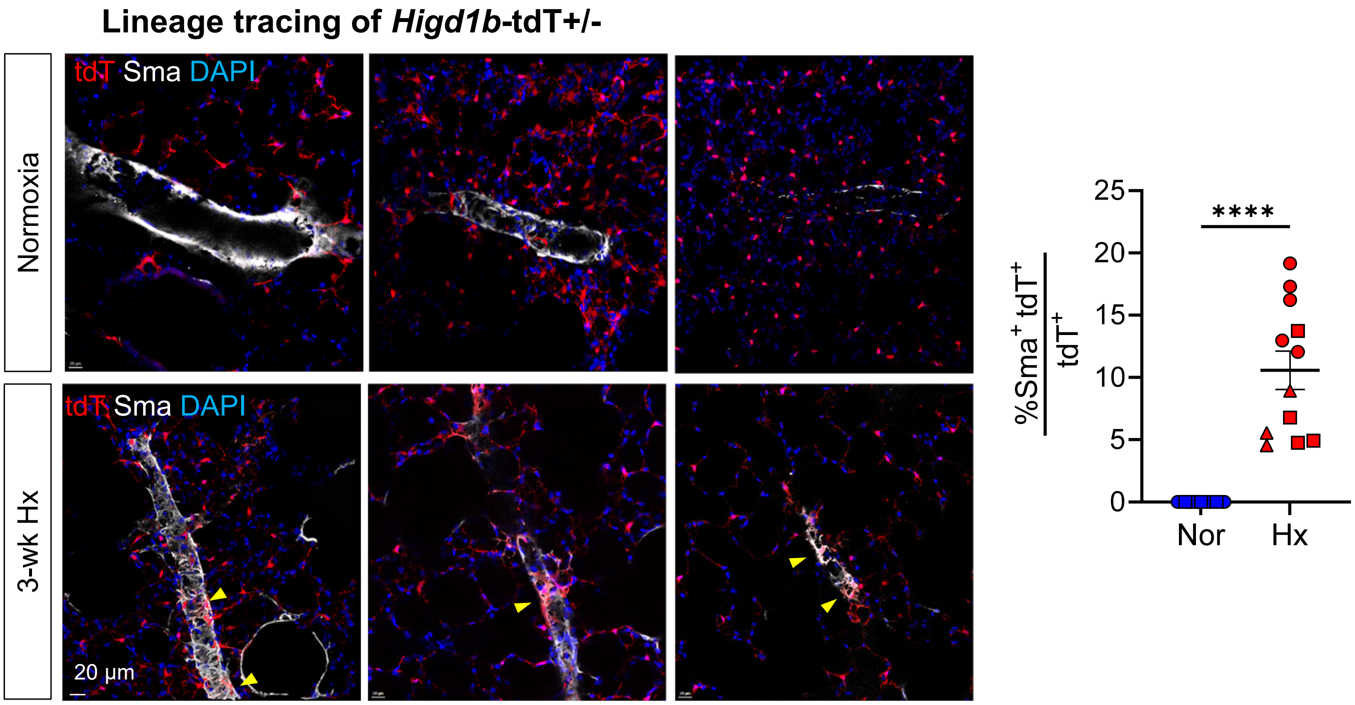

Appendix Figure S14: tdT+ cells accumulate in muscularized distal arterioles after 3wk Hx.

tdT+ PCs in normoxia from *Higd1b-tdT+/-* mice show Sma-negative tdT+ PCs (red) located in the parenchymal region and distal arterioles. After 3 wks of Hx, accumulation of tdT+ PCs (red) in muscularized distal arterioles were co-stained with SMC markers Sma (white). DAPI: blue. Scale bar: 20 µm. Each symbol represents each lung harvested from normoxia (N=2) and 3wk Hx (N=3). The number of Sma+ tdT+ cells was quantified relative to the total number of DAPI+ cells, with statistical significance determined by ****$P<0.0001$ unpaired t-test.

Appendix Figure S15

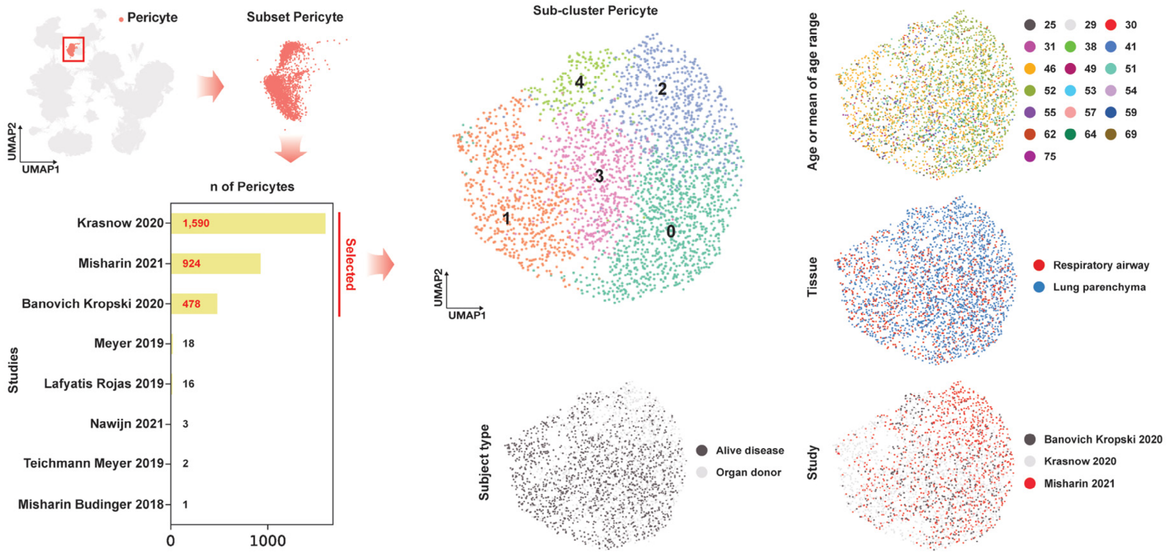

Number of Pericytes for each

Tissue	Sub-0	Sub-1	Sub-2	Sub-3	Sub-4	Total
● (red)	256	239	102	158	26	**1,590**
● (blue)	747	389	508	418	149	**1,402**
**Total**	**1,003**	**628**	**610**	**576**	**175**	**2,992**

Subject type	Sub-0	Sub-1	Sub-2	Sub-3	Sub-4	Total
● (dark grey)	546	450	188	323	83	**1,590**
● (light grey)	457	178	422	253	92	**1,402**
**Total**	**1,003**	**628**	**610**	**576**	**175**	**2,992**

Study	Sub-0	Sub-1	Sub-2	Sub-3	Sub-4	Total
● (dark grey)	175	62	77	113	51	**478**
● (light grey)	546	450	188	323	83	**1,590**
● (red)	282	116	345	140	41	**924**
**Total**	**1,003**	**628**	**610**	**576**	**175**	**2,992**

Donor ID

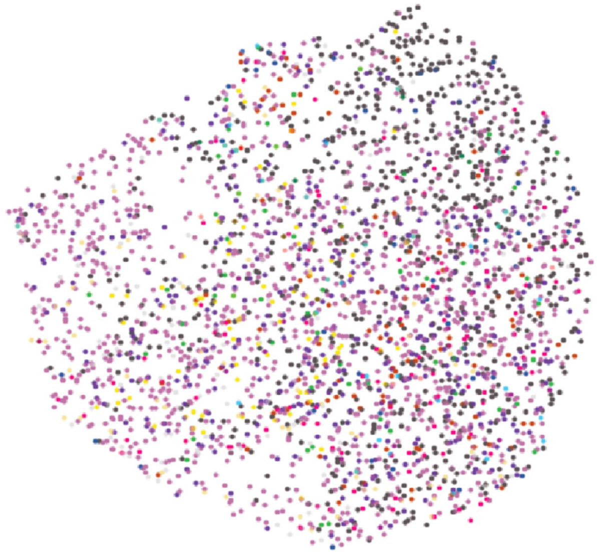

Donor ID color	Sub-0	Sub-1	Sub-2	Sub-3	Sub-4	Total
● (purple)	153	71	49	103	22	**398**
● (mauve)	368	354	138	210	59	**1,129**
● (pale yellow)	25	25	1	10	2	**63**
● (pink)	1	0	2	0	0	**3**
● (magenta)	59	16	20	15	5	**115**
● (green)	21	6	5	17	6	**55**
● (yellow)	3	26	2	41	3	**75**
● (violet)	10	1	5	5	1	**22**
● (brown)	0	0	1	3	0	**4**
● (dark green)	0	0	2	1	1	**4**
● (red-orange)	53	7	13	15	17	**105**
● (dark blue)	13	3	16	5	6	**43**
● (light pink)	2	0	2	1	1	**6**
● (purple)	0	0	0	0	1	**1**
● (lavender)	0	0	0	0	1	**1**
● (light blue)	11	0	2	2	2	**17**
● (olive)	0	0	0	2	0	**2**
● (teal)	1	1	6	5	2	**15**
● (crimson)	0	1	0	0	0	**1**
● (orange)	1	0	0	0	1	**2**
● (blue)	0	0	0	0	1	**1**
● (light green)	0	1	1	1	1	**4**
● (orchid)	0	0	0	0	1	**1**
● (red)	0	0	0	0	1	**1**
● (light grey)	16	53	11	8	5	**93**
● (dark grey)	266	63	334	132	36	**831**
**Total**	**1,003**	**628**	**610**	**576**	**175**	**2,992**

Appendix Figure S15: Sub-clustering of PCs was conducted from HLCA.
The PC cluster was sub-clustered from the HLCA (core). To mitigate the effects of potential confounders, we first excluded data from ‘Studies’ with fewer than 400 PCs, resulting in a final data of 2,992 PCs. We then employed Harmony to adjust for variables including ‘data’, ‘assay’, ‘tissue sampling method’, ‘sequencing platform’, ‘development stage’, ‘tissue’, ‘subject type’, ‘study’, ‘lung condition’, ‘sex’, ‘self-reported ethnicity’, and ‘age or mean of age range’. The cell composition across variables such as ‘Age or mean of age range’, ‘Tissue’, ‘Subject type’, ‘Study’, and ‘Donor ID’ did not reveal any significant batch effect enrichment in specific clusters.

Appendix Figure S16

GSEA

Pathways enriched (FDR q-val < 0.1) in Pericytes of cluster 0 compared to cluster 2

HALLMARK pathways	FDR q-val
REACTIVE_OXYGEN_SPECIES_PATHWAY	< 1E-05
OXIDATIVE_PHOSPHORYLATION	< 1E-05
FATTY_ACID_METABOLISM	< 1E-05
MYC_TARGETS_V1	< 1E-05
INTERFERON_ALPHA_RESPONSE	4E-04
INTERFERON_GAMMA_RESPONSE	5E-04
DNA_REPAIR	5E-04
ADIPOGENESIS	0.001
GLYCOLYSIS	0.005
MTORC1_SIGNALING	0.01
UNFOLDED_PROTEIN_RESPONSE	0.01
XENOBIOTIC_METABOLISM	0.01
PEROXISOME	0.01
ALLOGRAFT_REJECTION	0.03
HYPOXIA	0.05
UV_RESPONSE_UP	0.06
P53_PATHWAY	0.09

KEGG pathways	FDR q-val
RIBOSOME	< 1E-05
ANTIGEN_PROCESSING_AND_PRESENTATION	< 1E-05
PROTEASOME	< 1E-05
OXIDATIVE_PHOSPHORYLATION	< 1E-05
PARKINSONS_DISEASE	< 1E-05
HUNTINGTONS_DISEASE	< 1E-05
ALZHEIMERS_DISEASE	< 1E-05
SYSTEMIC_LUPUS_ERYTHEMATOSUS	1E-05
GLYCOLYSIS_GLUCONEOGENESIS	1E-04
CITRATE_CYCLE_TCA_CYCLE	2E-04
PYRUVATE_METABOLISM	0.002
PROTEIN_EXPORT	0.002
PYRIMIDINE_METABOLISM	0.003
COMPLEMENT_AND_COAGULATION_CASCADES	0.01
CARDIAC_MUSCLE_CONTRACTION	0.04
LYSOSOME	0.05
LEISHMANIA_INFECTION	0.05
SPLICEOSOME	0.05
CELL_ADHESION_MOLECULES_CAMS	0.06
PRION_DISEASES	0.06
PURINE_METABOLISM	0.07
VIRAL_MYOCARDITIS	0.07

Pathways enriched (FDR q-val < 0.1) in Pericytes of cluster 2 compared to cluster 0

HALLMARK pathways	FDR q-val
MITOTIC_SPINDLE	< 1E-05
G2M_CHECKPOINT	0.006
UV_RESPONSE_DN	0.008
TGF_BETA_SIGNALING	0.08
ESTROGEN_RESPONSE_EARLY	0.09

KEGG pathways	FDR q-val
ECM_RECEPTOR_INTERACTION	4E-04
PATHWAYS_IN_CANCER	6E-04
ARRHYTHMOGENIC_RIGHT_VENTRICULAR _CARDIOMYOPATHY_ARVC	6E-04
FOCAL_ADHESION	8E-04
SMALL_CELL_LUNG_CANCER	9E-04
DILATED_CARDIOMYOPATHY	0.001
HYPERTROPHIC_CARDIOMYOPATHY_HCM	0.001
PANCREATIC_CANCER	0.001
NON_SMALL_CELL_LUNG_CANCER	0.002
TGF_BETA_SIGNALING_PATHWAY	0.003
GLIOMA	0.003
ENDOMETRIAL_CANCER	0.003
WNT_SIGNALING_PATHWAY	0.003
MELANOMA	0.003
ERBB_SIGNALING_PATHWAY	0.003
MELANOGENESIS	0.003
NOTCH_SIGNALING_PATHWAY	0.003
CHRONIC_MYELOID_LEUKEMIA	0.008
VASCULAR_SMOOTH_MUSCLE_CONTRACTION	0.008
T_CELL_RECEPTOR_SIGNALING_PATHWAY	0.008
AXON_GUIDANCE	0.008
GNRH_SIGNALING_PATHWAY	0.009
LONG_TERM_DEPRESSION	0.01
ACUTE_MYELOID_LEUKEMIA	0.01
PROSTATE_CANCER	0.01
PHOSPHATIDYLINOSITOL_SIGNALING_SYSTEM	0.01
BLADDER_CANCER	0.01
VEGF_SIGNALING_PATHWAY	0.01
MTOR_SIGNALING_PATHWAY	0.01
MAPK_SIGNALING_PATHWAY	0.01
JAK_STAT_SIGNALING_PATHWAY	0.01
FC_EPSILON_RI_SIGNALING_PATHWAY	0.01
CHEMOKINE_SIGNALING_PATHWAY	0.01
CYTOKINE_CYTOKINE_RECEPTOR_INTERACTION	0.01
ADHERENS_JUNCTION	0.01
COLORECTAL_CANCER	0.01
RENAL_CELL_CARCINOMA	0.01
B_CELL_RECEPTOR_SIGNALING_PATHWAY	0.01
NEUROTROPHIN_SIGNALING_PATHWAY	0.02
REGULATION_OF_ACTIN_CYTOSKELETON	0.02
CALCIUM_SIGNALING_PATHWAY	0.02
LONG_TERM_POTENTIATION	0.03
INOSITOL_PHOSPHATE_METABOLISM	0.04
GAP_JUNCTION	0.07
INSULIN_SIGNALING_PATHWAY	0.08

Appendix Figure S16: Gene Set Enrichment Analysis in PC sub-cluster 0 vs 2. Differential expression (DE) analysis utilizing the Wilcoxon rank-sum test was conducted between subcluster 0 (highest *HIGD1B* expression among the subclusters) and subcluster 2 (lowest *HIGD1B* expression among the subclusters). The table shows the full result of Gene Set Enrichment Analysis (GSEA) of HallMark and KEGG comparing PC sub-cluster 0 and 2 with an FDR below 0.1.

Appendix Figure S17

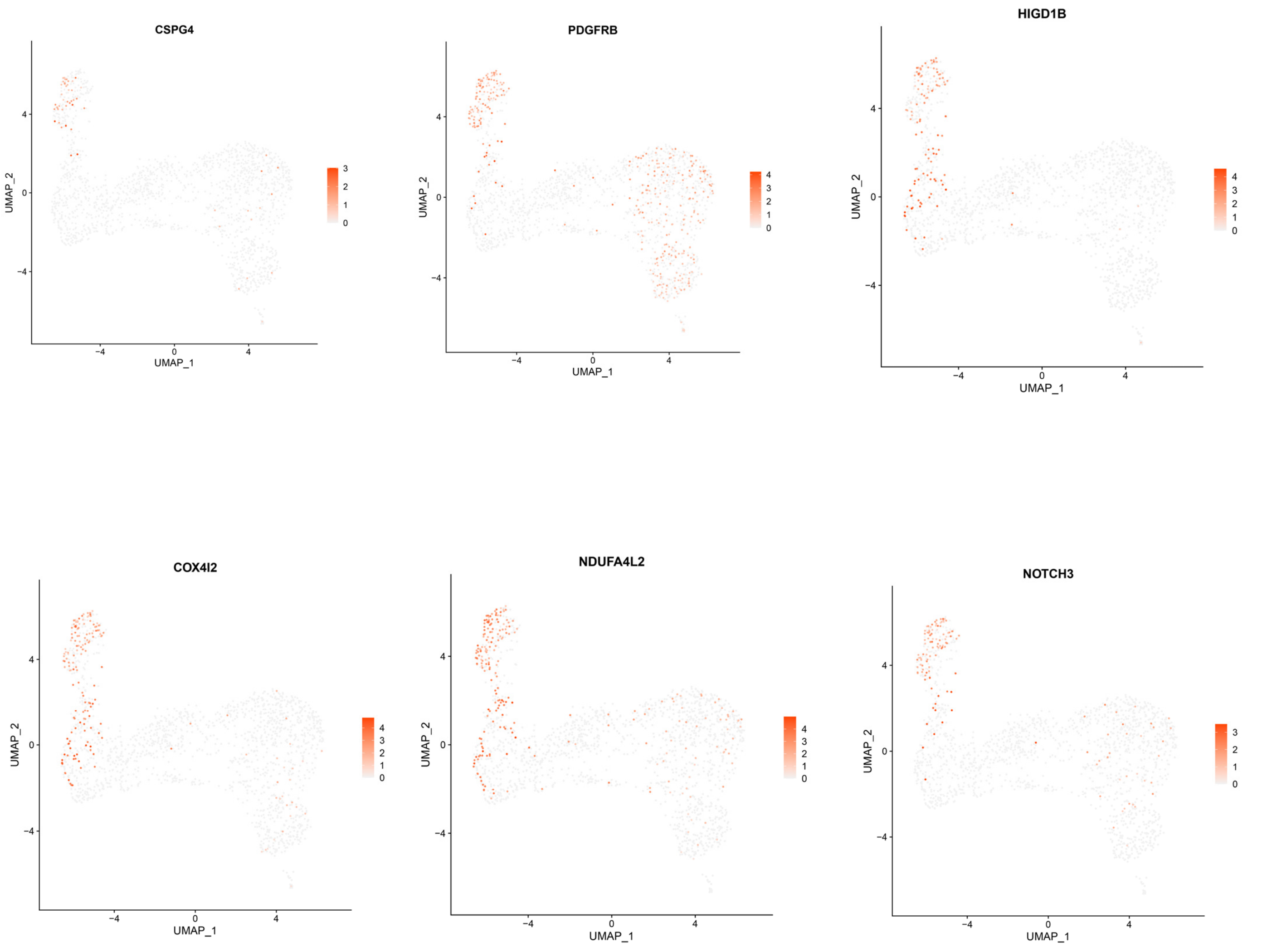

Appendix Figure S17: Mural cell cluster 5 and 6 feature plots from IPAH and control scRNA-seq data.
UMAP plots in mural sub-clusters 5 and 6 from IPAH and control scRNA-seq data show the relative expression of markers *CSPG4, PDGFRB, HIGD1B, COX412, NDUFA4L2,* and *NOTCH3*.

Appendix Figure S18

A

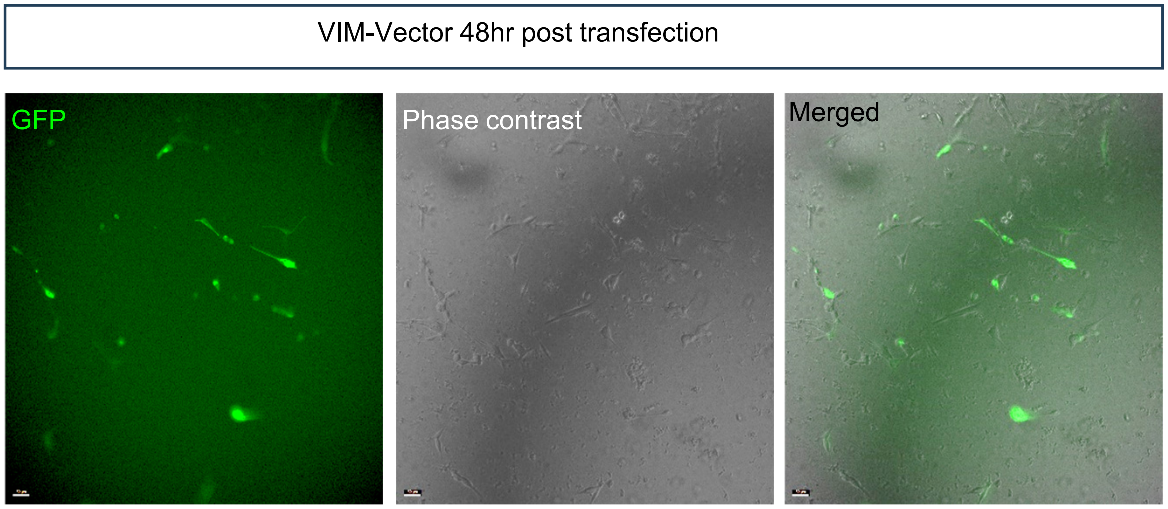

B

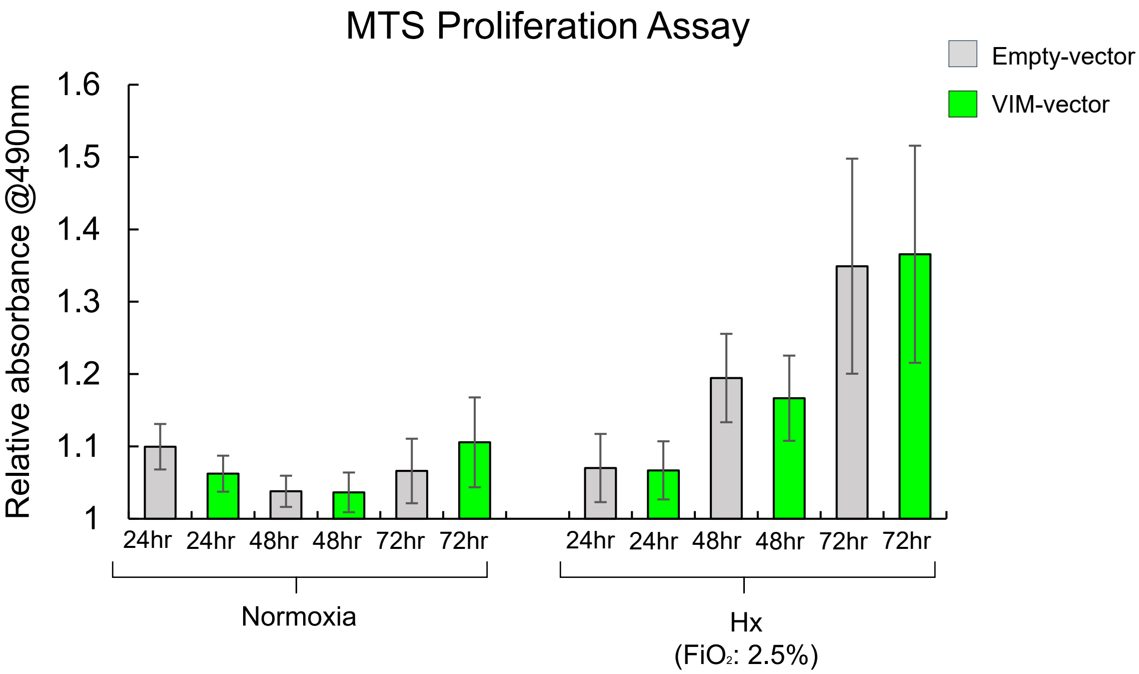

Appendix Figure S18: Overexpression of VIM in PCs does not enhance proliferation. (A) PCs transfected with VIM-vector expressed GFP after 48hr. (B) The MTS proliferation assay demonstrated no proliferation when measuring the relative 490nm absorbance of PCs in response to VIM overexpression (green) under normoxic and hypoxic conditions (2.5% FiO_2) over time. Controls were treated with an empty vector. Each dot represents quantifications from three biological replicates with three repeated experiments (N=3). Statistical analysis was performed with an unpaired t-test between VIM-vector vs empty-vector at the same time point under the same condition. Data presented as mean± standard error.
