# Supplementary figures and images for: Specialized pericyte subtypes in the pulmonary capillaries
Source: EMBO J. 2025 Jan 13;44(4):1074–106. doi: 10.1038/s44318-024-00349-1 (PMC11833098; doi:10.1038/s44318-024-00349-1)

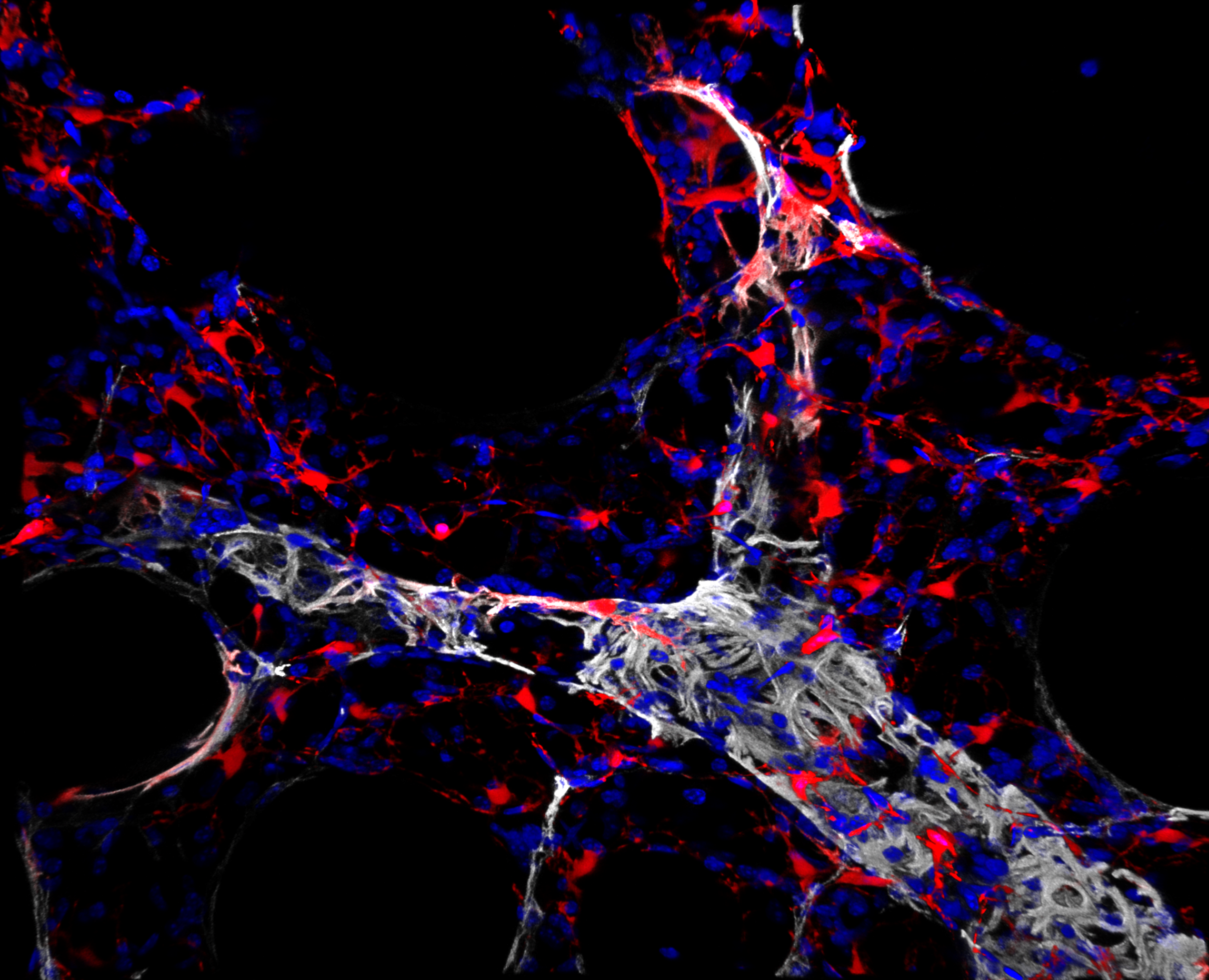

Supplement: Supplementary file 5 — Source data Fig. 4 [file 44318_2024_349_MOESM5_ESM.zip › 4A/artery1.png]

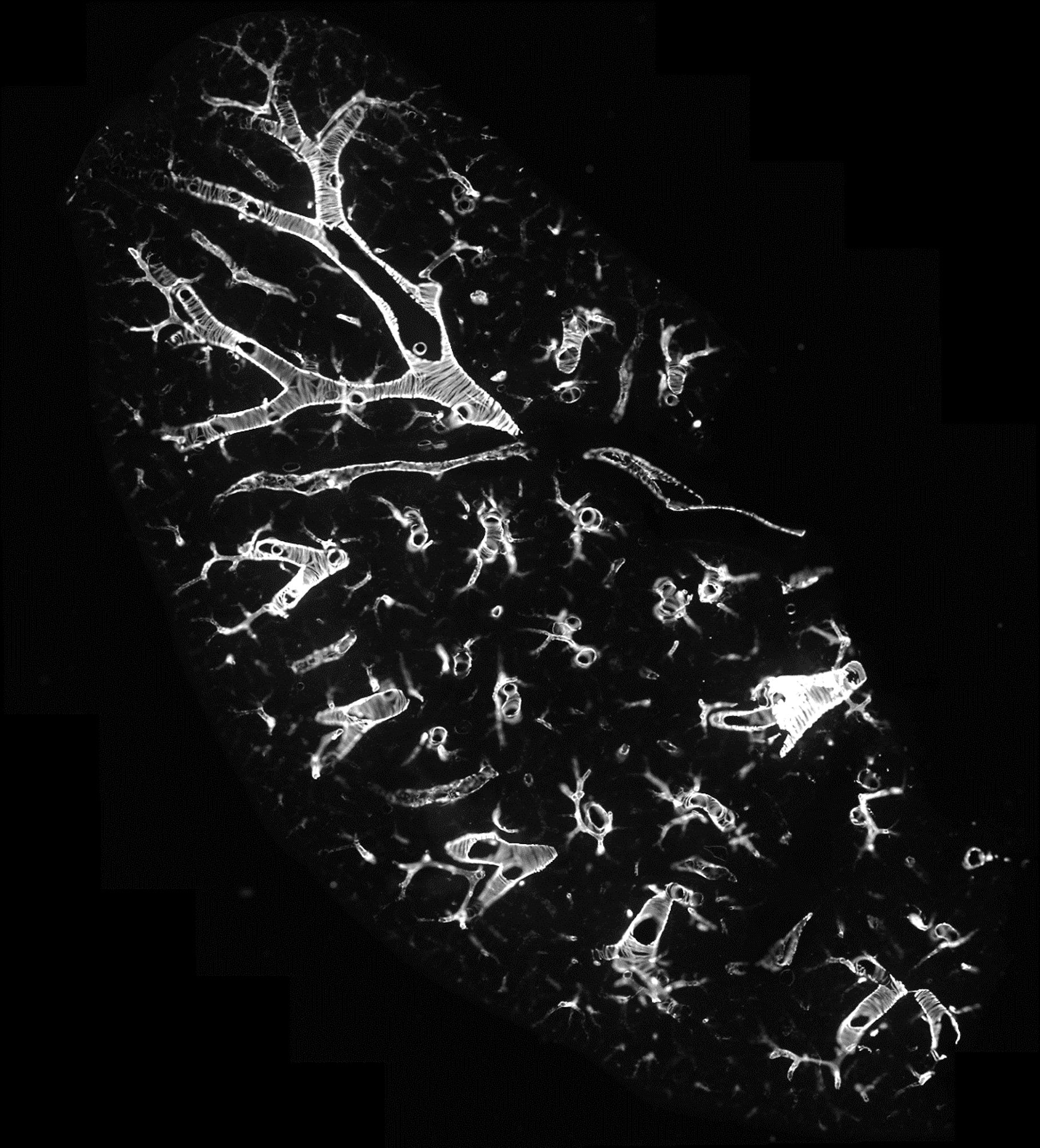

Supplement: Supplementary file 5 — Source data Fig. 4 [file 44318_2024_349_MOESM5_ESM.zip › 4A/hx-all.png]

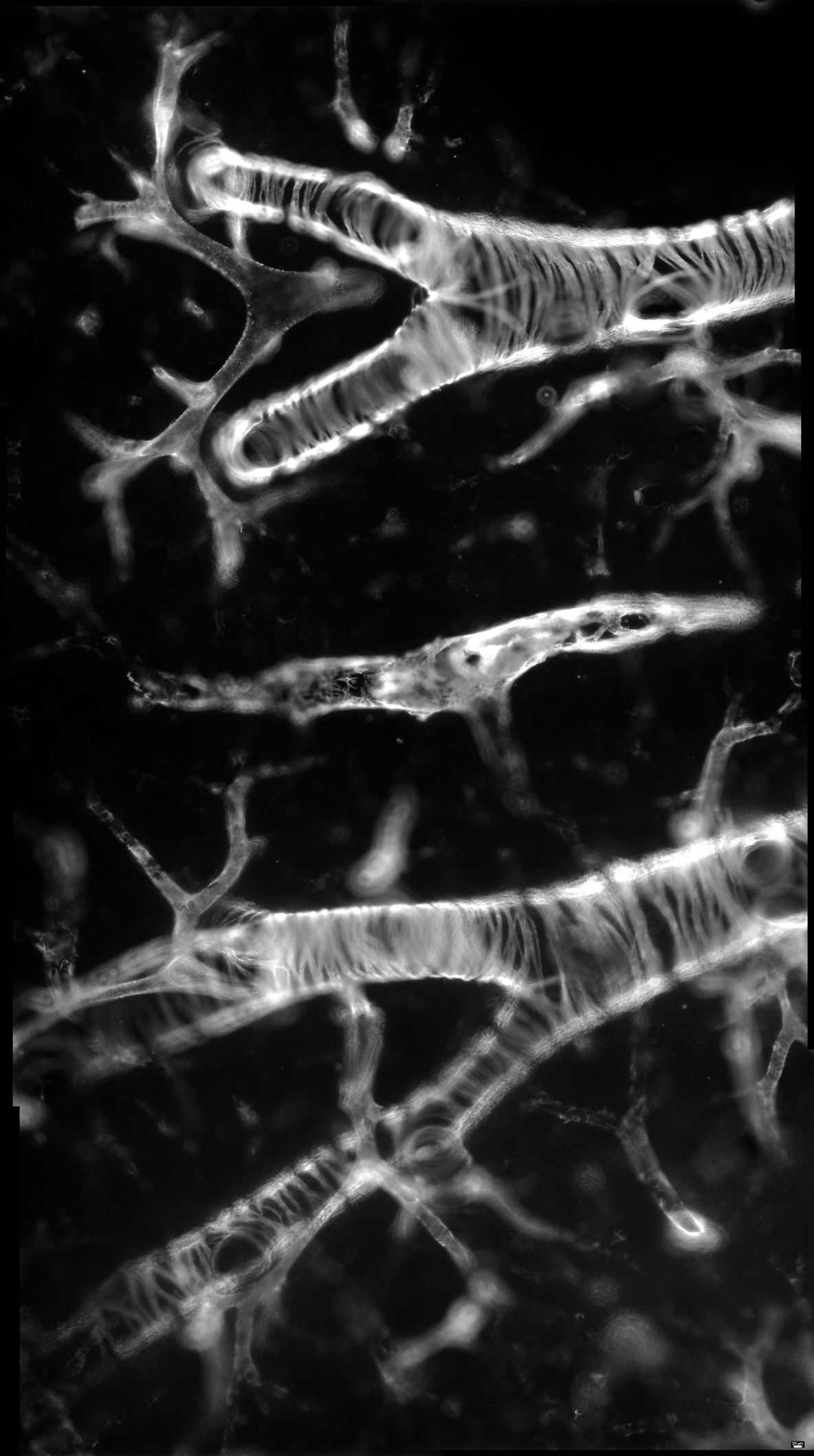

Supplement: Supplementary file 5 — Source data Fig. 4 [file 44318_2024_349_MOESM5_ESM.zip › 4A/norm-all.png]

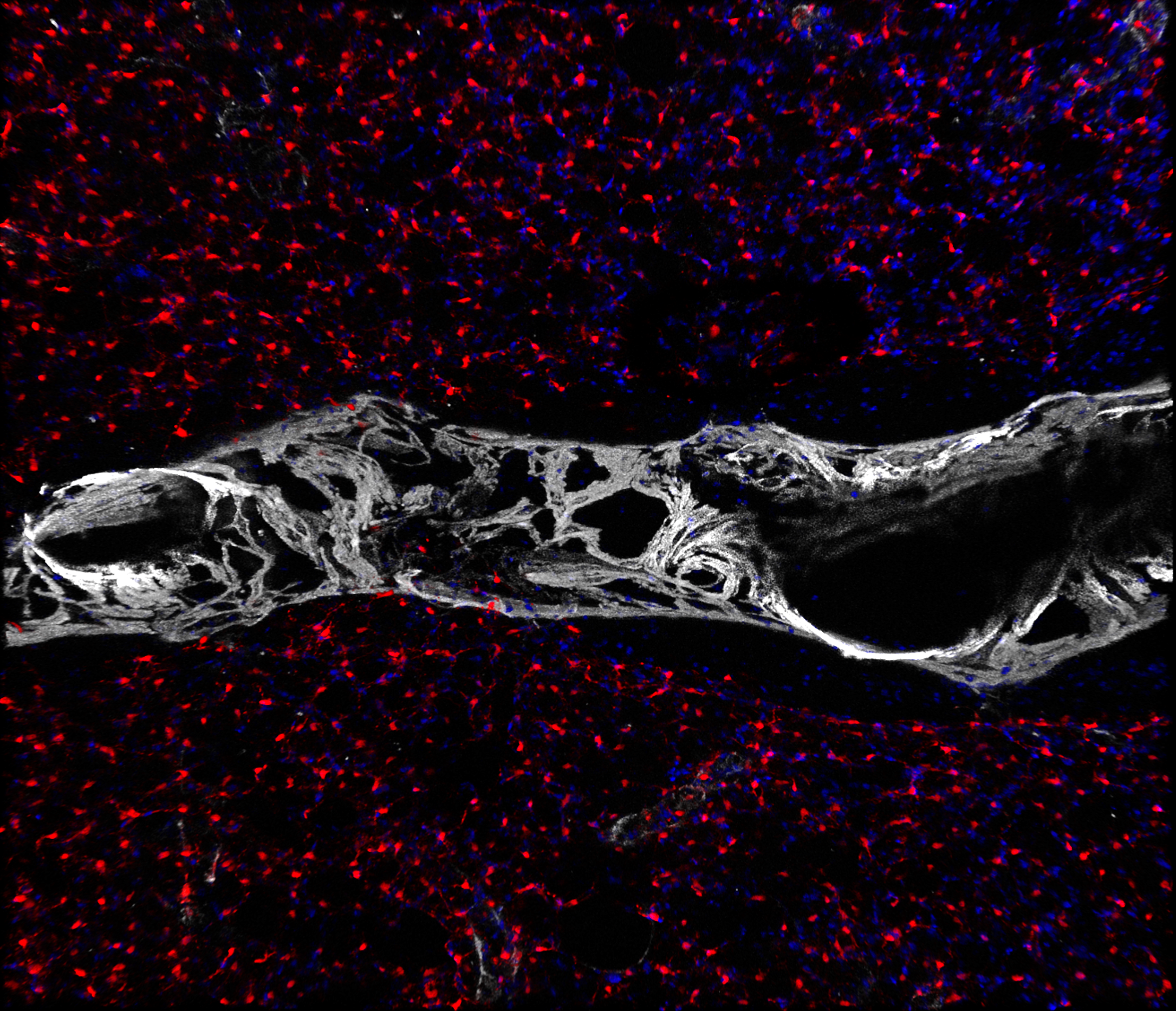

Supplement: Supplementary file 5 — Source data Fig. 4 [file 44318_2024_349_MOESM5_ESM.zip › 4A/vein1.png]

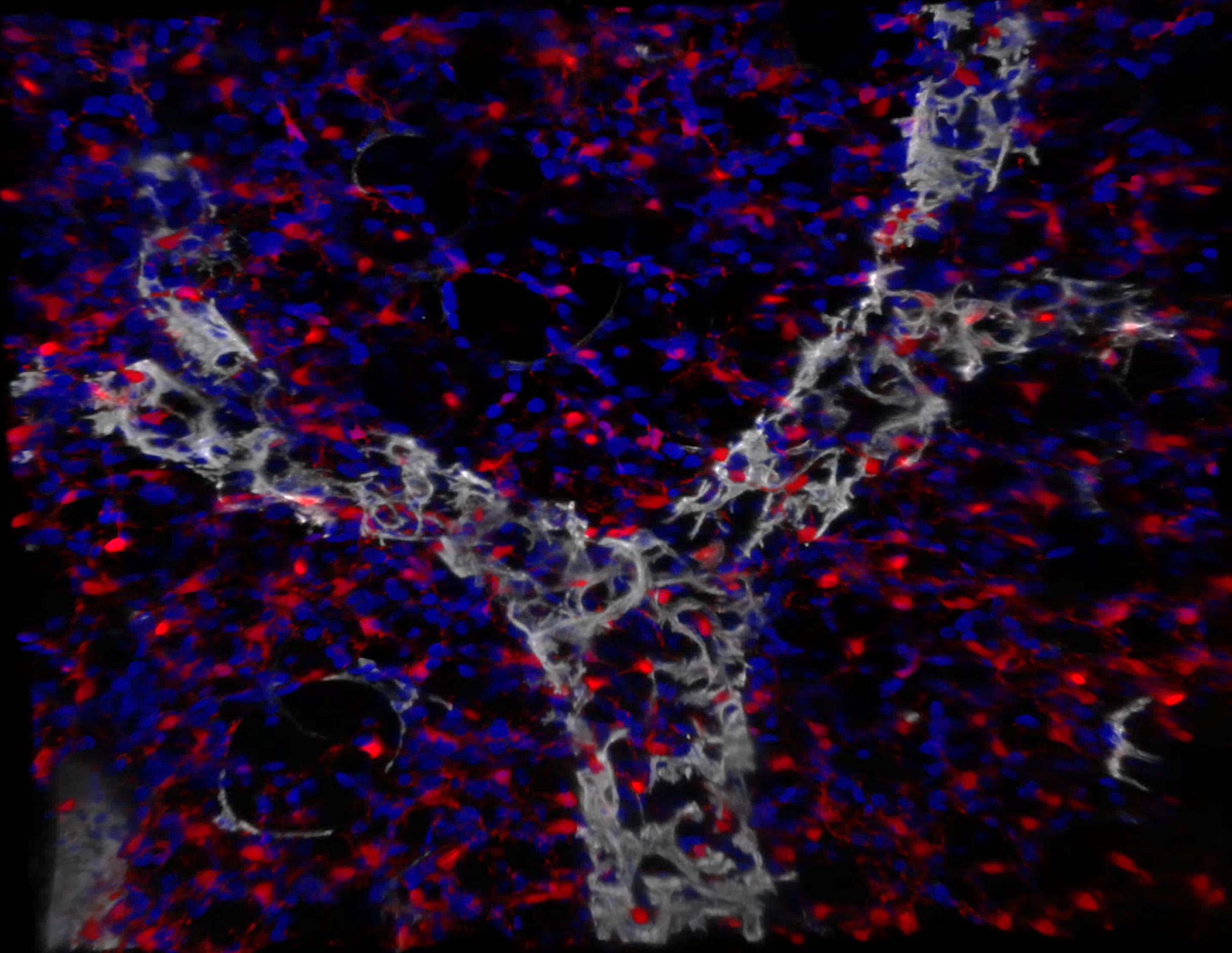

Supplement: Supplementary file 5 — Source data Fig. 4 [file 44318_2024_349_MOESM5_ESM.zip › 4A/vein2.png]

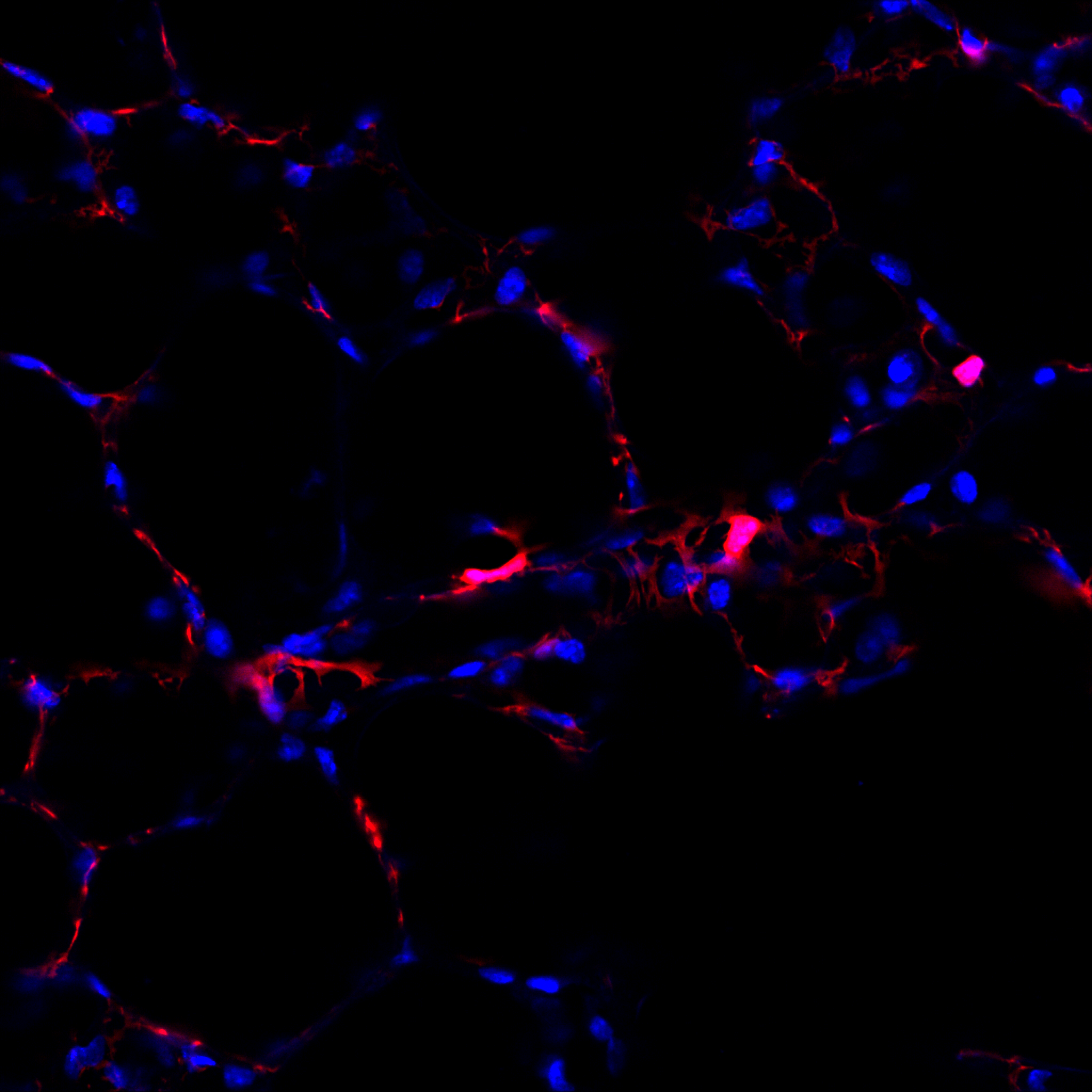

Supplement: Supplementary file 5 — Source data Fig. 4 [file 44318_2024_349_MOESM5_ESM.zip › 4B/1wk Hx tdT DAPI-3.tif]

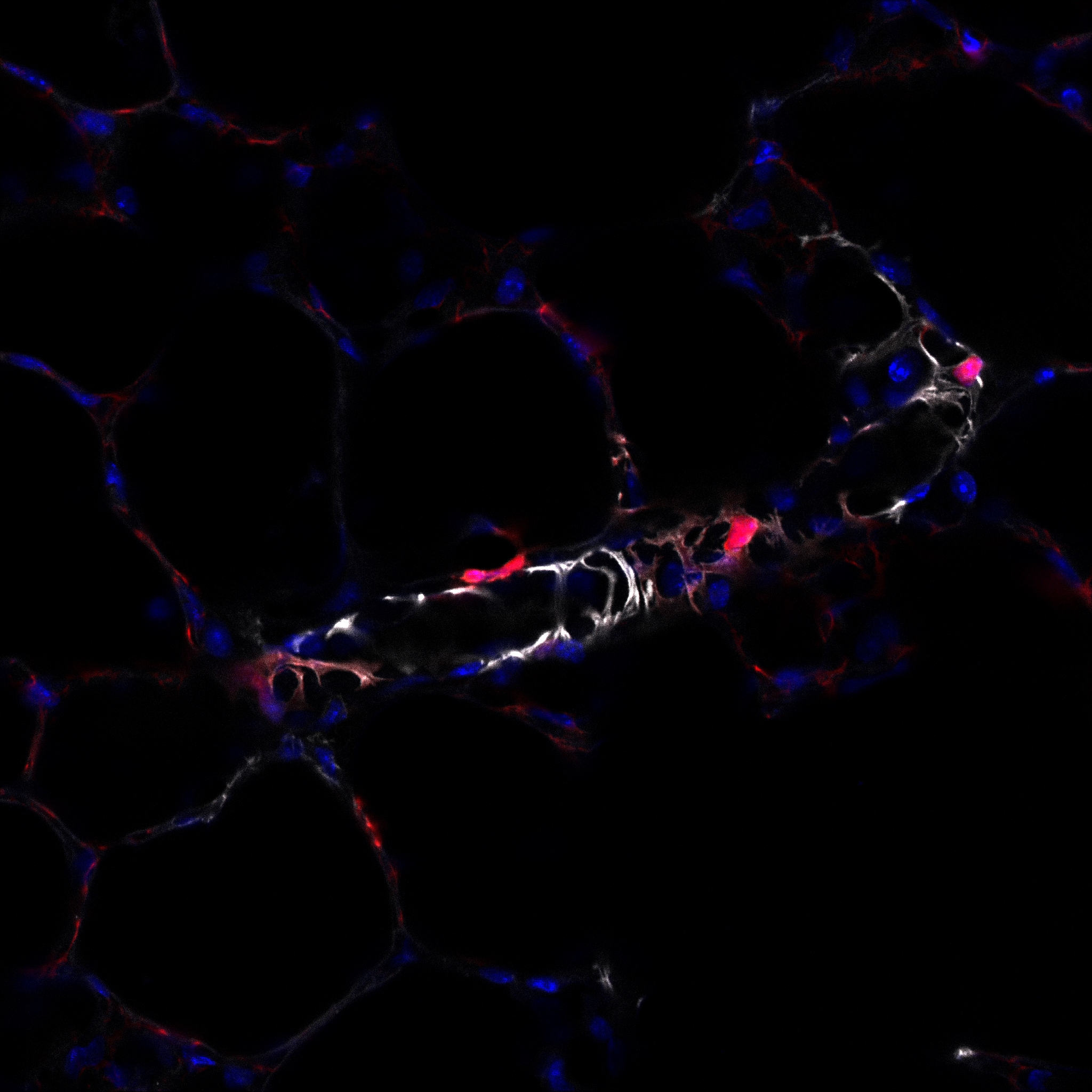

Supplement: Supplementary file 5 — Source data Fig. 4 [file 44318_2024_349_MOESM5_ESM.zip › 4B/1wk Hx-1.jpg]

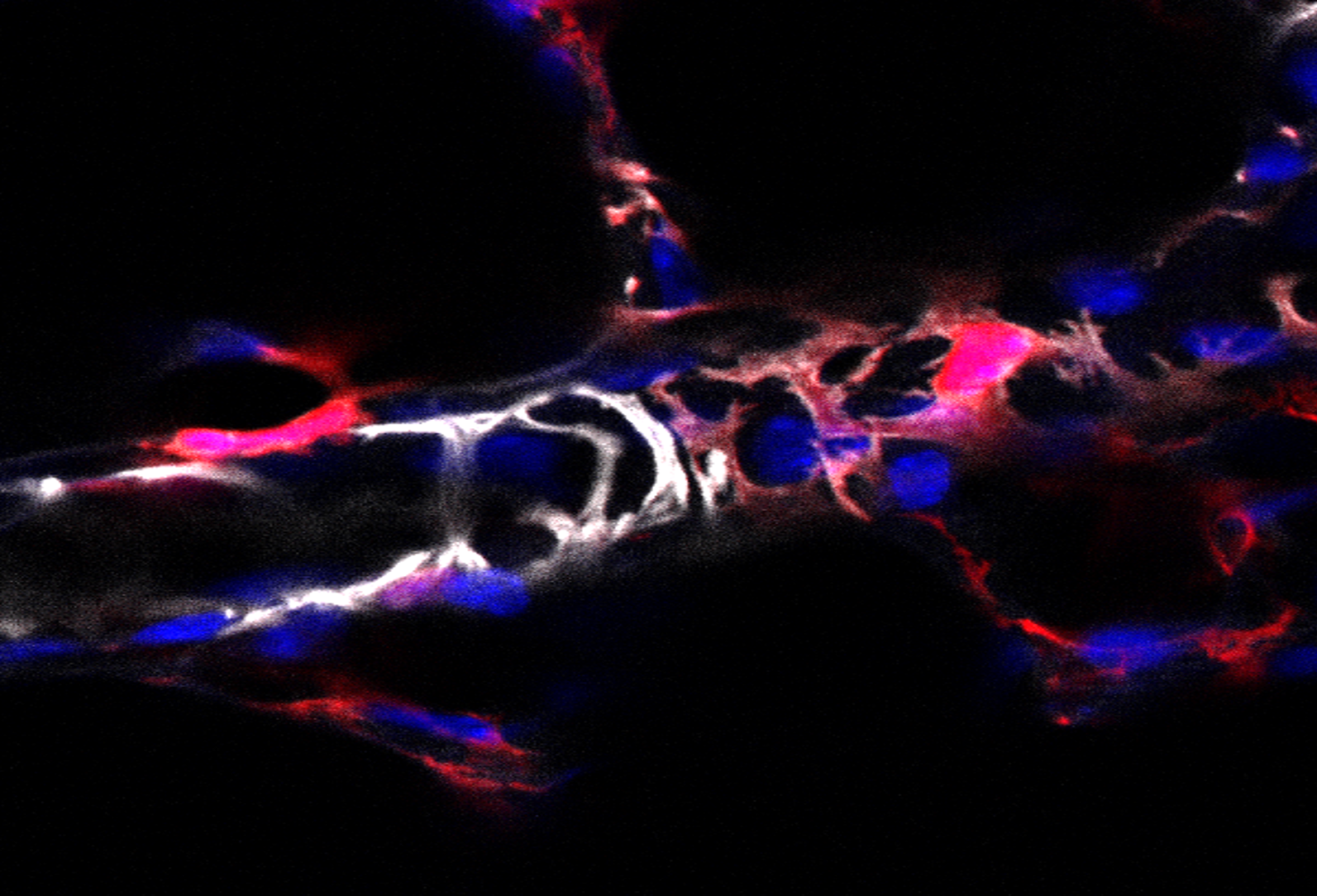

Supplement: Supplementary file 5 — Source data Fig. 4 [file 44318_2024_349_MOESM5_ESM.zip › 4B/1wk-2.png]

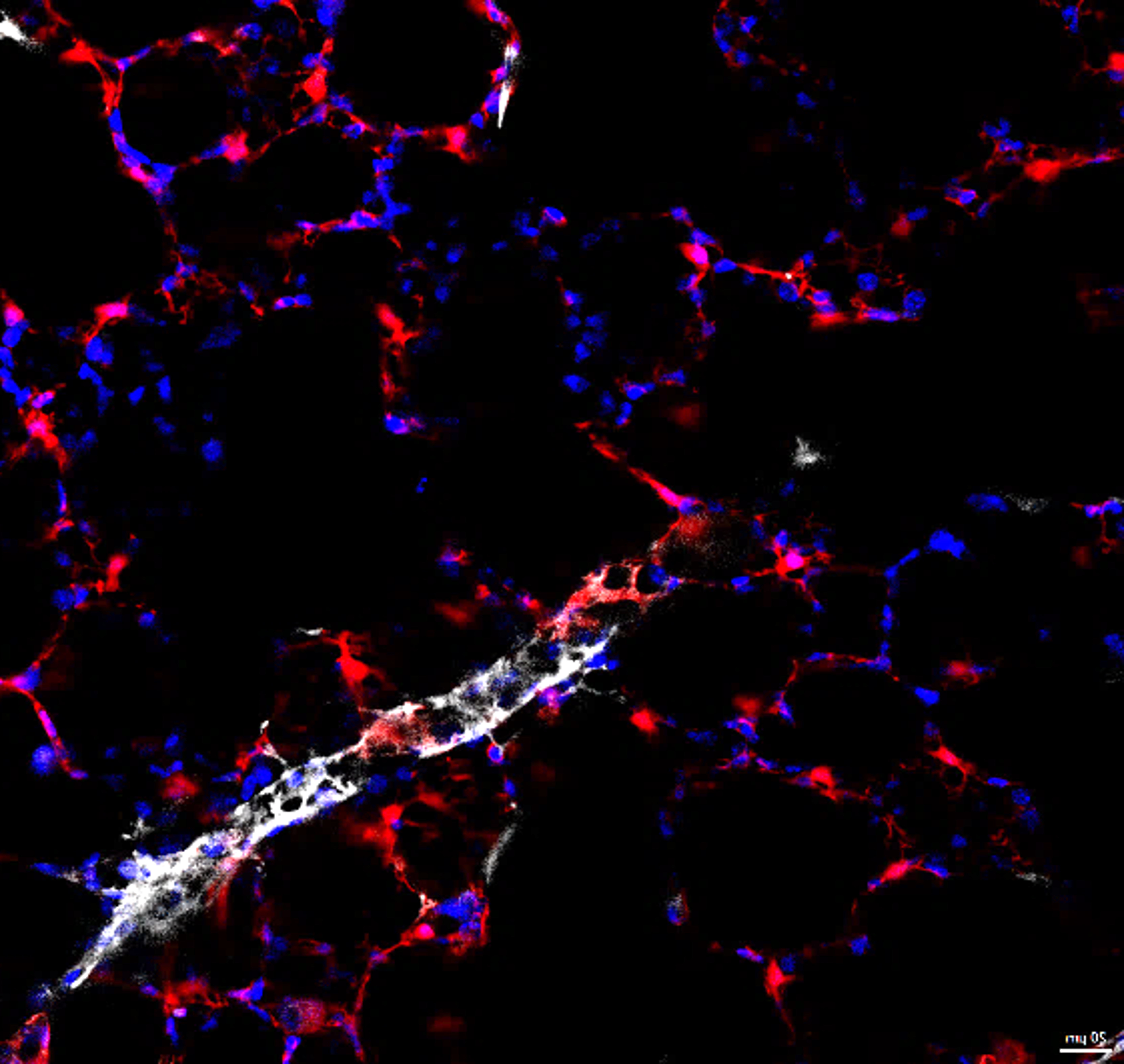

Supplement: Supplementary file 5 — Source data Fig. 4 [file 44318_2024_349_MOESM5_ESM.zip › 4B/2wk hx-1.png]

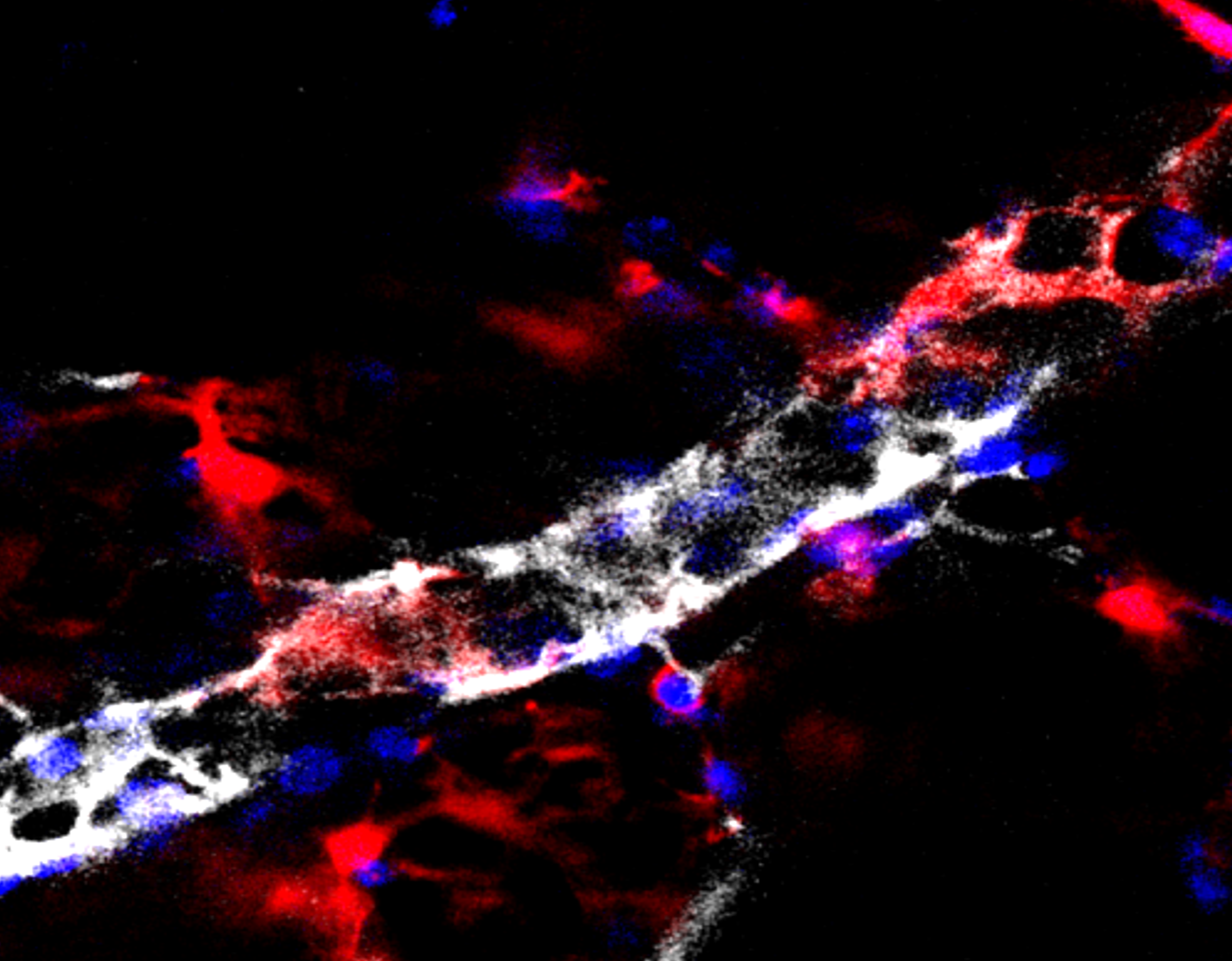

Supplement: Supplementary file 5 — Source data Fig. 4 [file 44318_2024_349_MOESM5_ESM.zip › 4B/2wk hx-2.png]

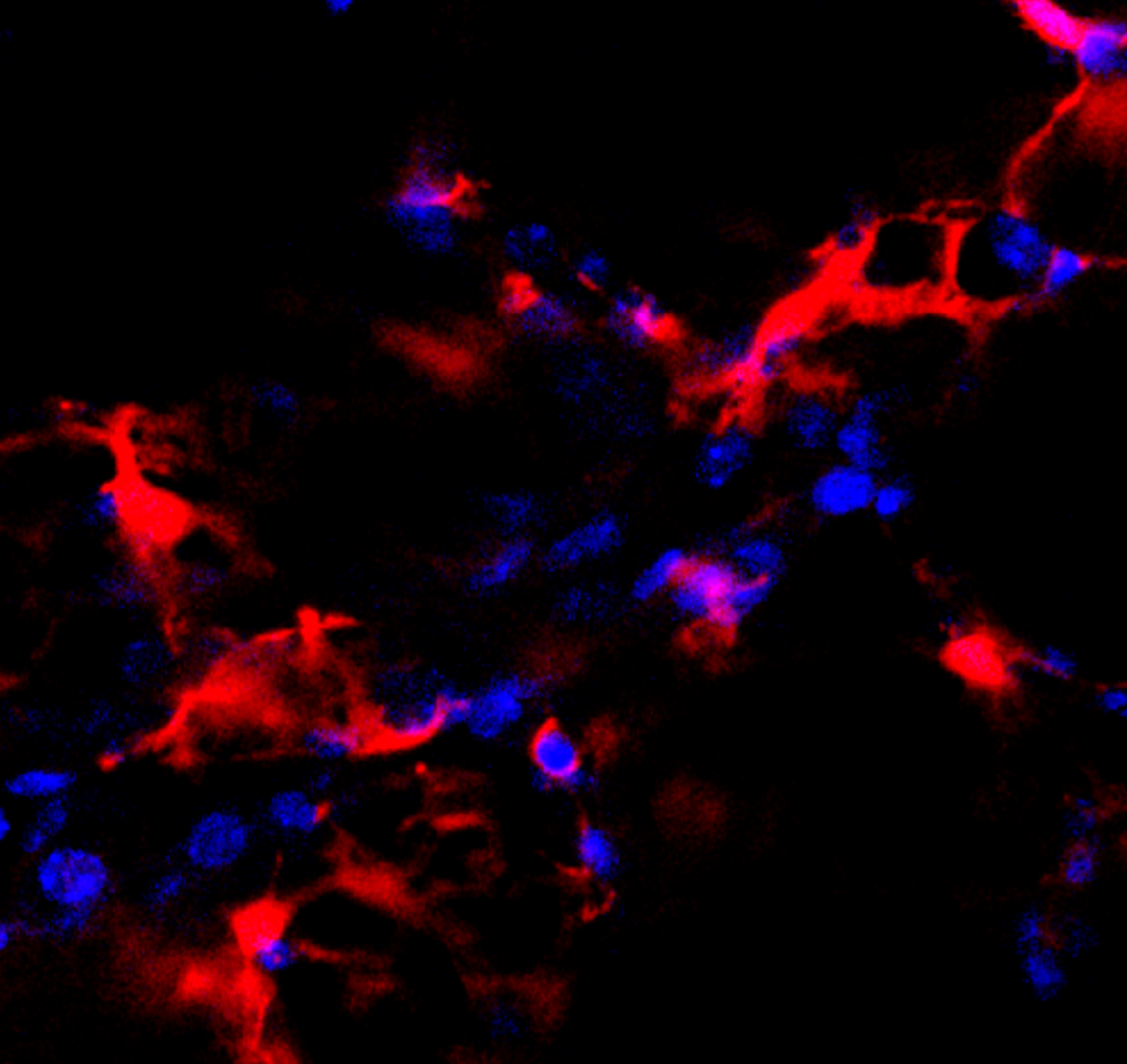

Supplement: Supplementary file 5 — Source data Fig. 4 [file 44318_2024_349_MOESM5_ESM.zip › 4B/2wk hx-3.png]

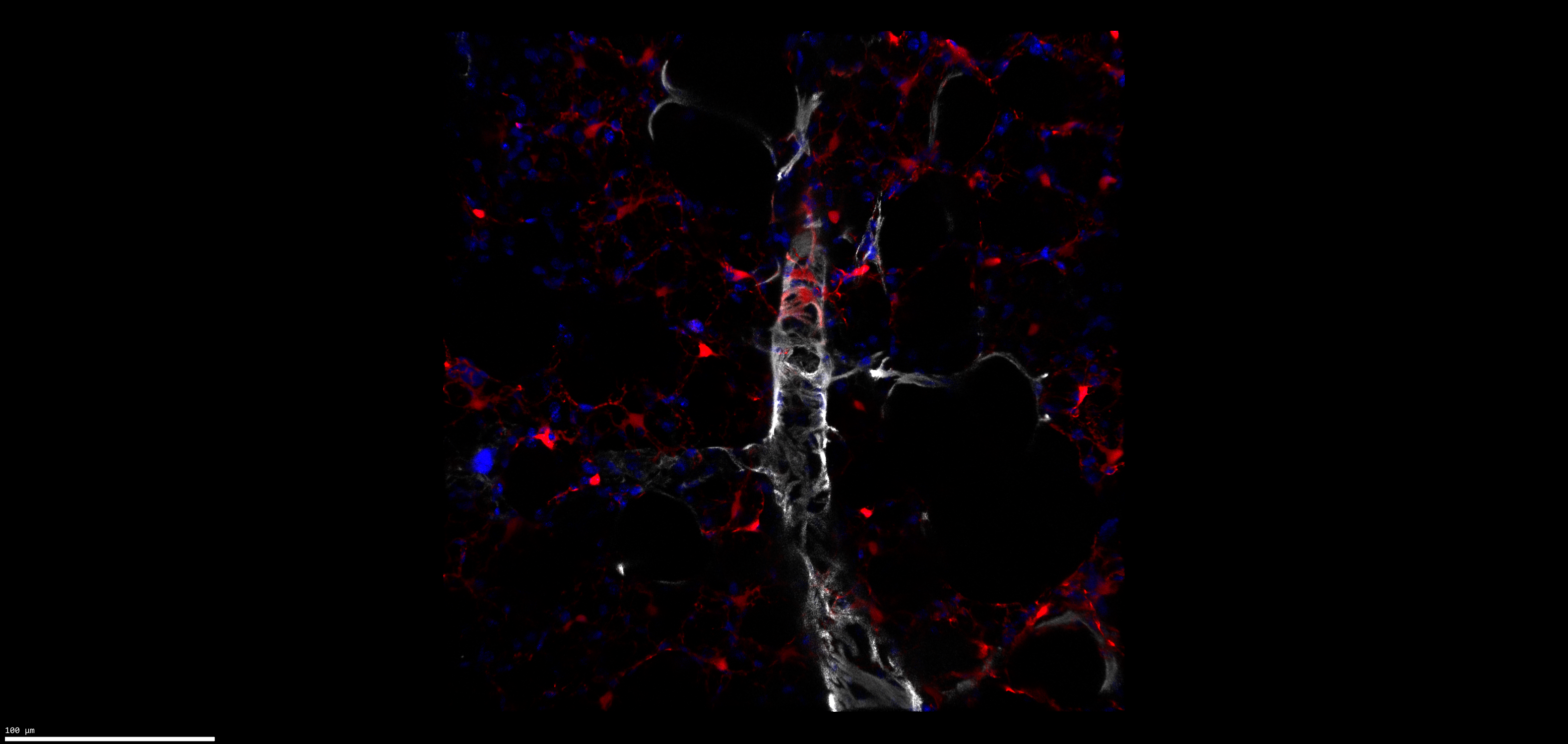

Supplement: Supplementary file 5 — Source data Fig. 4 [file 44318_2024_349_MOESM5_ESM.zip › 4B/3wk Hx.bmp]

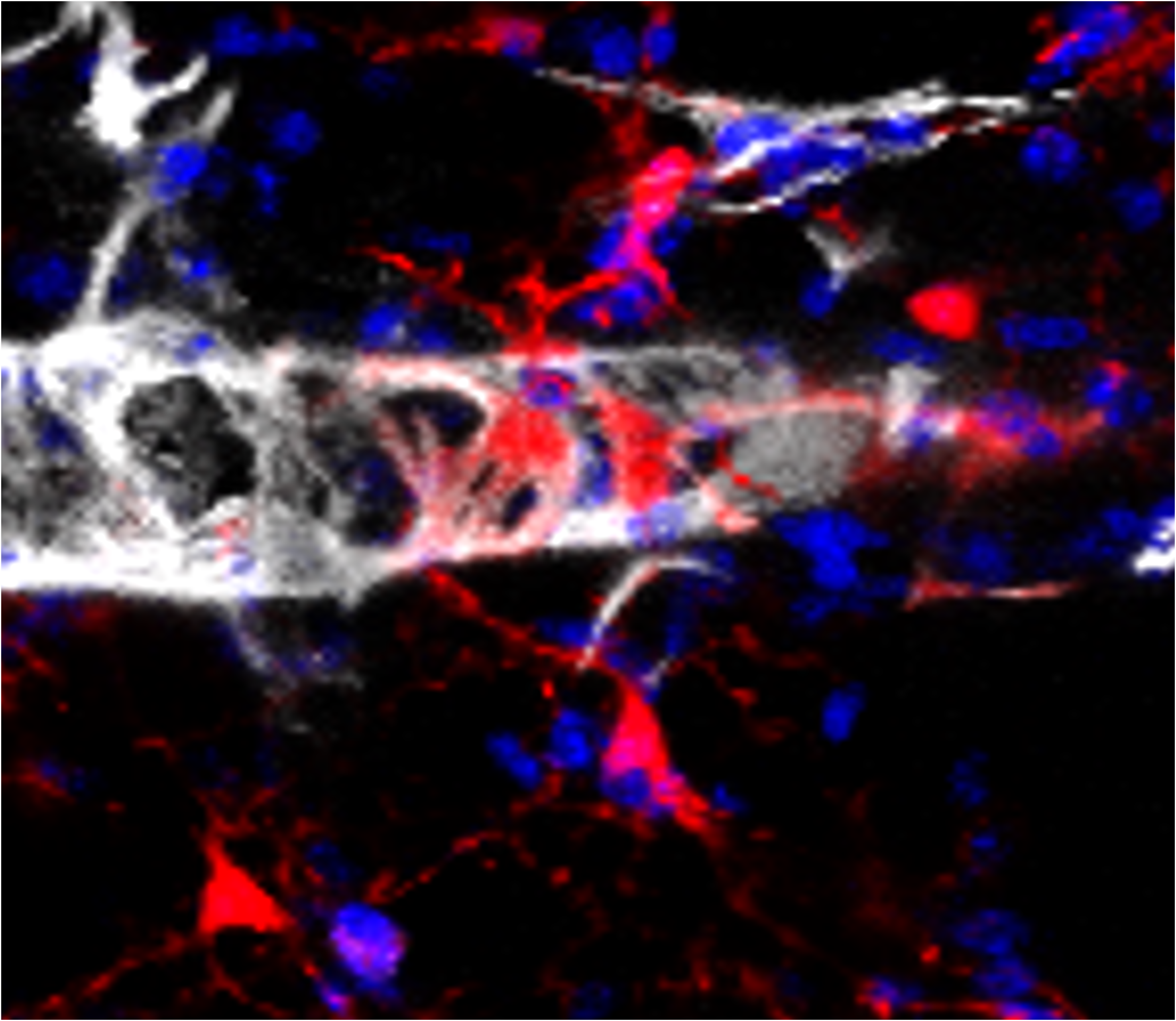

Supplement: Supplementary file 5 — Source data Fig. 4 [file 44318_2024_349_MOESM5_ESM.zip › 4B/3wk-hx-2.png]

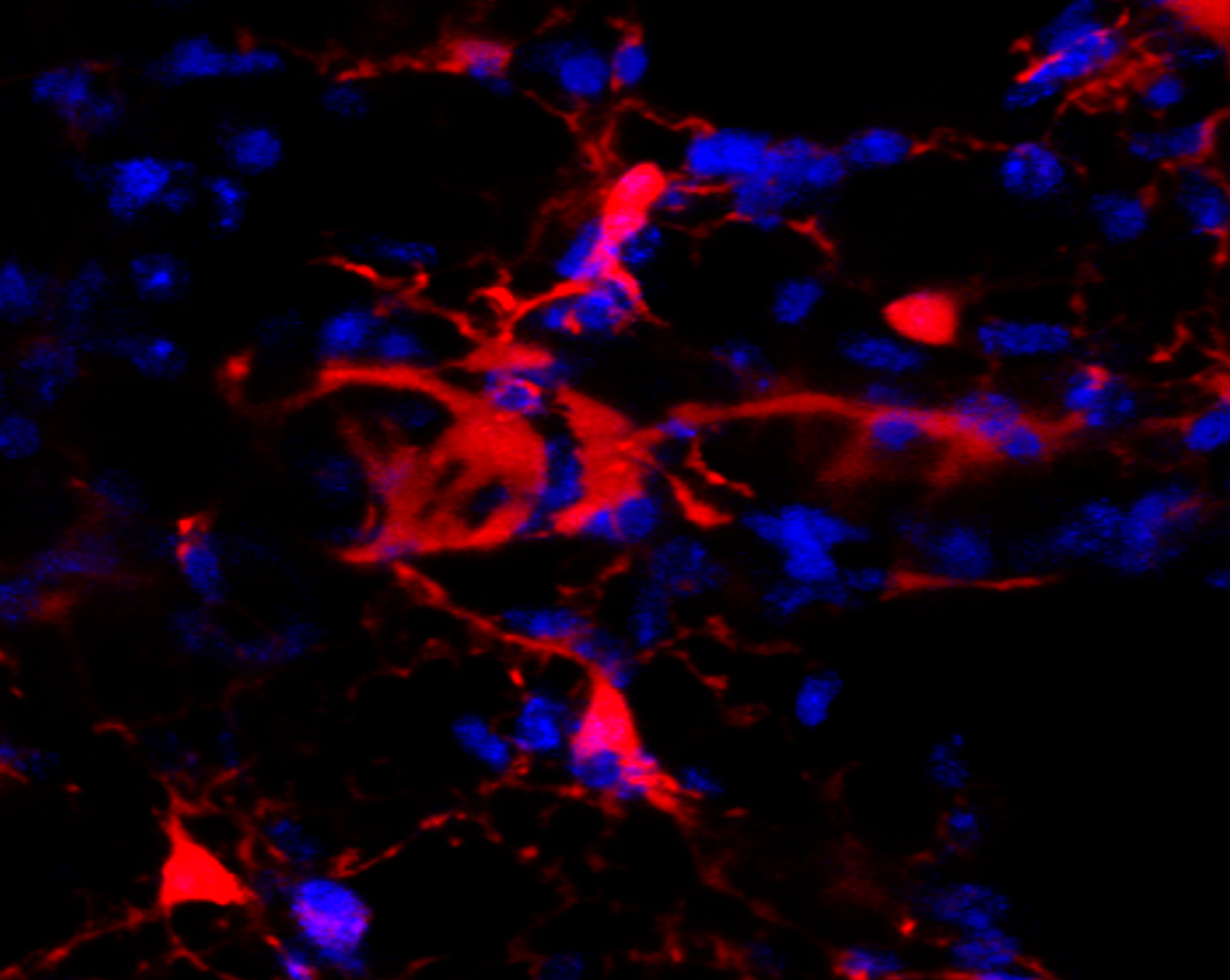

Supplement: Supplementary file 5 — Source data Fig. 4 [file 44318_2024_349_MOESM5_ESM.zip › 4B/3wk-hx-3.png]

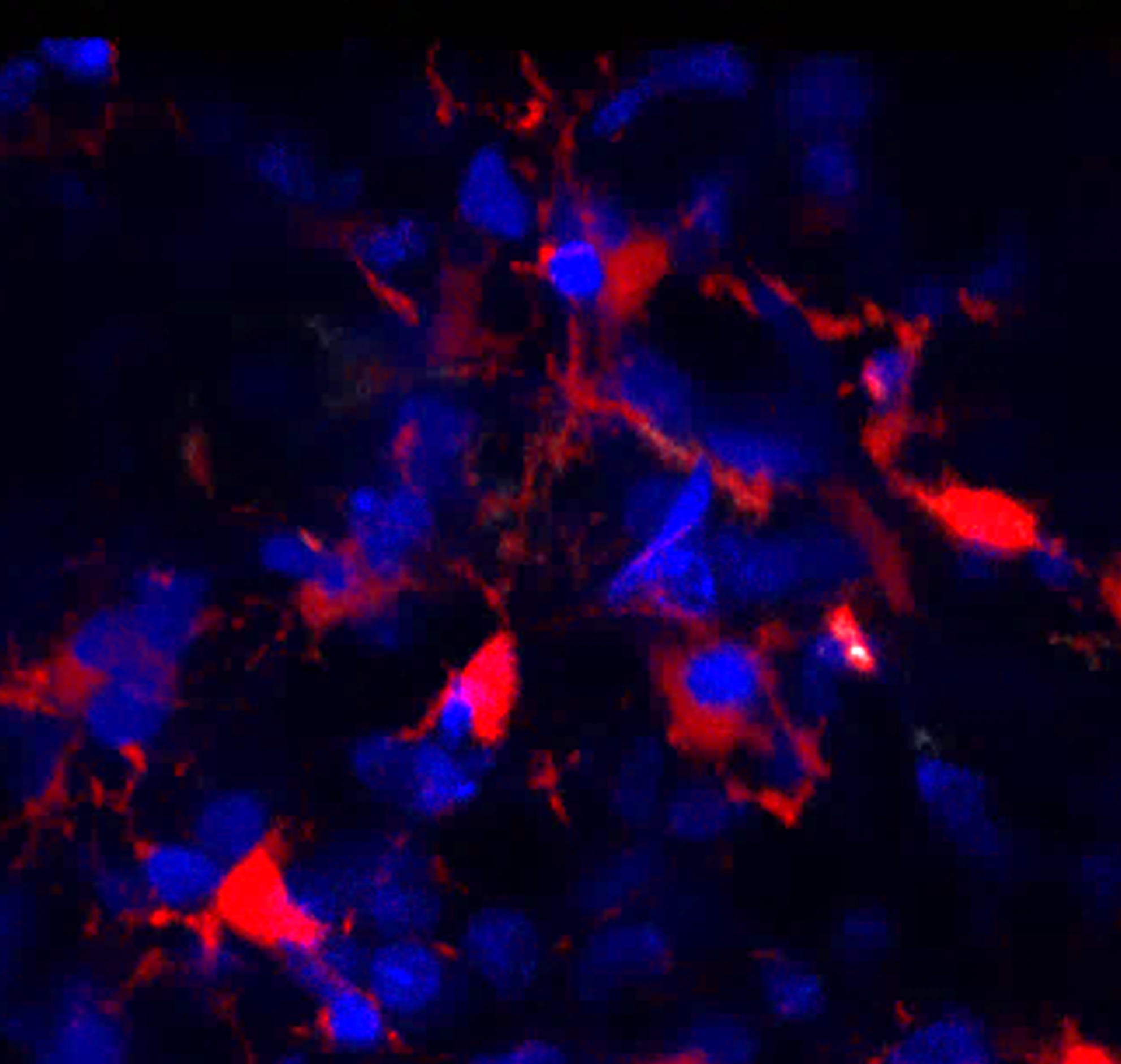

Supplement: Supplementary file 5 — Source data Fig. 4 [file 44318_2024_349_MOESM5_ESM.zip › 4B/norm-2.png]

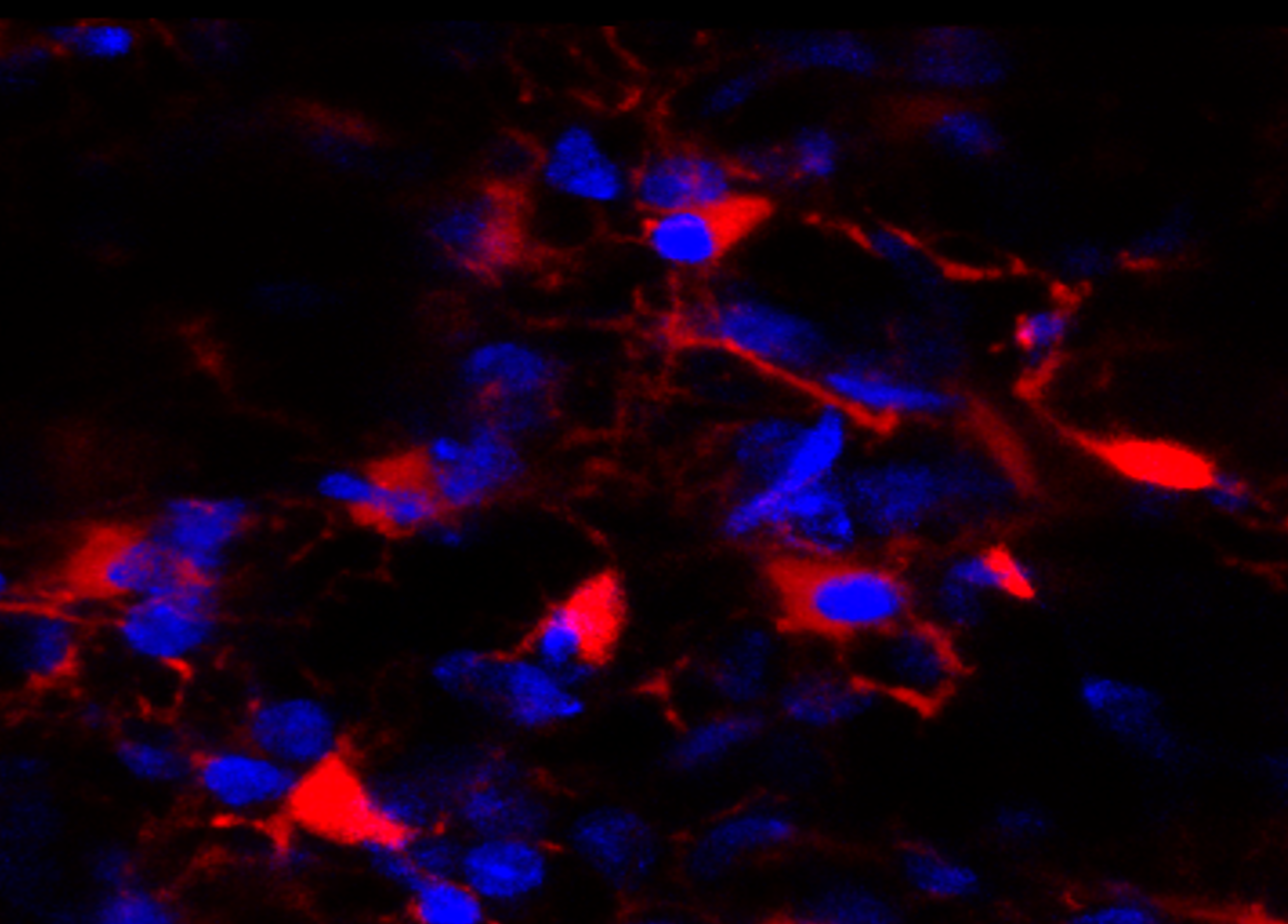

Supplement: Supplementary file 5 — Source data Fig. 4 [file 44318_2024_349_MOESM5_ESM.zip › 4B/norm-3.png]

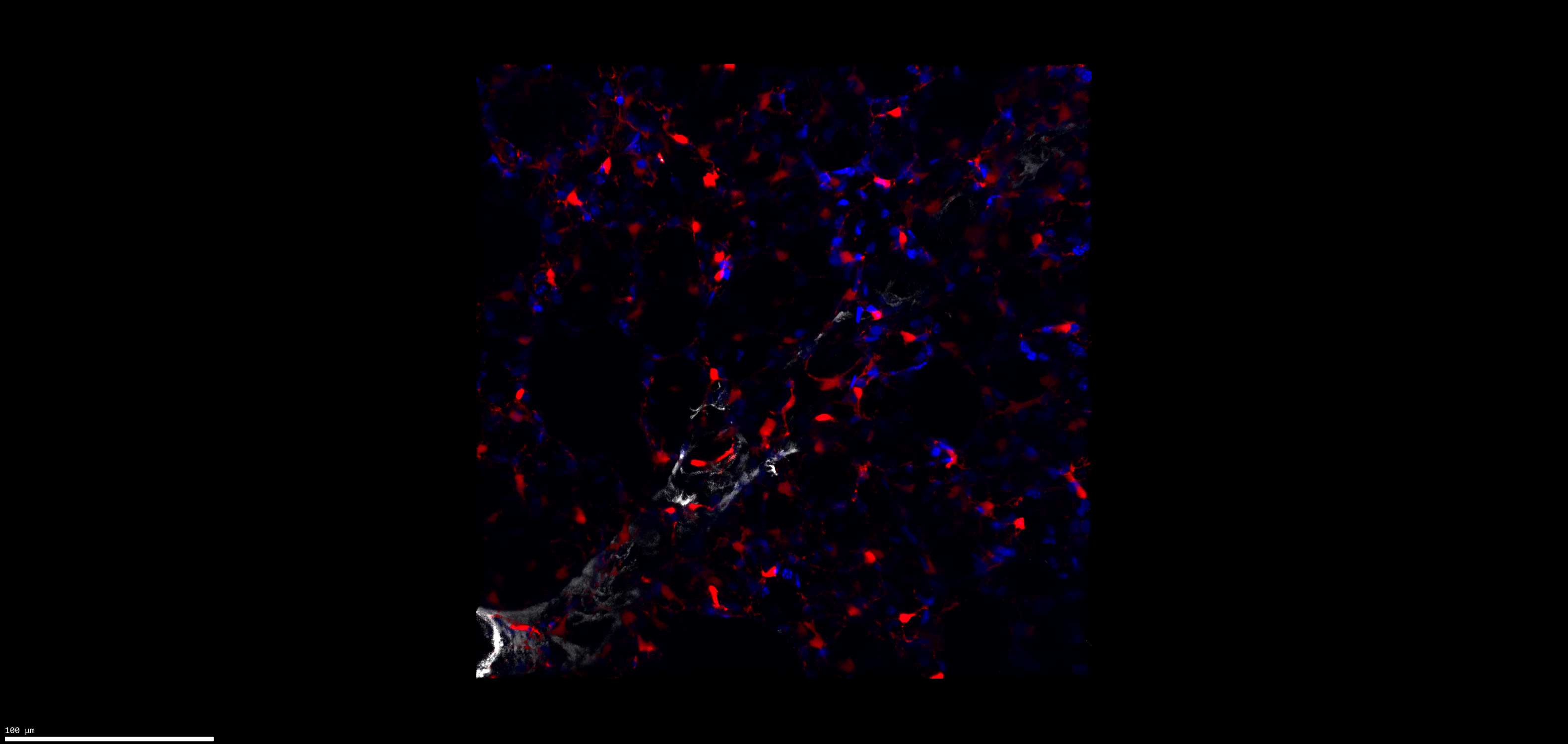

Supplement: Supplementary file 5 — Source data Fig. 4 [file 44318_2024_349_MOESM5_ESM.zip › 4B/Nornoxia.bmp]

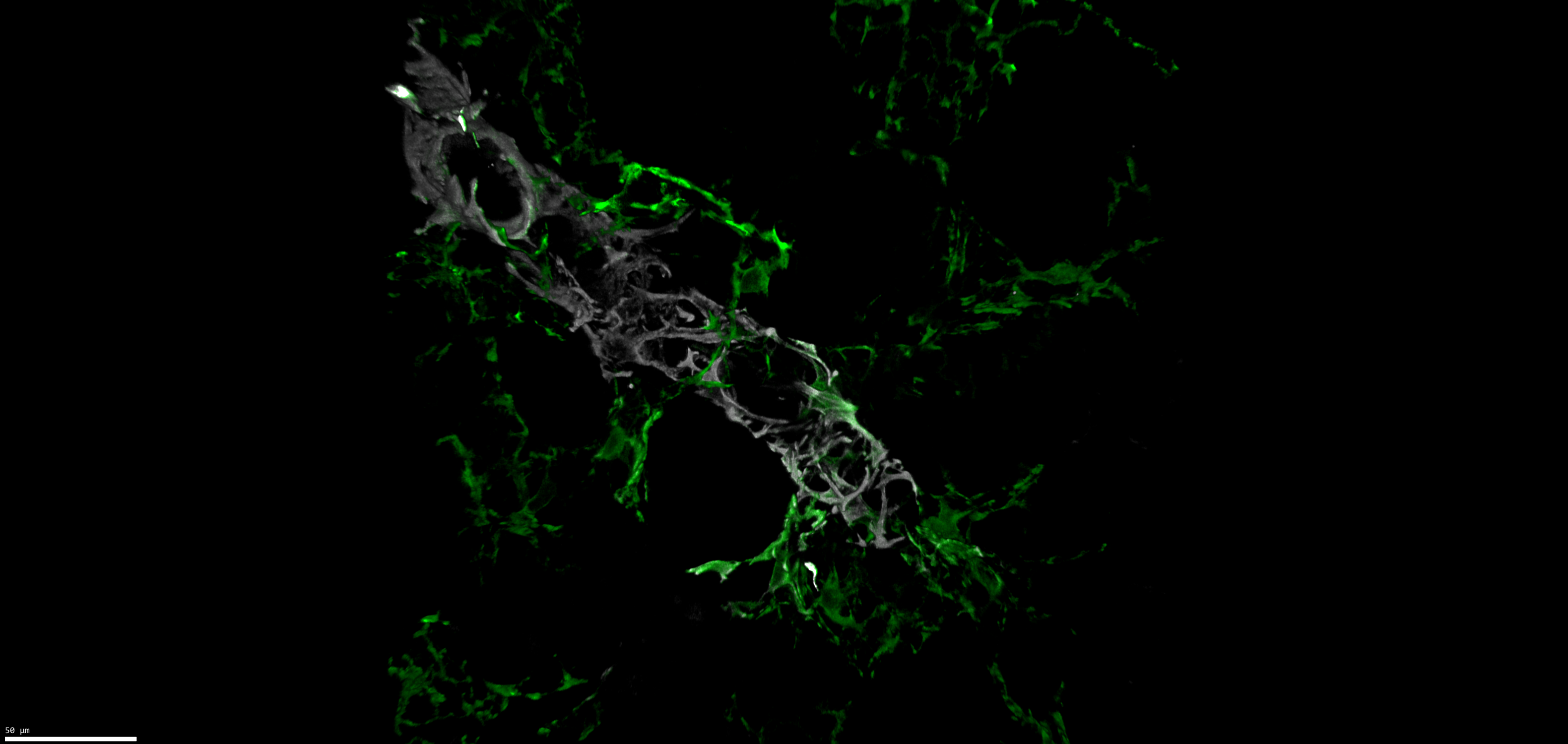

Supplement: Supplementary file 5 — Source data Fig. 4 [file 44318_2024_349_MOESM5_ESM.zip › 4C/3wk Hx GFP SMA DAPI.bmp]

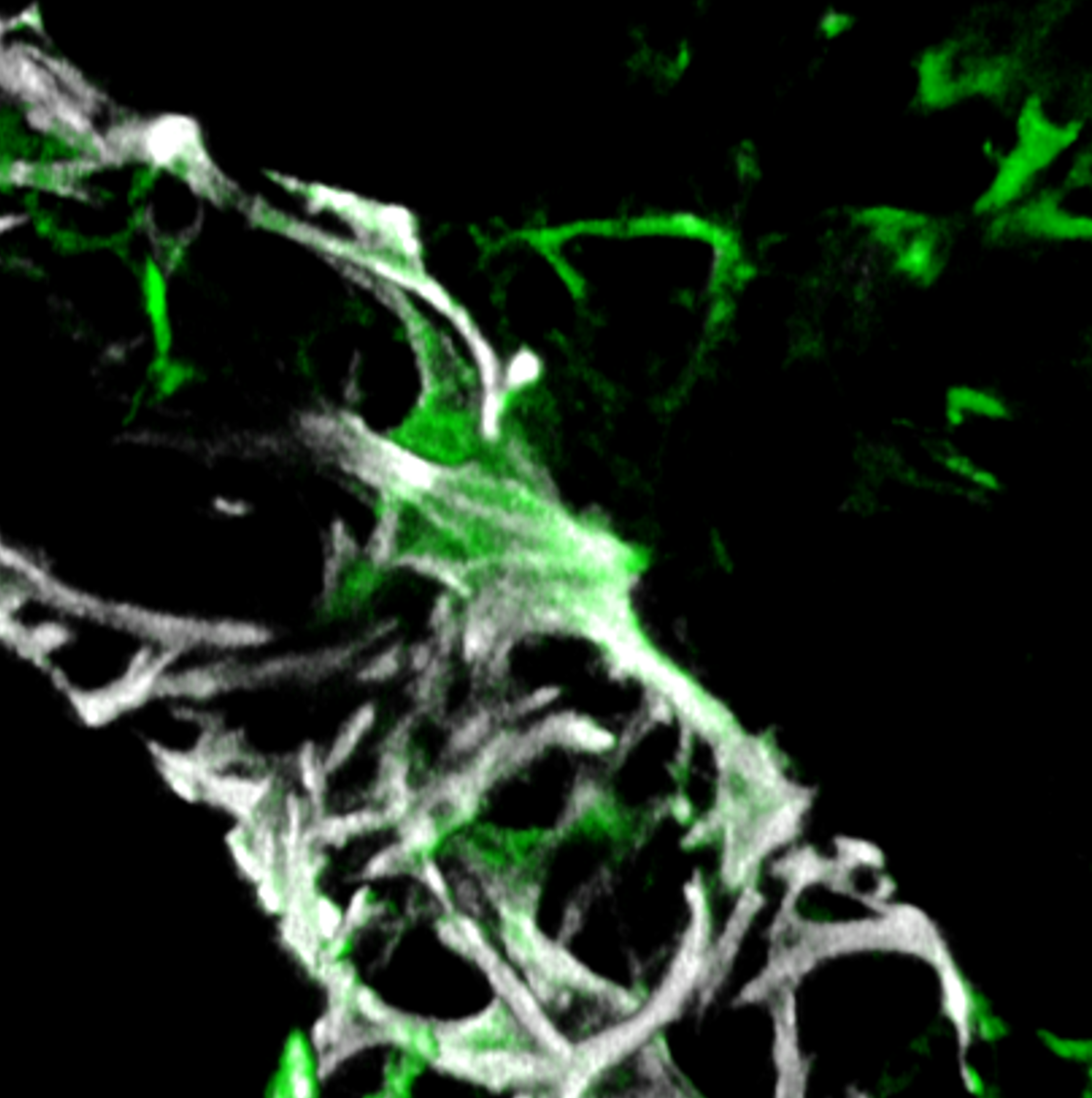

Supplement: Supplementary file 5 — Source data Fig. 4 [file 44318_2024_349_MOESM5_ESM.zip › 4C/3wk-2.png]

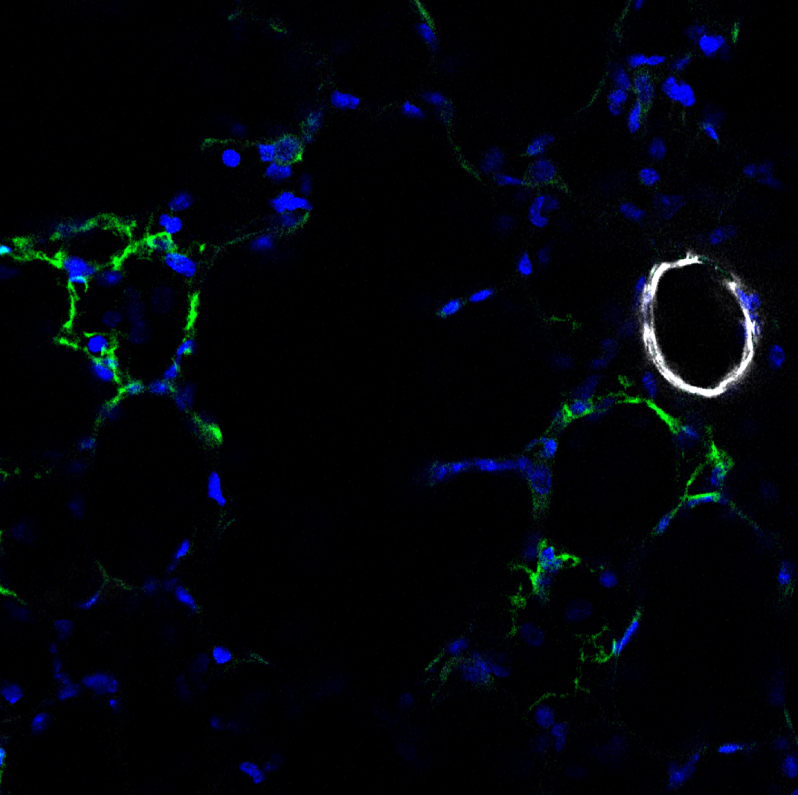

Supplement: Supplementary file 5 — Source data Fig. 4 [file 44318_2024_349_MOESM5_ESM.zip › 4C/Normoxia.tif]

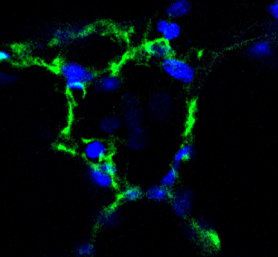

Supplement: Supplementary file 5 — Source data Fig. 4 [file 44318_2024_349_MOESM5_ESM.zip › 4C/Normxia crop.tif]

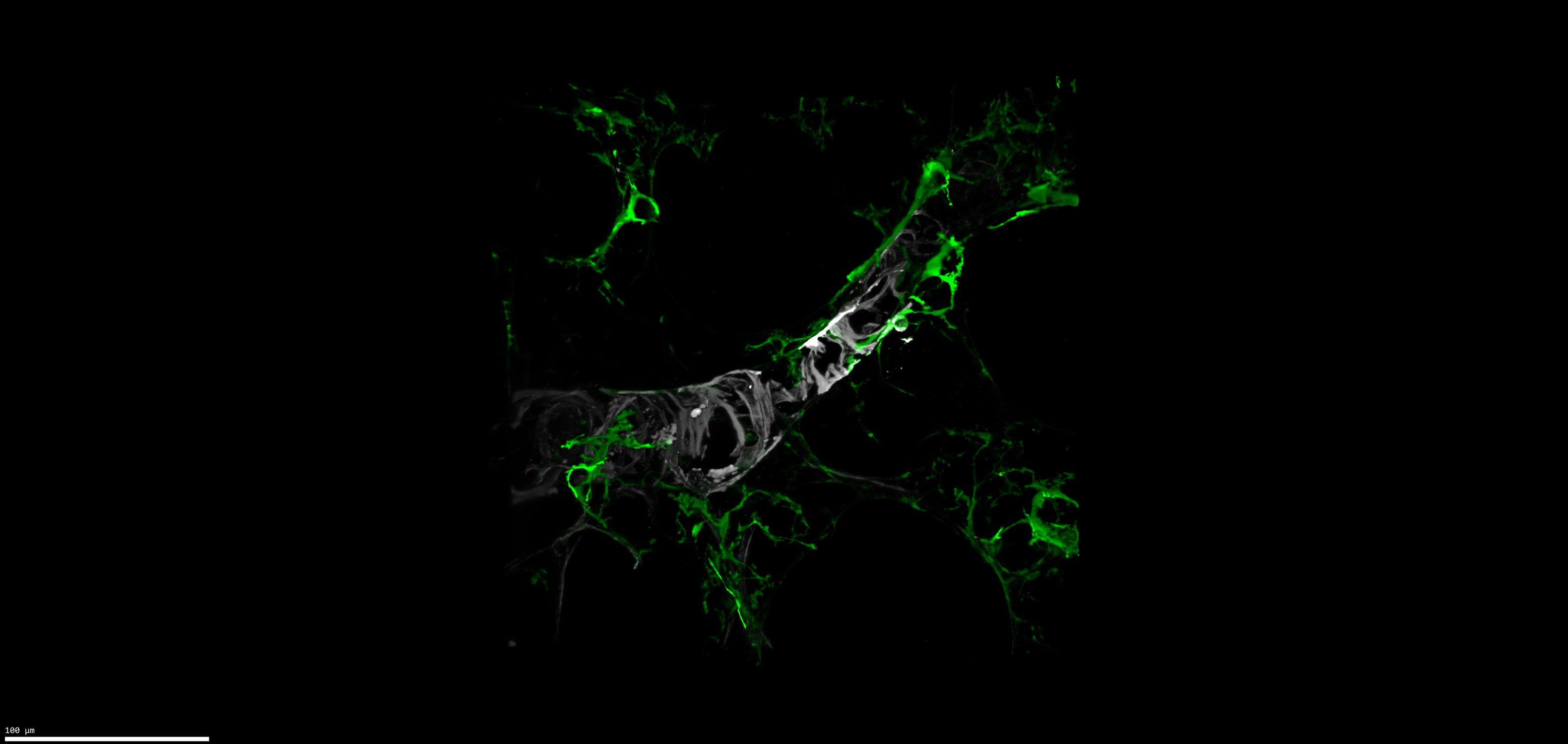

Supplement: Supplementary file 5 — Source data Fig. 4 [file 44318_2024_349_MOESM5_ESM.zip › 4D/mTmG recovery GFP SMA.bmp]

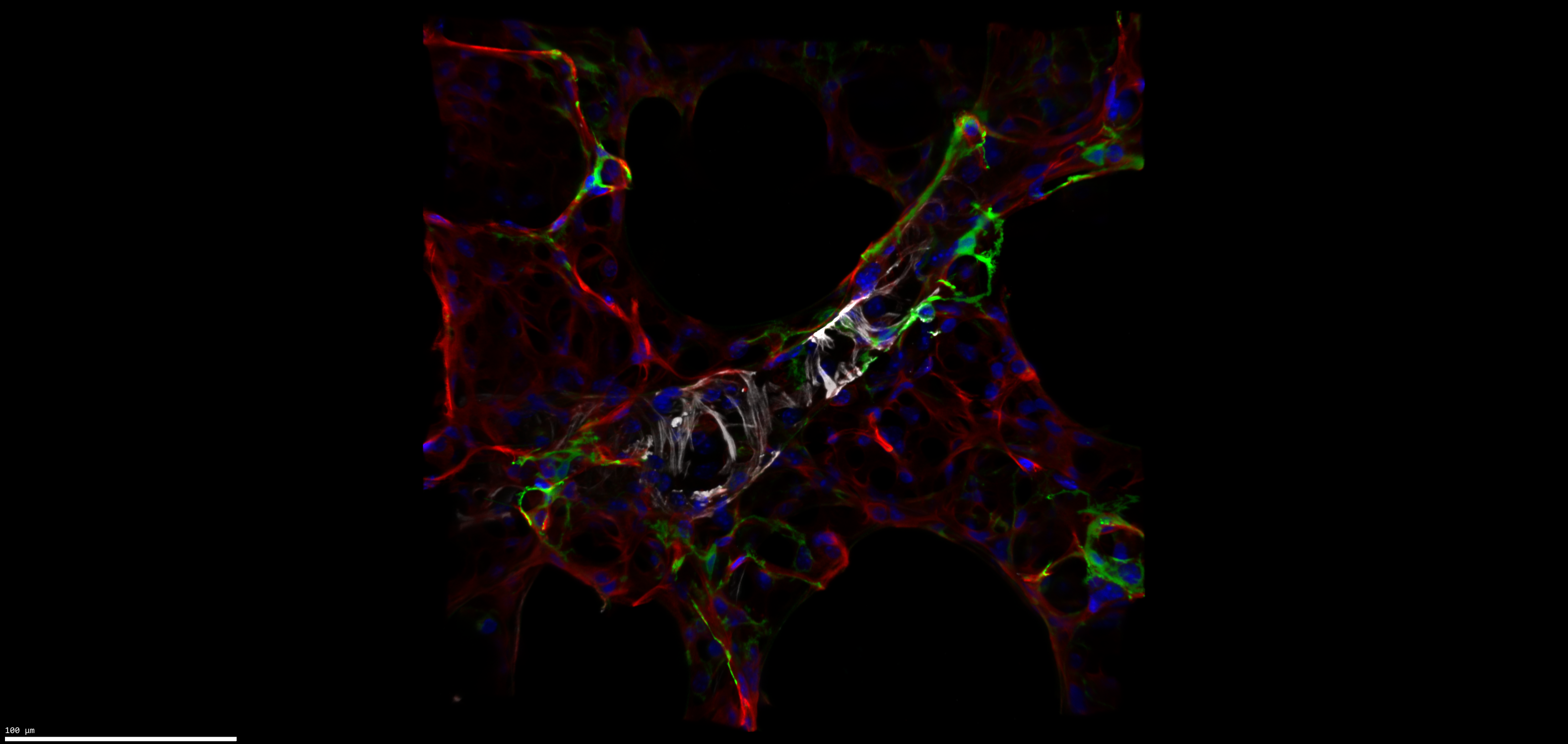

Supplement: Supplementary file 5 — Source data Fig. 4 [file 44318_2024_349_MOESM5_ESM.zip › 4D/mTmG recovery Merge.bmp]

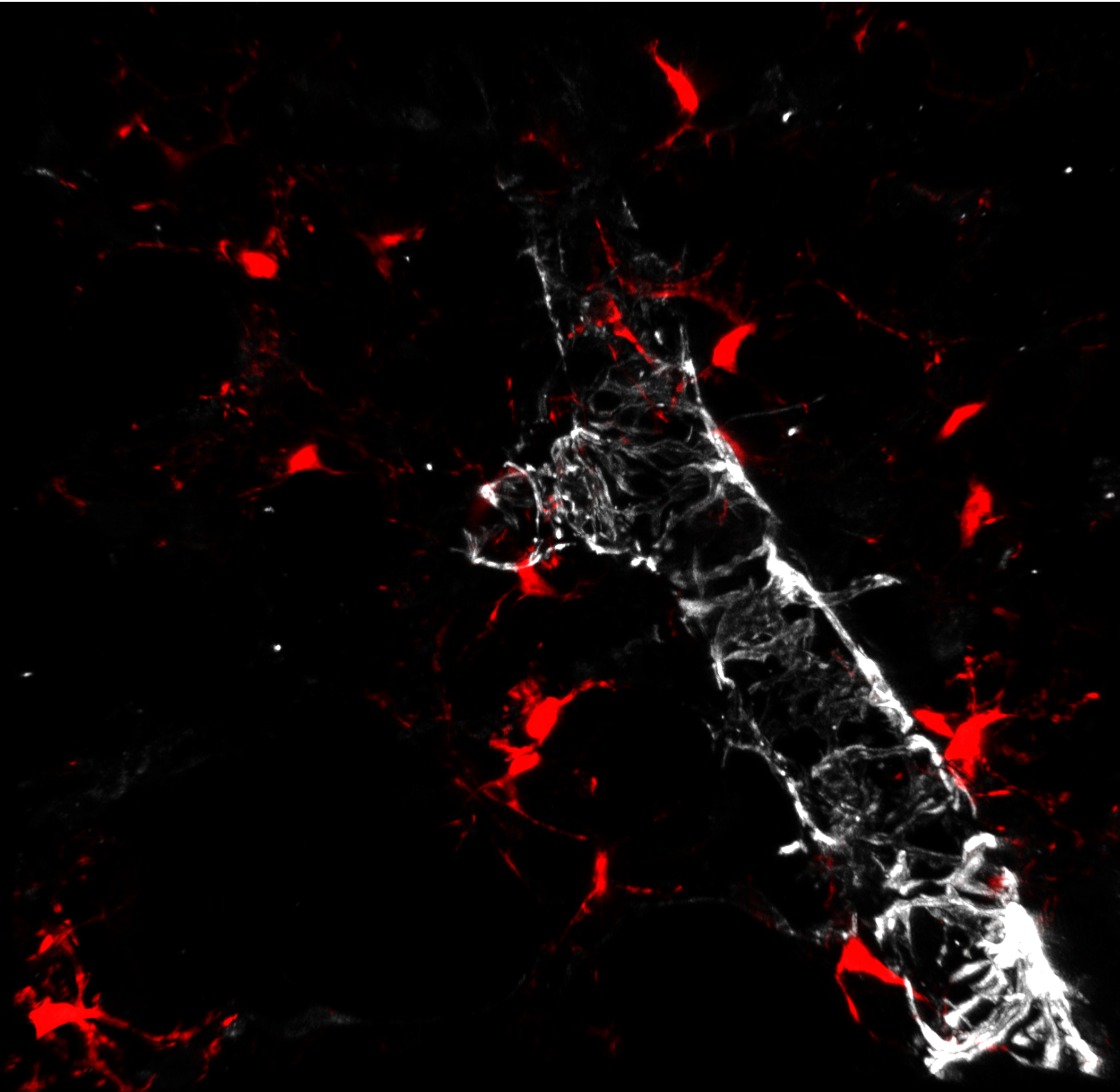

Supplement: Supplementary file 5 — Source data Fig. 4 [file 44318_2024_349_MOESM5_ESM.zip › 4D/tdT recovery -1.png]

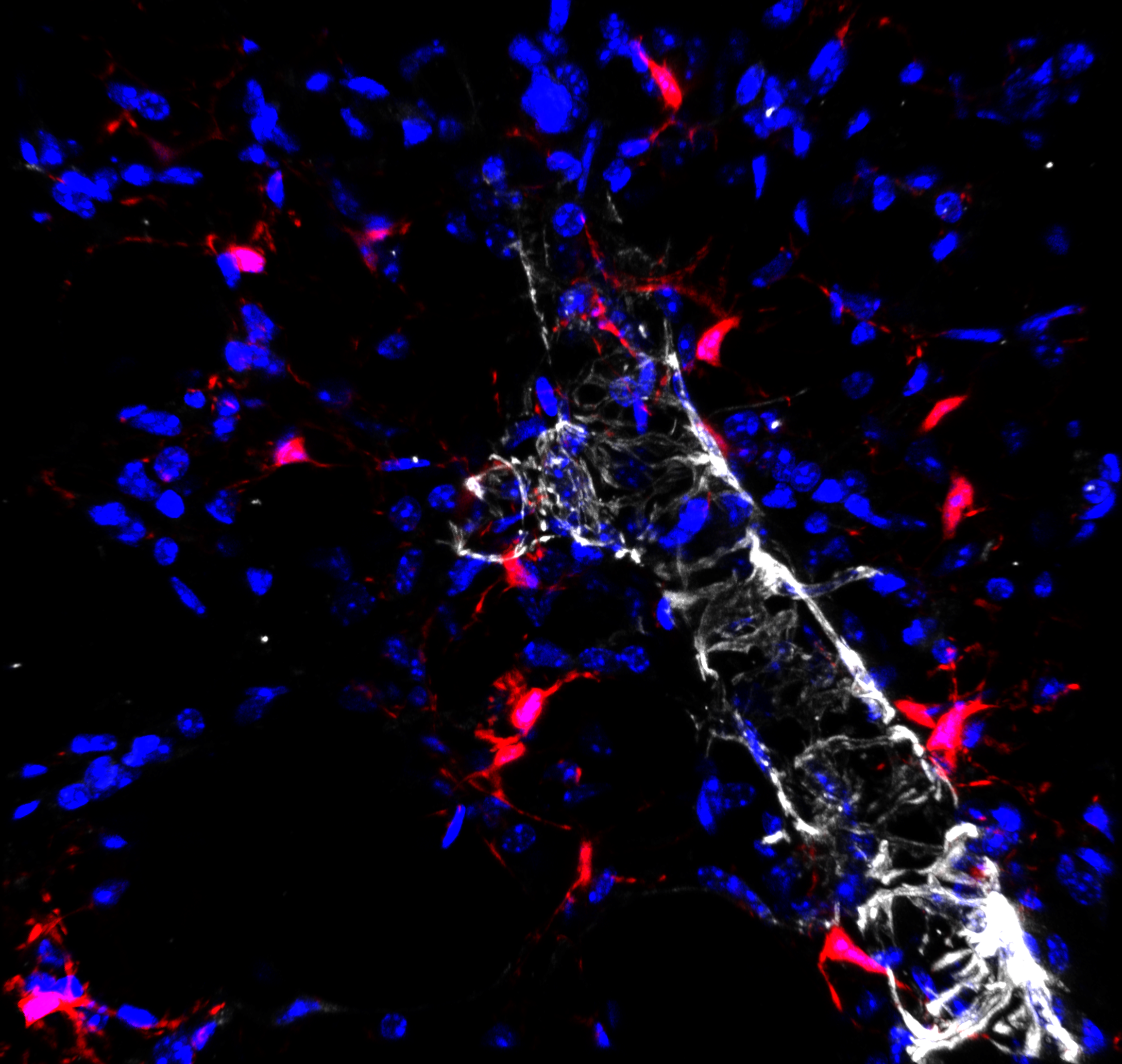

Supplement: Supplementary file 5 — Source data Fig. 4 [file 44318_2024_349_MOESM5_ESM.zip › 4D/tdT recovery -2.png]
