# Supplementary material for: Specialized pericyte subtypes in the pulmonary capillaries
Source: EMBO J. 2025 Jan 13;44(4):1074–106. doi: 10.1038/s44318-024-00349-1 (PMC11833098; doi:10.1038/s44318-024-00349-1)
Supplement: Supplementary file 9 — Expanded View Figures [file 44318_2024_349_MOESM9_ESM.pdf]

Expanded View Figures

Figure EV1. *HIGD1B* is expressed in human lung PCs.

(**A**) UMAP visualization of cell populations within human lung tissue using the original UMAP coordinates and cell type annotations from the 'Human Lung Cell Atlas (core)'. Cell type annotations are color-coded, as shown in the dot plot. PC and SMC distributions within lung tissues, using original UMAP coordinates and cell annotations from the 'HLCA (core)' are highlighted in the bottom left. In UMAP, Dot, Violin and Heatmap plots, PCs were highlighted in light orange and SMCs in dark red. The Dot and Violin plots on the right illustrate the expression pattern of PC (*CSPG4, PDGFRB*, and *HIGD1B*) and SMC markers (*ACTA2*, *CNN1*, and *TAGLN*) across all annotated cell types. The Dot plot highlights the relative expression of markers, while the Violin plot depicts the distribution and intensity of expression within each cell type. (**B**) Differential expression (DE) analysis utilizing the Wilcoxon rank-sum test shows the expression profiles of PC markers (*CSPG4, PDGFRB*, and *HIGD1B*) and SMC markers (*ACTA2*, *CNN1*, and *TAGLN*) in annotated PCs and SMCs compared to all other cell populations within the 'Human Lung Cell Atlas (core)'.

 © The Author(s)

A

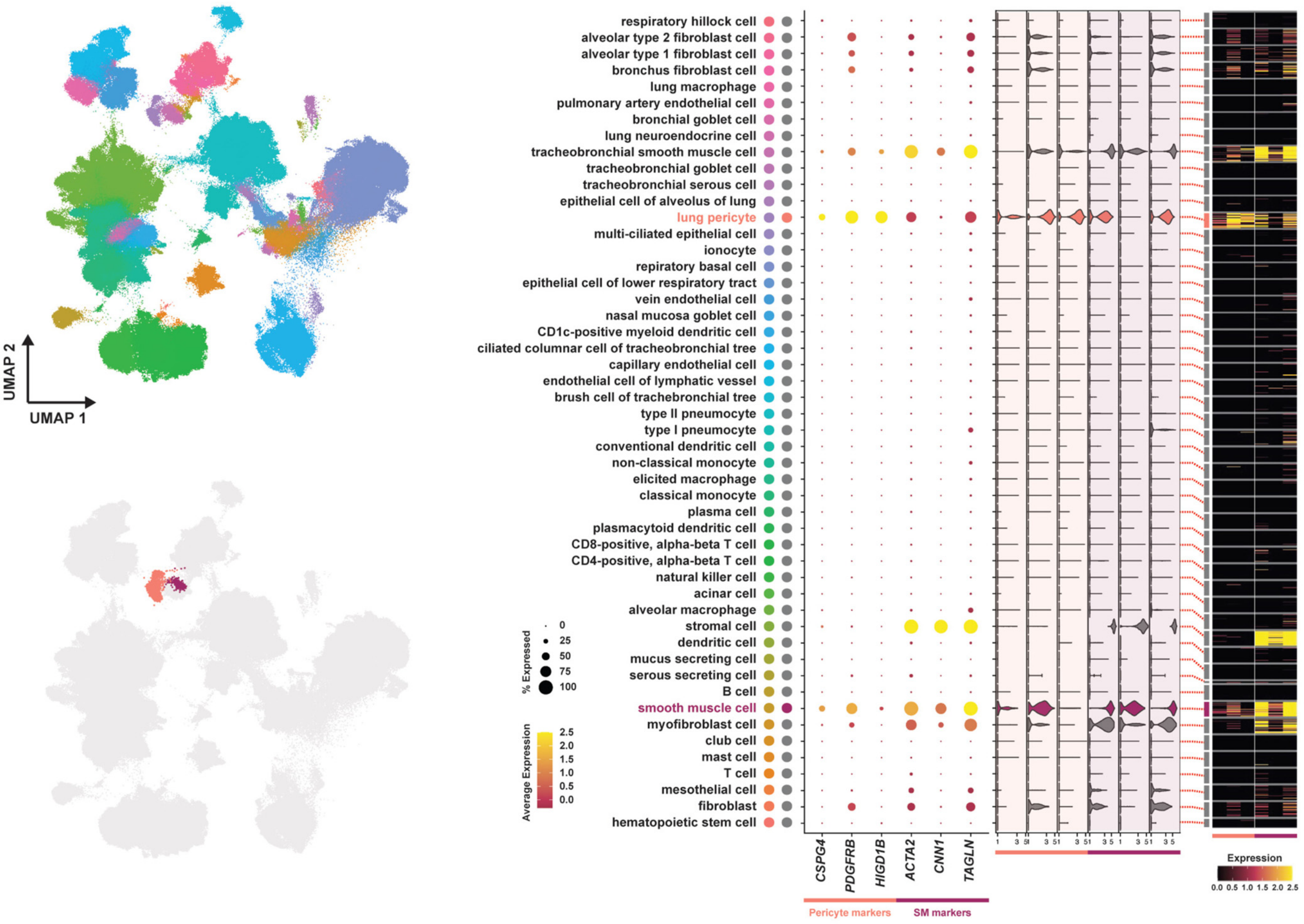

B

	lung pericyte vs Others				smooth muscle cell vs Others			
	Ave log2FC	padj	pct.1	pct.2	Ave log2FC	padj	pct.1	pct.2
CSPG4	1.555	padj < 1E-04	41.1%	1.0%	0.525	padj < 1E-04	36.9%	1.2%
PDGFRB	3.603	padj < 1E-04	91.1%	2.5%	1.944	padj < 1E-04	76.4%	3.0%
HIGD1B	3.239	padj < 1E-04	85.6%	0.5%	0.455	padj < 1E-04	18.7%	1.0%
ACTA2	2.467	padj < 1E-04	68.6%	6.7%	4.752	padj < 1E-04	99.1%	7.0%
CNN1	0.271	padj < 1E-04	10.2%	1.0%	1.991	padj < 1E-04	75.9%	1.0%
TAGLN	2.398	padj < 1E-04	77.5%	12.9%	5.323	padj < 1E-04	99.3%	13.2%

HLCA (n = 506,665 cells), lung pericyte (n = 3,032), smooth muscle cell (n = 556), Others (n = 503,633): HLCA excluding lung pericyte, & Others (n = 506,109): HLCA excluding smooth muscle cell

© The Author(s)

A

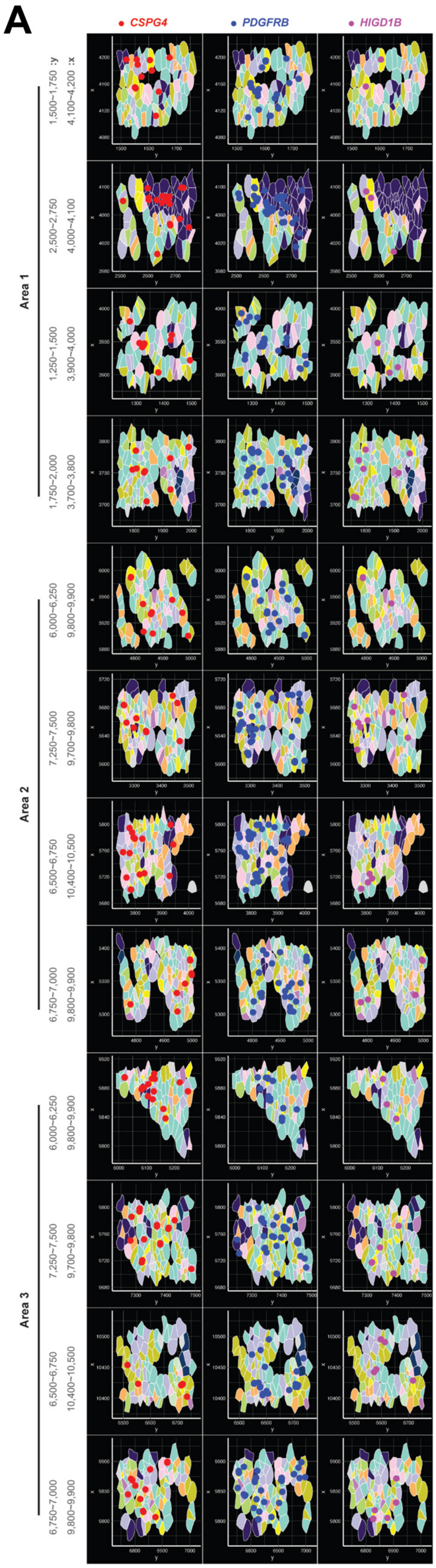

B

		Pericyte (+)	Pericyte (-)
CSPG4	(+)	30	75
	(-)	36	1,171

Sensitivity	45.5%
Specificity	94.0%
(+) predictive value	28.6%
(-) predictive value	97.0%
Accuracy	91.5%

		Pericyte (+)	Pericyte (-)
PDGFRB	(+)	50	179
	(-)	16	1,067

Sensitivity	75.8%
Specificity	85.6%
(+) predictive value	21.8%
(-) predictive value	98.5%
Accuracy	85.1%

		Pericyte (+)	Pericyte (-)
HIGD1B	(+)	23	26
	(-)	43	1,220

Sensitivity	34.8%
Specificity	97.9%
(+) predictive value	46.9%
(-) predictive value	96.6%
Accuracy	94.7%

		SMC (+)	SMC (-)
CSPG4	(+)	21	84
	(-)	85	1,122

Sensitivity	19.8%
Specificity	93.0%
(+) predictive value	20.0%
(-) predictive value	93.0%
Accuracy	87.1%

		SMC (+)	SMC (-)
PDGFRB	(+)	30	199
	(-)	76	1,007

Sensitivity	28.3%
Specificity	83.5%
(+) predictive value	13.1%
(-) predictive value	93.0%
Accuracy	79.0%

		SMC (+)	SMC (-)
HIGD1B	(+)	0	49
	(-)	106	1,157

Sensitivity	0.0%
Specificity	95.9%
(+) predictive value	0.0%
(-) predictive value	91.6%
Accuracy	88.2%

		Pericyte	SMC
CSPG4	(+)	30	21
	(-)	36	85

Sensitivity	45.5%
Specificity	80.2%
(+) predictive value	58.8%
(-) predictive value	70.2%
Accuracy	66.9%

		Pericyte	SMC
PDGFRB	(+)	50	30
	(-)	16	76

Sensitivity	75.8%
Specificity	71.7%
(+) predictive value	62.5%
(-) predictive value	82.6%
Accuracy	73.3%

		Pericyte	SMC
HIGD1B	(+)	23	0
	(-)	43	106

Sensitivity	34.8%
Specificity	100%
(+) predictive value	100%
(-) predictive value	71.1%
Accuracy	75.0%

C

Pericyte vs all other cells					
PC Markers	PPV	NPV	Sensitivity	Specificity	Accuracy
HIGD1B	46.9%	96.6%	34.8%	97.9%	94.7%
CSPG4	28.6%	97.0%	45.5%	94.0%	91.5%
PDGFRB	21.8%	98.5%	75.8%	85.6%	85.1%

SMC vs all other cells					
PC Markers	PPV	NPV	Sensitivity	Specificity	Accuracy
HIGD1B	0.0%	91.6%	0.0%	95.9%	88.2%
CSPG4	20.0%	93.0%	19.8%	92.8%	87.1%
PDGFRB	13.1%	93.0%	28.3%	83.5%	79.0%

 © The Author(s)

◀ **Figure EV2. Statistical analysis of spatial transcriptomic figures are collected from non-diseased lung tissue.**

(**A**) The quantification of mural cell markers was performed using four selected areas from the spatial transcriptomic figure, derived from the three yellow boxes in Appendix Fig. S7. In these analyses, PCs are represented by yellow-colored cells, while SMCs are indicated by dark blue-colored cells. The expression of specific markers is depicted as follows: *CSPG4* is represented by red dots (left), *PDGFRB* by blue dots (middle), and *HIGD1B* by purple dots (right). (**B**) The analysis results are summarized in 2 × 2 tables that display the presence of *CSPG4*, *PDGFRB*, and *HIGD1B* across cell populations. Detailed quantification processes are described in the Methods section. Each table is followed by the corresponding results of the analysis. (**C**) The bottom tables summarize the performance of each marker to distinguish PCs or SMCs from other cells (PC vs all other cells, SMCs vs all other cells, and PCs vs SMCs). TP true positive, FP false positive, TN true negative, PPV positive predictive value, NPV negative predictive value.

© The Author(s)

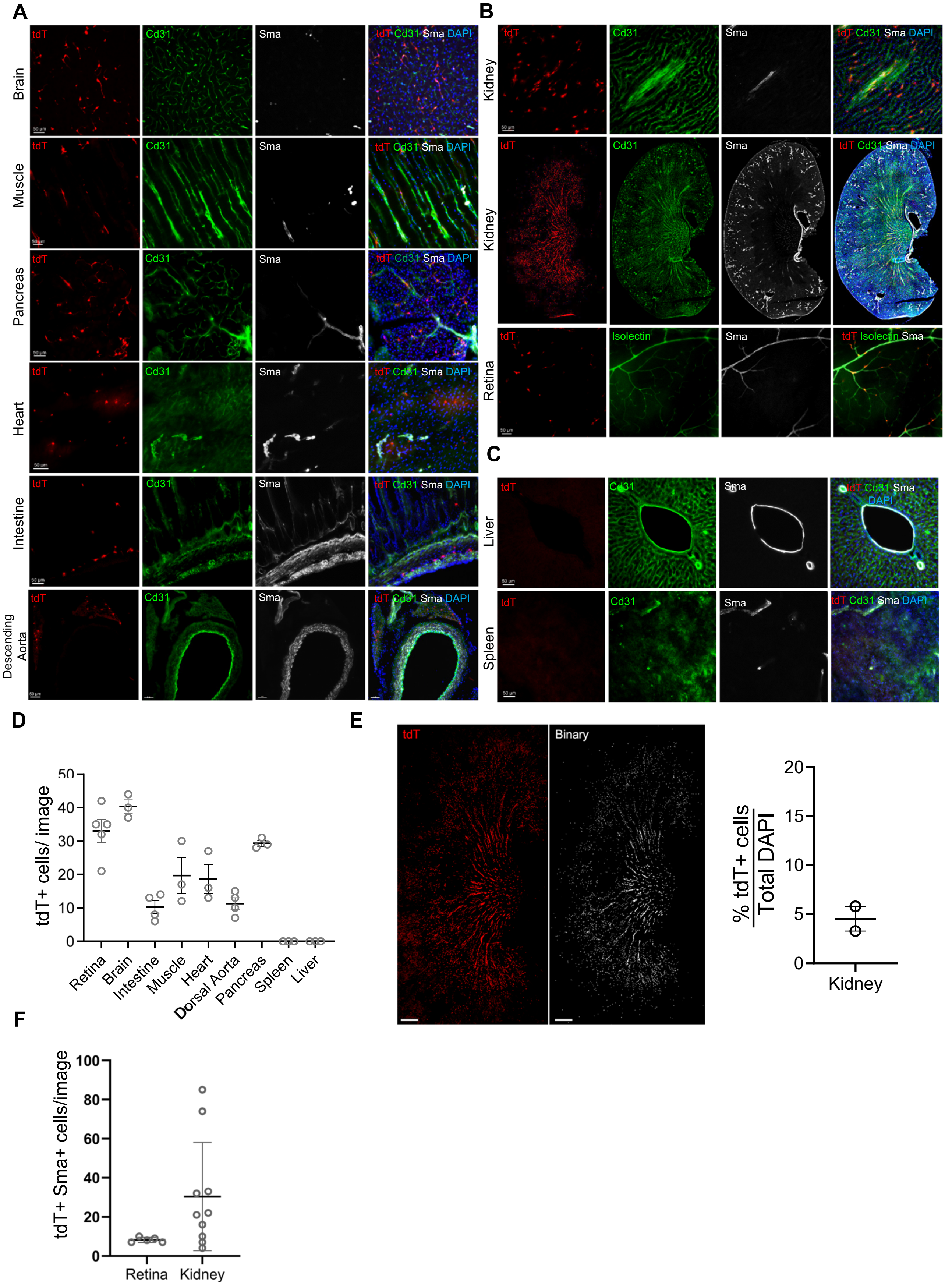
A
B
C
D
E
F
Brain
Muscle
Pancreas
Heart
Intestine
Descending Aorta
Kidney
Retina
Liver
Spleen
tdT
Cd31
Sma
tdT Cd31 Sma DAPI
Isolectin
tdT Isolectin Sma
Binary
tdT+ cells/ image
Retina
Brain
Intestine
Muscle
Heart
Dorsal Aorta
Pancreas
Spleen
Liver
% tdT+ cells / Total DAPI
Kidney
tdT+ Sma+ cells/image
Retina
Kidney

 © The Author(s)

Figure EV3. tdT cells from *Higd1b-tdT*+/− label PCs in other organs in vivo.

(**A**) Stainings of brain, skeletal muscle, pancreas, heart, intestine, and connective tissue around the descending aorta from *Higd1b-tdT*+/− mice for Cd31 (green), Sma (white), and DAPI (blue). tdT endogenous reporter labeled PCs are in red. Note the presence of PCs found within multiple organ systems. Scale bar: 50 μm. (**B**) The kidney (top two panels) and retina (bottom panel) from *Higd1b-tdT*+/− mice were stained for Cd31 (green), Sma (white), and DAPI (blue). tdT+ PCs are in red. Scale bar: 50 μm. (**C**) Liver (top panel) and spleen (bottom panel) from *Higd1b-tdT*+/− mice stained for Cd31 (green), Sma (white), and DAPI (nuclei: blue) reveal an absence of tdT+ PCs (red) in both organs. Scale bar: 50 μm. (**D**) Quantification of tdT+ cells in various organs from *Higd1b-tdT*+/− mice in each image field inspected. Image area inspected = 429.5 μm^2. $N = 3$ for all organs except heart tissues ($N = 2$). Each dot represents an individual image analyzed. Data presented as mean ± SEM. (**E**) tdT+ cells from the kidney taken from *Higd1b-tdT*+/− mice (left) alongside binary immunofluorescence (middle). Scale bar: 50 μm. Quantification of tdT+ cells from the whole cell population identified with DAPI staining is displayed. $N = 2$, with each dot represents an image taken by an individual animal. (**F**) Percentage of tdT+, Sma+ cells per image field from the retina and kidney obtained from *Higd1b-tdT*+/− mice. Inspected image area = 429.5 μm^2. $N = 3$ for each group, with each dot representing an individual image analyzed. Data presented as mean ± SEM.

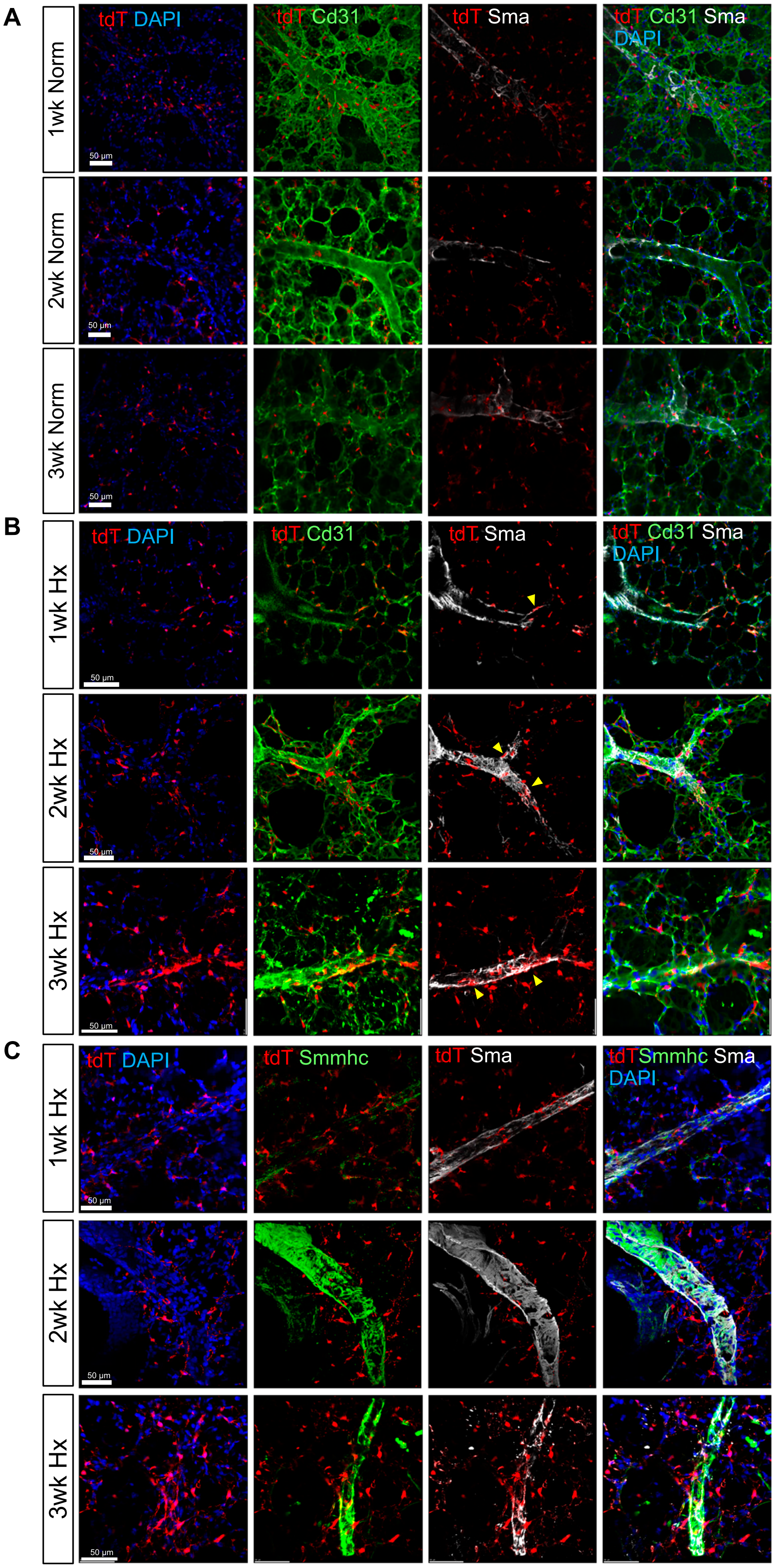
A
1wk Norm
2wk Norm
3wk Norm
tdT DAPI
tdT Cd31
tdT Sma
tdT Cd31 Sma DAPI
50 µm
B
1wk Hx
2wk Hx
3wk Hx
C
tdT Smmhc
tdT Smmhc Sma DAPI

 © The Author(s)

Figure EV4. Lineage tracing shows that tdT+ cells accumulate in muscularized distal arterioles by different hypoxic exposure times.

(**A**) tdT+ PCs from *Higd1b-tdT+/−* mice showing Sma negative tdT+ PCs(red) located in the parenchymal region and distal arterioles after 1, 2, and 3 wks of normoxia. Cd31 in green and Sma in white and DAPI in blue. (**B**) Representative images of PCLSs from *Higd1b-tdT+/−* mice show tdT+ PCs (red) on distal arterioles (Cd31, green) coexpressing Sma (white) after exposure to 1, 2, and 3 wks of Hx. Yellow arrowheads highlight the accumulation of PCs on remodeled distal arterioles that coexpress Sma after Hx exposure. Scale bar: 50 µm. (**C**) Accumulation of tdT+ PCs (red) in muscularized distal arterioles, with staining for SMC markers Smmhc (green) and Sma (white) after 1, 2, and 3 wks of Hx. DAPI: blue. Scale bar: 50 µm. Source data are available online for this figure.

© The Author(s)

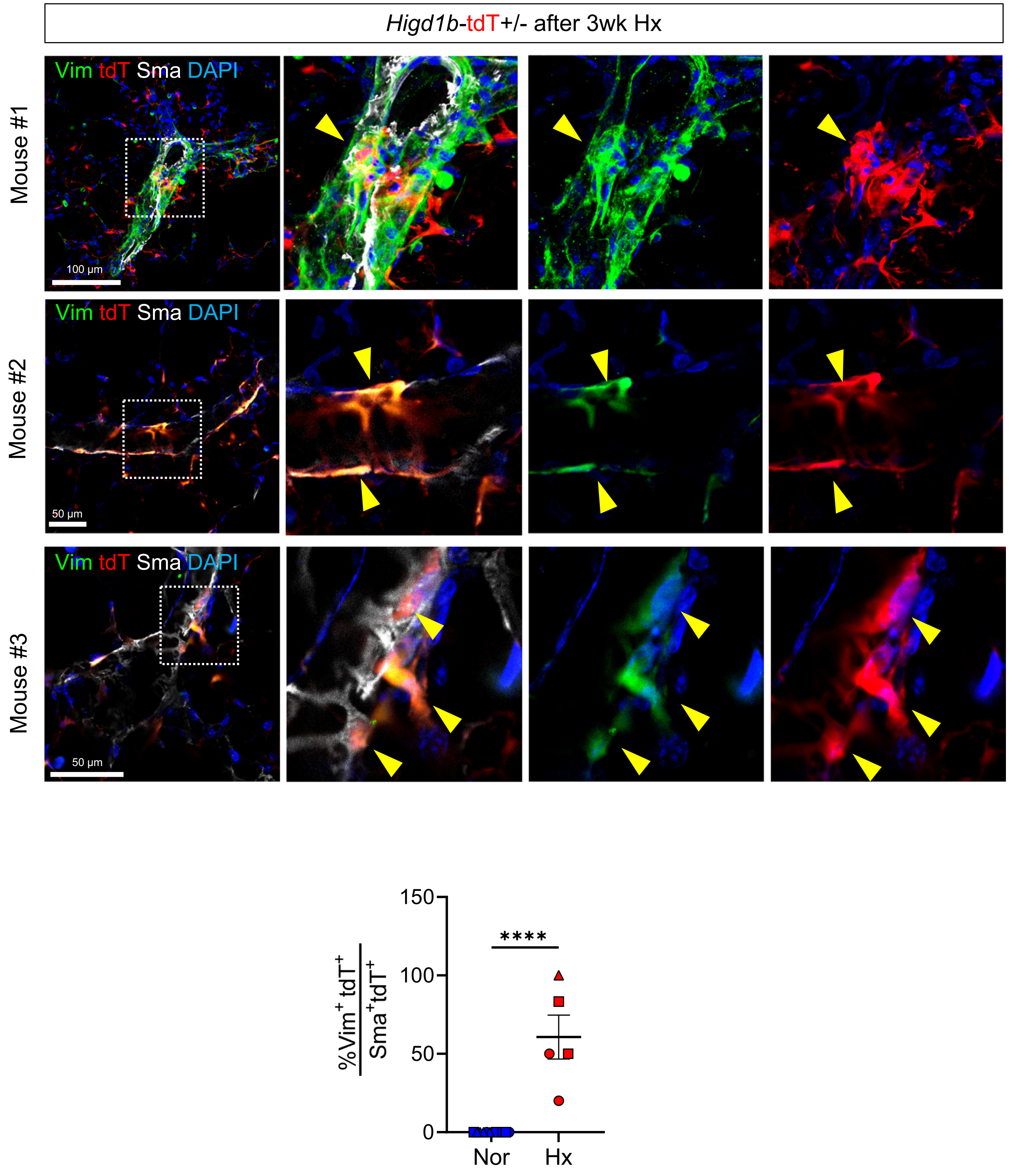
Higd1b-tdT+/- after 3wk Hx
Mouse #1
Mouse #2
Mouse #3
Vim tdT Sma DAPI
Vim tdT Sma DAPI
Vim tdT Sma DAPI
100 μm
50 μm
50 μm
%Vim+ tdT+ / Sma+tdT+
150
100
50
0
****
Nor
Hx

 © The Author(s)

Figure EV5. Chronic Hx results in upregulation of Vim in Type 2 PCs accumulated on distal arterioles.

PCLSs from three *Higd1b-tdT+/−* mice demonstrate the upregulation of Vim (green) in Type 2 PCs (red) accumulated on distal arterioles. Yellow arrowheads highlight the morphological changes of Type 2 PCs and the coexpression of Vim. Sma (white). DAPI (blue). Scale bar: 50 μm. The bottom figure shows the quantification of tdT+ cells coexpressing Vimentin in normoxic and Hx *Higd1b-tdT+/−* mice. $N = 3$ for each group. Each dot represents an individual image analyzed. Data is presented as mean ± SEM. Statistical analysis is performed with an unpaired t-test. ****$P < 0.0001$ indicates statistical significance. Source data are available online for this figure.

© The Author(s)
